# Motivations to connect with like-minded audiences increase partisan sharing on social media

**DOI:** 10.1093/pnasnexus/pgaf197

**Published:** 2025-06-14

**Authors:** Antoine Marie, Michael Bang Petersen

**Affiliations:** Department of Political Science, Aarhus University, Bartholins Allé 7, DK-8000 Aarhus C, Denmark; Département d’études cognitives, Institut Jean Nicod, ENS, EHESS, PSL University, CNRS, 75005 Paris, France; Department of Political Science, Aarhus University, Bartholins Allé 7, DK-8000 Aarhus C, Denmark

**Keywords:** audience, followers, misinformation, reputation, social media

## Abstract

Social media have been accused of facilitating the spread of partisan, hostile, and false news in ways that may foster ideological and affective polarization. Prior research has emphasized that individuals are motivated to selectively share partisan news if it promotes their political convictions or identity. Using a field study of news-sharing behavior on Twitter (study 1: *n* = 1,308) and two online experiments (study 2: *n* = 1,735; study 3: *n* = 1,637), we show that perceptions of the audience as being politically aligned is a key driver of partisan sharing. Partisan social media users selectively share congruent political news based on anticipation of positive reactions from like-minded audiences and refrain from sharing news to avoid upsetting politically dissimilar followers. The evidence for audience effects on partisan sharing in the field study 1 is mostly clear for real news, and it is compelling for both true and false news in the experimental studies 2 and 3. With study 3, we further show that partisan sharing is partly driven by social motivations to connect with, be liked by, and mobilize politically like-minded audiences, in parallel with intrinsic factors. This suggests that the formation of “echo chambers” may trigger social motives that further the sharing of polarizing and false claims.

Significance Statement Fears about social media amplifying political polarization and misinformation spread have risen in recent years. Using a field study of actual news-sharing behavior on US Twitter in 2019 and two online experiments, we demonstrate that social media users engage in partisan sharing of news in ways that are amplified when the audience is politically aligned with them. This partisan sharing of news is motivated by intrinsic properties of the news—such as whether they are perceived as true and politically aligned—but also by social goals—such as being liked by and mobilizing followers. It thus appears that partisan communication online is bolstered by motivations to connect with one's audience in ways that can spread polarizing messages.

## Introduction

Concerns about a negative impact of social media on partisan polarization and public opinion in democracies have centered around two phenomena (1–5). First, the existence of homogenous online networks of politically like-minded users, suspected of worsening opinion accuracy and affective polarization via “echo chamber” effects (6–11). Second, users’ tendency to be highly selective in their sharing of political news by preferring to post stories that promote their political convictions and partisan interests, even if such news demonizes political opponents or makes fabricated claims (5, 12–19).

To explain partisan sharing, most extant work emphasizes *intrinsic* (i.e. content-specific) factors. That is, how properties of a political news item or claim fit a user's convictions or beliefs and motivate decisions to communicate it. For instance, some scholars highlight that most citizens value information accuracy, and strive to share information they see as true (16, 20). Others argue that citizens are motivated to believe and share more messages that “affirm their social identity,” i.e. that portray the political ingroup positively and reinforce their self-esteem (21). Similarly, being attitudinally extreme on and moralizing a policy issue (e.g. abortion, gun control) have been found to magnify partisan sharing (14).

Here, we move beyond a focus on intrinsic factors by demonstrating that the presence of a like-minded audience is a key condition of partisan sharing and that it activates *social* or *relational* concerns to connect with such audiences. We show that people are more likely to engage in partisan sharing of political headlines on social media when the ingroup is watching and that they do so to be liked by and influence their audiences, not just because they personally like or believe the political messages to be true.

We take as our premise that humans are hyper-social and groupish animals, inclined to join and form groups and to compete against rival coalitions to promote their interests and worldviews (22–24). On divisive societal and political issues, which our minds construe as modern instances of group conflict (25, 26), individuals tend to exchange information not just because it is thought to be true, but because it is seen as a tool of influence on others. One recurrent example of this is attempts to enhance one's *social standing* in the eyes of others. Early work on political discussions and rumors (27) in the real world has found that individuals selectively express opinions that are approved by their social circles and refrain from expressing thoughts that may damage their reputations, sometimes at the price of losing track of the nuances of social issues (28–32). Similar dynamics operate online (14, 33, 34). For instance, to signal trustworthiness, Twitter and Facebook users broadcast the pronouns they want to be addressed with; express outrage at celebrities’ statements; or customize their profile pictures to indicate solidarity with causes like transgender rights or victims of terrorist attacks (e.g. after the Bataclan attacks in Paris in 2015).

In parallel, a frequent social goal of political communication is to *get others to act*, for instance, to engage against perceived societal issues and villains (3, 26, 34–37). Political narratives and headlines are often threat based, pointing to social problems to be solved or accusing political opponents of harming the common good, and are thus used as tools of mobilization (e.g. a headline such as “Nancy Pelosi in custody after tax evasion accusations” can be spread to mobilize pro-Republican citizens against Democrats).

In line with these ideas, a stream of research has explored social motivations for spreading political messages online and audience influences on social media communication. Marwick & Boyd (38) used text analysis to show that Twitter users know that the social identity of their followers is often unclear and that they have to imagine their reactions before writing something publicly. Other studies demonstrated that fear of discrediting oneself as a source of information contributes to keep false news-sharing levels low (20), and that positive misbeliefs are often shared out of motivations to signal kindness to and bond with online friends (39). Exploiting a dataset from 2020 Twitter, Lawson et al. (40) observed that users who re-share hyper-partisan posts from like-minded followers were better able to maintain social connections with them than those who did not reshare, suggesting that echoing one's friends’ communication helps maintain online ties. Similarly, Ren et al. (41) provided experimental evidence that expectations of “likes” and comments from social media audiences increase chances of expressing conspiracy theories.

### Our approach and contribution

Despite advances in the study of the social motivations for sharing online content, we know of no prior studies exploring in depth how the perceived *political composition* of one's audience shapes decisions to share partisan news online. Our key contribution here is thus to examine a premise neglected by previous studies. Namely, that for social motivations to engage in partisan sharing to be activated, *users must represent how their audience will react based on an assessment of their political orientation*. Human communication relies on expectations of relevance, relevance which depends on the audience's prior beliefs, desires, and interests (42, 43). Accordingly, decisions to spread political messages should be determined by the triangular relationship between a user anticipating her audience's likely reactions to gauge whether sharing a political claim (e.g. a news story) will produce beneficial social effects, before deciding to share it or not. The overarching view we want to defend is that partisan sharing does not occur in a vacuum: social motivations to connect with and influence like-minded audiences underpin social media users’ decisions to share political content, in parallel with intrinsic motivations like the perceived truth of a piece of news or its fit with moral convictions.

As regards the outcomes investigated, our focus is on decisions and intentions to share partisan news stories on social media in the polarized United States of the Biden presidency, as an archetype of political expression in contexts of sociopolitical conflict. We now turn to our predictions. In line with extant research, we expect (H1) users to be much more likely to share partisan news stories that are ideologically congruent with their own partisanship and attitudes than incongruent ones (12, 14, 19). By congruent news, we mean news stories that report on an event or societal fact that somehow advances a political side's agenda, or points at a threat that partisans of that political side would typically find important and credible (e.g. a news story mocking Joe Biden's old age for Republicans). Incongruent news, in contrast, is defined as news items that would typically satisfy those goals among political opponents. Crucially, we anticipate variation in the political composition of respondents’ audience to modulate the size of partisan sharing. We predict (H2) that the sharing of politically congruent news increases in the presence of ingroup audiences—whom it makes sense to signal to, court, mobilize, etc.—when compared with outgroup audiences.

Our studies include a wide range of partisan news varying along the dimensions of congruency (studies 1–3), truth/plausibility (studies 1–3), and hostility (studies 2 and 3). Using data from the US Twitter (study 1), we identify associations consistent with the notion that audience influences on shares of partisan news are taking place on actual social media. Using experimental approaches, we then show that the sharing of congruent headlines of all types (true, false, hostile or not) is boosted by the presence of like-minded audiences (studies 2 and 3). Moreover, an observational analysis of the motivations for sharing partisan news (study 3) finds not only that perceived accuracy and congruence with moral convictions are important reasons to disseminate stories online, but also—consistent with the argument put forth here—that social motives like being praised by like-minded audiences play a central role.

Overall, we demonstrate that the online presence of like-minded followers amplifies the sharing of partisan (mis)information on social media through a combination of intrinsic and social motivations.

## Study 1: observing audience effects on the US Twitter

Study 1 aimed to provide an initial and externally valid test of the idea that users’ beliefs about the likely reactions of their social media followers influence the political slant of the news they share. Study 1 relied on a reanalysis of observational data on news shared on the US Twitter in 2019 collected by Osmundsen et al. (15).

### Hypotheses

Study 1 was exploratory and its analyses were not preregistered. Based on past research, we expected users to favor sharing congruent over incongruent news (H1), whether real or false. We also expected perceptions of one's Twitter followers as being more politically like-minded (i.e. more clearly from the political ingroup) to predict more shares from news domains publishing news congruent to the respondent and their audience (H2), whether real or false. In contrast, we had no expectation about how the perceived like-mindedness of one's Twitter followers would affect shares of real and false news incongruent to the respondent and their audience.

### Methods

#### Consent and ethics

All participants recruited in the studies reported in this paper gave written informed consent. All studies complied with the Danish Code of Conduct for Research Integrity. As specified in the Danish law of the National Committee on Health Research Ethics (§14.2), surveys “that do not involve human biological material” are exempt from further ethical review. See letter of ethical review by the head of the political science department of Aarhus University in Supplementary Material, B.

#### Participants and data

The dataset of study 1 was composed by linking news-sharing behavior on the US Twitter to a YouGov survey asking participants about their partisanship and their perceptions of the political leaning of their Twitter followers. The YouGov survey lasted about 20 min and was run between mid-December 2018 and mid-January 2019. The survey also asked respondents for their Twitter handles, for permission to scrape data on their publicly available Twitter activity, and to link this activity with the responses from the YouGov survey. Twitter data from consenting participants were scraped around early March 2019, and includes news domains tweeted or retweeted in the months and years until that time.

Of the 27,000 US Twitter users invited by YouGov to participate in the survey, only a small minority accepted the invitation, answered all questions, and provided an identifiable Twitter handle. Respondents were asked about their perceptions of the political orientation of their followers in the second one of the six waves. As a result, our final sample size in study 1 (*n* = 1,308) is smaller than that of Osmundsen et al. (15) (*n* = 2,337). Due to nonrandom attrition, our sample does not accurately reflect the general US population: 14.4% respondents were aged between 18 and 29 years old (yo), 35.4% between 30 and 44 yo, 42% between 45 and 64 yo, and 0.08% were over 65 yo. In addition, 49% of respondents were female, 68.8% Democrats, and 31.2% Republicans (no Independents were kept in the data, like in the other studies in this paper). Still, these characteristics approximately match those of Twitter users, and of samples analyzed in other high-quality investigations of partisan behavior on X/Twitter (15, 44, 45). To alleviate possible concerns about data quality, let us highlight that Appel & Haas (46) recently reviewed the Twitter sample from Osmundsen et al. (15). Their analyses confirm that people who provided access to their Twitter activity reflect the sample of individuals who took the YouGov survey and provided valid Twitter handles.

#### Ethics

All participants recruited in the studies reported in this paper gave written informed consent. Research took part at Aarhus University as part of the project Research on Online Political Hostility.

#### Design and procedure

The number of times a Twitter user shared a link to a political news domain constitutes our dependent variable. Following best practices, we define sharing from “false news sources” as tweets or retweets by panel members of URLs to web domains which have been identified as publishing factually false news stories in the past. As in Osmundsen et al.’s (15) investigation, the list of false news domains was established by cross-referencing all the tweet URLs against a list of 608 false news sources previously constructed by journalists and scholars (47). “Real news sources” were defined by cross-referencing tweet URLs against a list of real news sources defined by the AllSides organization, which seeks to “strengthen our democratic society with balanced news, media bias ratings, [and] diverse perspectives” (www.allsides.com/media-bias/). A domain or source was defined as publishing news congruent to Democrat-leaning users if it had been identified as publishing pro-Democrat real or false news, and incongruent for Democrat users if it had published pro-Republican real or false news. The reverse was true for pro-Republican news domains.

Our distinction between real vs. false news domains is based on actual online behaviors and thus avoids potential biases associated with self-report recalls of online activity. Still, it is important to keep in mind that our classification of news domains does not rest on an examination of each one of the specific news items shared. It is based on whether the publisher is known to have produced false news in the past or not, as is commonly done in research on social media behavior (13, 44, 48).

The YouGov survey probed respondents on their own partisanship on a 7-point scale (from [1] “Strong Democrat” to [7] “Strong Republican” with [4] “Independent”), as part of demographic questions. Its wave 2 then asked participants about their perceptions of the political orientation of their Twitter followers, also on a 7-point scale (“Let us think about the people who follow you on Twitter. Do you think most of them identify politically as…”: from [1] “Strong Republican” to [4] “Moderate” to [7] “Strong Democrat”). This latter measure of followers’ political slant was transformed into a measure of perceived political like-mindedness of one's followers by reverse-coding it for Republicans.

### Results

Our dependent variable, news shared, was subject to zero inflation (76.8% respondent did not share any URL from the political news websites we inventoried) and overdispersion (Mean = 8.6 < Variance = 4,114). See the histogram of the distribution of shares from real and false news domains in Supplementary Material, E.1. Consequently, influences of followers’ like-mindedness on shares from news domains were modeled by default using zero-inflated negative binomial (ZINB) and negative binomial (NB) regressions on the whole dataset (as opposed to Poisson regression, not recommended with zero inflation and overdispersion (49).^a^ For robustness purposes and on reviewers’ requests, we also report regression coefficients of shares from ZINB models while excluding the 1% greatest news sharers (of fake or real news, depending on the subsets analyzed), and from Poisson regression models. See Supplementary Material, E.4 for regression tables and Supplementary Material, E.3 for groupwise numbers of shares. Note that, as shown in Table 1, ZINB and NB models consistently obtained better BIC scores than Poisson regressions. Still for robustness, tests of associations were performed using two distinct classifications of real news domains (using AllSides’ and replicated with Bakshy et al.’s (50) classification) and false news domains (using Allcott et al.’s (47) and replicated with Grindberg et al.’s (44) classification). Those classifications were already available in Osmundsen et al.’s (15) data. As preregistered and like in all studies reported in this paper, respondents who reported as being politically Independents were removed from the dataset prior to data analysis.

**Table 1.** Coefficients, 95% CIs and significance of the associations between shares from real and false news domains on Twitter and followers’ perceived like-mindedness in study 1.

	Associations between shares and followers’ like-mindedness (*b*_exponentiated_)	
	Congruent news	Incongruent news
Real news (AllSides’ main classification)		
ZINB	**1.47** [1.26, 1.71], *P* < 0.001, BIC = 7,629	**0.74** [0.65, 0.85], *P* < 0.001, BIC = 3,355
ZINB without 1% outliers	**1.30** [1.14, 1.47], *P* < 0.001, BIC = 6,953	**0.80** [0.70, 0.92], *P* = 0.002, BIC = 3,323
Negative binomial	**1.44** [1.21, 1.70], *P* < 0.001, BIC = 7,969	**0.75** [0.65, 0.87], *P* < 0.001, BIC = 3,567
Poisson	**1.40** [1.39, 1.42], *P* < 0.001, BIC = 154,915	**0.58** [0.57, 0.60], *P* < 0.001, BIC = 15,679
Real news (Bakshy et al.’s (50) alternative classification)		
ZINB	**1.39** [1.19, 1.64], *P* < 0.001, BIC = 7,491	**0.71** [0.62, 0.83], *P* < 0.001, BIC = 3,370
ZINB without 1% outliers	**1.26** [1.10, 1.43], *P* = 0.001, BIC = 6,813	**0.76** [0.65, 0.88], *P* < 0.001, BIC = 3,345
Negative binomial	**1.36** [1.14, 1.61], *P* < 0.001, BIC = 7,792	**0.72** [0.61, 0.84], *P* < 0.001, BIC = 3,548
Poisson	**1.36** [1.34, 1.37], *P* < 0.001, BIC = 148,262	**0.61** [0.59, 0.62], *P* < 0.001, BIC = 14,941
False news (Allcott et al.’s (47) main classification)		
ZINB	0.47 [0.22, 1.02], *P* = 0.055, BIC = 1,298	0.83 [0.50, 1.38], *P* = 0.477, BIC = 404
ZINB without 1% outliers	**0.54** [0.33, 0.89], *P* = 0.016, BIC = 825	0.83 [0.50, 1.38], *P* = 0.477, BIC = 404
Negative binomial	0.98 [0.70, 1.38], *P* = 0.917, BIC = 1,270	0.68 [0.39, 1.13], *P* = 0.112, BIC = 362
Poisson	**1.36** [1.29, 1.43], *P* < 0.001, BIC = 11,173	0.76 [0.58, 1.01], *P* = 0.060, BIC = 430
False news (Grindberg et al.’s (44) alternative classification)		
ZINB	**1.70** [1.07, 2.70], *P* = 0.026, BIC = 1,263	**0.56** [0.35, 0.92], *P* = 0.021, BIC = 443
ZINB without 1% outliers	**1.84** [1.19, 2.85], *P* = 0.006, BIC = 817	**0.53** [0.32, 0.90], *P* = 0.019, BIC = 416
Negative binomial	1.29 [0.93, 1.81], *P* = 0.128, BIC = 1,238	0.76 [0.46, 1.27], *P* = 0.296, BIC = 419
Poisson	**1.32** [1.25, 1.40], *P* < 0.001, BIC = 8,963	**0.63** [0.51, 0.79], *P* < 0.001, BIC = 663

All models include the same variables and adjust for sex, age, education, race, income, number of Twitter followers and user's partisanship. Coefficients are in bold when significant at *P* < 0.05.

**Fig. 1.**
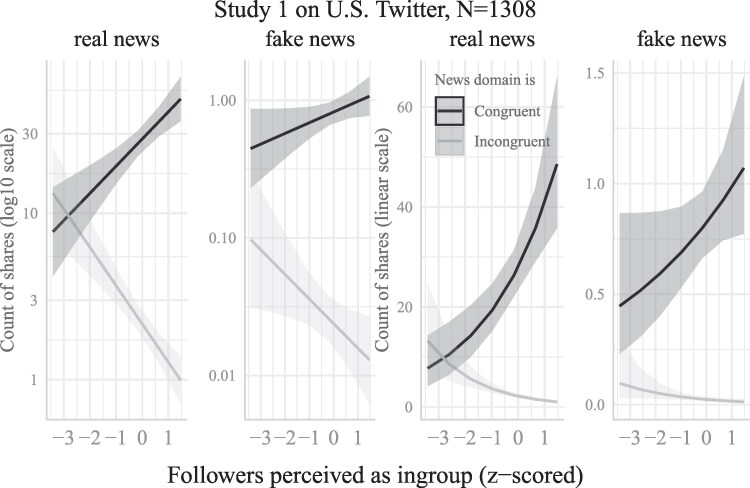
Predicted counts of shares of partisan news from domains publishing real and false news ideologically congruent vs. incongruent to respondents’ partisanship, as a function of the political like-mindedness of respondents’ Twitter followers in study 1 (*z*-scored, high scores to the right). Shares are plotted using log_10_ (left panels) and linear scales (right panels). Data displayed here was fit on all the data available with NB regression. 95% CIs are shown as grayed areas.

Associations between followers’ like-mindedness and real and false news shared on Twitter are displayed in Fig. 1 and Table 1. From the models (Table 1), we report the exponentials of the log odds coefficients, which are expressed in original units and are to be interpreted as multiplicative effects.^b^ All models of shares include respondents’ age group, sex, race, education, household income, number of Twitter followers, and users’ partisanship as controls. Covariates and control variables were *z*-scored prior to analyses, but not the count of news shared, our dependent variable. Regression coefficients are provided with 95% CIs, and *P*-values.

Twitter users mostly reported being followed by other users who share their partisanship, entailing high homophily of their online network, as documented by previous research (51) (Supplementary Material, E.2).

Overall, shares from false news domains (*M* = 0.39) were *much* rarer than shares from real news domains (*M* = 17) by a factor of 43 (NB fit: *b*_log odds_ = −3.78, [−4.34, −3.21], *b*_expon_. = 0.022, [0.013, 0.040], *P* < 0.001; see Supplementary Material, E.3). This observation corroborates prior studies finding that people share very few fake news overall (8, 15, 48).

#### Associations between audience composition and shares from congruent news domains

As expected, there were clear associations between the perceived political orientation of one's Twitter followers and decisions to share news from both real and false news domains. They are reported in Table 1 with several fitting procedures and visualized in Fig. 1. Simple slope analyses revealed that when followers were perceived as more ideologically like-minded (i.e. from the political ingroup), Twitter users shared more congruent news from *real* news domains. Associations were consistently positive across fitting procedures (ZINB, NB, and Poisson), which criterion was used to classify as a real news domain (AllSides or Bakshy et al. (50)), and were robust to exclusion of the 1% greatest sharers. Using a NB model to estimate the predicted values, mean shares of congruent real stories at the minimum of the followers’ like-mindedness continuum, −3.41, was 7.58 [4.43, 12.96] whereas it climbed to 47.55 [36.40, 62.12] at the maximum, 1.47.

Shares of congruent news from *false* news domains, in contrast, had less clear associations with the degree of like-mindedness of the followers. Associations were positive while using a Poisson fit on Allcott et al.’s (47) classifications of false news domains, and positive while using ZINB, ZINB without 1% outliers, NB, and Poisson fits on Grindberg et al.’s (44) classification. However, associations between followers’ like-mindedness and shares of congruent false news turned out tendentially negative when using ZINB fits on Allcott et al.’s (47) classification.

#### Associations between audience composition and shares from incongruent news domains

When followers were perceived as more ideologically like-minded, Twitter users consistently shared less incongruent news from *real* news domains. Associations were consistently negative across fitting procedures (ZINB, NB, and Poisson), which criterion was used to classify as a real news domain (AllSides or Bakshy et al. (50)), and were robust to exclusion of the 1% greatest sharers. Average shares of incongruent stories from real news domains at followers’ like-mindedness’ minimum were 10.71 [6.18, 18.56] vs. at its maximum 0.98 [0.74, 1.30] (predicted values from NB model).

Incongruent news shared from *false* news domains tended to be negatively correlated with followers’ like-mindedness. However, negative coefficients only reached significance using the Grindberg et al.’s (44) classification using ZINB and Poisson fits, but never when using Allcott et al.’s (47) classification.

Considering the above trends together, it clearly appears that Twitter users do not engage in partisan sharing to the same degree *regardless* of who is reading or watching them. As illustrated by Fig. 1, shares of congruent news are higher than shares of incongruent news—especially for real news—as long as users perceive their audience as being overwhelmingly like-minded to slightly uncongenial (i.e. as falling within the [−2, 1] range of the audience like-mindedness scale approximately). But when followers are perceived as overwhelmingly composed of out-partisans, users do *not* engage in partisan sharing. This is most apparent on shares from real news domains, where users shared equal amounts of incongruent and congruent news, as if to cater to out-partisans’ views (at −3.41, the minimum of the follower's like-mindedness variable, users shared an average of 7.58 [4.43, 12.96] congruent real news stories and an average of 10.71 [6.18, 18.56] incongruent news stories). Similarly, as regards false news domains, congruent news was shared only marginally more often (*M* = 0.24 [0.06, 0.89]) than incongruent news when the audience was overwhelmingly made up of out-partisans (*M* = 0.11 [0.02, 0.59]).

Finally, we examined the associations between the political slant of the followers and Twitter users’ propensity to share news from domains that are politically congenial to those followers (irrespective of whether the domain is politically congruent to the user or not). This latter variable captures the propensity to make sharing choices that align with the political values of the network. This variable takes high values when, for instance, a user shares a story from a pro-Democrat domain to pro-Democrat followers, whether the story is congruent to the user (she is Democrat) or incongruent to her (she is Republican). The variable takes low values when, for instance, the user shares a story from a pro-Democrat domain to pro-Republican followers, whether the story is congruent to the user (she is Democrat) or incongruent to her (she is Republican). We ran this analysis, which ensures higher power, on news shared from real and false news domains separately using the AllSides (real news) and Allcott et al.’s (47) (false news) classifications. Because it involves stacking shares of news congruent and incongruent to each respondent together in the data (entailing repeated observations), clustering robust standard errors around participants’ IDs was required.

This analysis confirms that higher degrees of political congeniality of a news domain to a respondent's followers predict more shares of *real* news stories (ZINB fit: *b*_expon._ = 3.49 [3.06, 3.99], *P* < 0.001, without 1% outliers*: b*_expon._ = 1.70 [1.53, 1.89], *P* < 0.001; Poisson fit: *b*_expon._ = 2.58 [2.23, 2.99], *P* < 0.001). Similarly, higher political congeniality of a news domain to a respondent's followers predicts more shares of *false* news stories (ZINB fit: *b*_expon._ = 3.13 [2.03, 4.83], *P* < 0.001, without 1% outliers: *b*_expon._ = 1.43 [1.03, 1.97], *P* = 0.03; Poisson fit: *b*_expon._ = 2.65 [2.05, 3.43], *P* < 0.001). Note that associations are always statistically significant for both real and false news domains even when excluding the 1% greatest sharers and regardless of regression type. See the end of Supplementary Material, E.5 for regression tables.

### Discussion

Using data on actual news sharing on the US Twitter from 2019, study 1 demonstrates that respondents who believed they had faced a politically like-minded followership were more likely to engage in partisan sharing. Perceptions of one's online audience as being more like-minded were associated with more sharing of real news congruent to respondents and their followers. In contrast, perceptions of one's audience as being more like-minded tended to be associated with decreased sharing of real news incongruent to respondents and their followers.

We note that although associations between followers’ like-mindedness and shares followed similar trends for false news, those were less conclusive. Associations with congruent false news sharing were inconsistent across fitting procedures and domain classifications. Associations with incongruent false news were more consistently negative but only reach significance using one of the two domain classifications. Overall, we suspect that the fact that fake news was shared much less often than real news on Twitter made the associations underpowered and harder to estimate.

Study 1's observational design cannot rule out the possibility that the above associations may be partly explained by Twitter users choosing to follow those accounts that share politically congenial content. Still, as suggested by the clear experimental audience effects found in studies 2 and 3 below, it is likely that partisan sharing in our Twitter sample was, at least partly, caused by genuine effects of audience composition on sharing decisions.

## Study 2: experimental demonstration that partisan sharing is amplified by audiences’ like-mindedness

Study 2 experimentally demonstrates that the perceived political orientation of one's social media audience influences sharing of partisan news. This online survey manipulated whether participants had to imagine sharing news to a politically ingroup vs. outgroup audience on Facebook, before probing them on their intentions to share diverse partisan news stories.

### Preregistered hypotheses

All the materials, hypotheses, sample size and analyses for study 2 were preregistered at: https://osf.io/kvu7h/?view_only=f94af4b4a8ff40e1a24d5f265346b726. We predicted that respondents would report higher intentions to share politically congruent than incongruent stories, regardless of audience composition (H1). In line with the notion that communication on political issues is driven by relational motivations, we expected this preference for congruent news to be amplified by the presence of an ingroup or like-minded audience. Specifically, we predicted an ingroup audience to increase sharing of politically *congruent* news compared with an outgroup audience (H2). Imagining the presence of an ingroup (rather than outgroup) audience should increase motivations to be praised by like-minded followers by sharing content they like, signaling allegiances, or reminding political friends to support cherished causes. With regard to sharing *incongruent* news, there are two different possibilities. On the one hand, partisans tend to be convinced that the narratives upheld by their opponents are false and/or morally wrong (1, 52). As a result, one could expect participants to be equally unlikely to share stories incongruent to them to outgroup audiences (although outgroup audiences might agree with them) than to ingroup audiences (who would disapprove of the news) (H3a). On the other hand, partisans might still be motivated, to some degree, to try to please outgroup audiences by sharing content congruent to those audiences (but incongruent to themselves as sharers). In this case, intentions to share incongruent news would increase in the presence of an outgroup (vs. ingroup) audience (H3b).

### Methods

#### Participants

A power analysis runs in Gpower suggested that 1,500 participants were required in study 2 to detect a small effect of sharing congruent news to an ingroup vs. outgroup audience of *d* = 0.1 at 80% power (alpha level = 0.05). Although we did not formally preregister this criterion, our goal was to arrive at a balanced sample of ∼50% Democratic and ∼50% Republican respondents. Of the 1,829 US respondents recruited on Prolific who completed the study, and as preregistered, 94 were removed because they were neither Democrat nor Republican or failed a pretreatment attention check (see Supplementary Material, B). Our final dataset comprised 1,735 participants for the analyses (*M*_age_ = 42, SD_age_ = 13.9, 50% women), balanced along partisanship: 50% Democrats and 50% Republicans (see Supplementary Material, F.1 for more information). Study 2 had even better power than initially envisaged.

#### Design

The survey was designed in Qualtrics, lasted 16 min on average and was run in mid-February 2023. After giving informed consent, participants answered demographic questions (income, level of education, age, and race), reported their partisanship on a 1-item scale (from [1] “Strong Democrat” to [6] “Strong Republican,” with [99] “I refuse identification to either party”), and their vote at the last general election. They were also probed on a range of potential personality moderators of audience effects on sharing, not analyzed in this article. Respondents were then randomly exposed to one of two vignettes asking them to imagine sharing political news in a Facebook group that was composed either of Democrat or Republican supporters. After reading one of the two vignettes, each participant saw 24 partisan news items and two true neutral stories (used as within-subject controls), presented in a random order.

Although study 2 relied on hypothetical sharing decisions, previous research (53) has found that self-reported willingness to share political news headlines in online surveys correlates moderately with actual sharing on social media (Pearson's *r* = 0.44). Moreover, the perceived interestingness of a piece of news in survey experiments predicts its success on actual social media (54). Studies based on intentions to share news are now a frequent approach to study the psychology of news use and partisanship (14, 16, 37, 55).

### Materials and procedure

#### Audience manipulation

After reporting their partisanship, respondents read a vignette inviting them to imagine they were navigating a Facebook group whose audience was either made up of strong Democrats or strong Republicans, in a between-subjects design. Our vignettes were designed to provide a believable manipulation of the perceived political orientation of one's social media audience. The Facebook group was presented as being officially dedicated to a hobby like a game or a sport, not political issues, but as having strongly Democratic or Republican users. Our intention in using this framing was to make it plausible that, for instance, a Democrat participant would find herself scrolling on a group dominated by Republican-leaning users (and vice versa). Several studies have shown that communities of online and offline gamers are frequently politicized, with both progressive and conservative leanings, and no less likely to vote on average than the rest of the population (56–59). In both conditions, the audience was described as politically homogenous and strongly slanted: no condition described a politically mixed audience or omitted to describe any audience. Table 2 provides the verbatim of each vignette. The underlined words are the only ones that varied across conditions (but they were not underlined in the study's stimuli). After the experiment was over, we used responses to a 1-item measure of partisanship to determine whether the imaginary audience on the Facebook group was the participant's political ingroup (e.g. Democrats for a Democrat participant) or her political outgroup (e.g. Republicans for a Democrat participant). Since all study respondents had been filtered at the recruitment stage to be either Democrat or Republican in leaning, classifying them as falling in the ingroup or outgroup audience condition was straightforward.

**Table 2.** Vignettes used to vary the imagined political composition of the social media audience in study 2.

Democrat Facebook group (e.g. ingroup audience for Democrats)	Republican Facebook group (e.g. outgroup audience for Democrats)
[All conditions:] *Please read very carefully the following instruction, questions will be asked to you about it:* Imagine that you are navigating on a Facebook group that is dedicated to one of your hobbies (e.g. a game or sport). Most discussions on the group are, of course, related to the hobby. But often discussions about politics also appear, and people frequently share news stories in the group	
You know from reading the stories and the comments that the group's members are quite Democrat. They like to point out that Republican politicians and voters are ignorant, that sexism, racism and transphobia are widespread, etc.	You know from reading the stories and the comments that the group's members are quite Republican. They like to point out that Democrat politicians and voters are ignorant, that immigration, abortion and gun control must be opposed, etc.
[All conditions:] They stand firm on their political beliefs, which they think are the only right ones. They can be pretty mean to people who don't share their values. On the other hand, they really praise those who agree with them. So, if you post and write the kinds of things that they want to hear, you know for sure that they will like you.	

Words underlined are the passages that varied across experimental conditions.

On the page following exposure to the vignettes, respondents were asked to recollect the political composition of the Facebook group they had just been described (“Just to double check, we’ve asked you to imagine you are navigating on a Facebook group that is”: “quite Democrat”; “quite Republican”; “quite religious”; “quite atheist”). To avoid posttreatment bias, participants who failed this audience manipulation check were kept in the data; the goal was simply to gauge attentiveness.

Participants then saw the 26 news stories in a random order, and were asked their willingness to share each one, after which they were invited to give feedback on the study, and the survey ended. Each news item was displayed on its own page, with the willingness-to-share question positioned immediately below. Each news item (see Supplementary Material, C.2–C.5) comprised a headline and a picture chosen to illustrate its content; sources were not visible to focus attention on the claims made by the headlines. The formulation of the intentions-to-share question was adapted to each audience described in the vignettes, to strengthen the audience manipulation: “How likely would you be to share that headline in this Democrat [vs. Republican] Facebook group?” [0] “Extremely unlikely,” [1] “Unlikely,” [2] “Somewhat Unlikely,” [3] “Somewhat Likely,” [4] “Likely,” [5] “Extremely likely.” Choices were displayed horizontally, with high values on the right.

#### Selection of the news items

We selected our news stories based on a pretest study. All the pretested news items (see Supplementary Material, C.1), the pretest data and the script of analysis used to select them are available on OSF at https://osf.io/2xdmy/?view_only=b609eafb21e546cdbf113d3d05485cc7. For the pretest study, the true news items were taken from reliable news sources (e.g. *The New York times*, *The Guardian*, a scientific study found on Google Scholar) and were simultaneously intended to be perceived as being higher in accuracy (or plausibility) than all the false news by independent raters recruited on Prolific. The perceived accuracy question read: “To the best of your knowledge, is this claim in the above headline accurate?” (slider scales from [0] “Extremely accurate” to [100] “Extremely inaccurate” with [50] “Undecided” as default choice). In contrast, the false news stories made fabricated claims and were intended to be perceived as being lower in accuracy (or plausibility) than all the true news by the raters. Some false stories drew on posts that had widely circulated on social media and which had been debunked by fact-checkers from Snopes or Politifact. Others reported events or claims by politicians which we invented while giving them the desired characteristics (e.g. being rather implausible + hostile to a Democratic politician). Both groups of true-high accuracy and false-low accuracy stories were composed by dropping items until we obtained two clearly distinct clusters, one low and the other high in perceived accuracy-plausibility (see Supplementary Material, C.1 for visualizations and statistical tests).

Within each batch of true and false items, half was intended to be perceived as being merely congruent to the beliefs and goals of one political side (e.g. congruent to Democrats), whereas the other half was meant to be perceived as being *hostile* to the outgroup of that political side (e.g. hostile to Republicans). We understood hostility in the sense of accusing political outgroup members (e.g. Kamala Harris) of incompetence or malevolence. The target difference in perceived hostility between the hostile and the nonhostile news approximated a 0.2 difference on a 0–1 scale, *P* < 0.05, whether the stories were true or false.

**Fig. 2.**
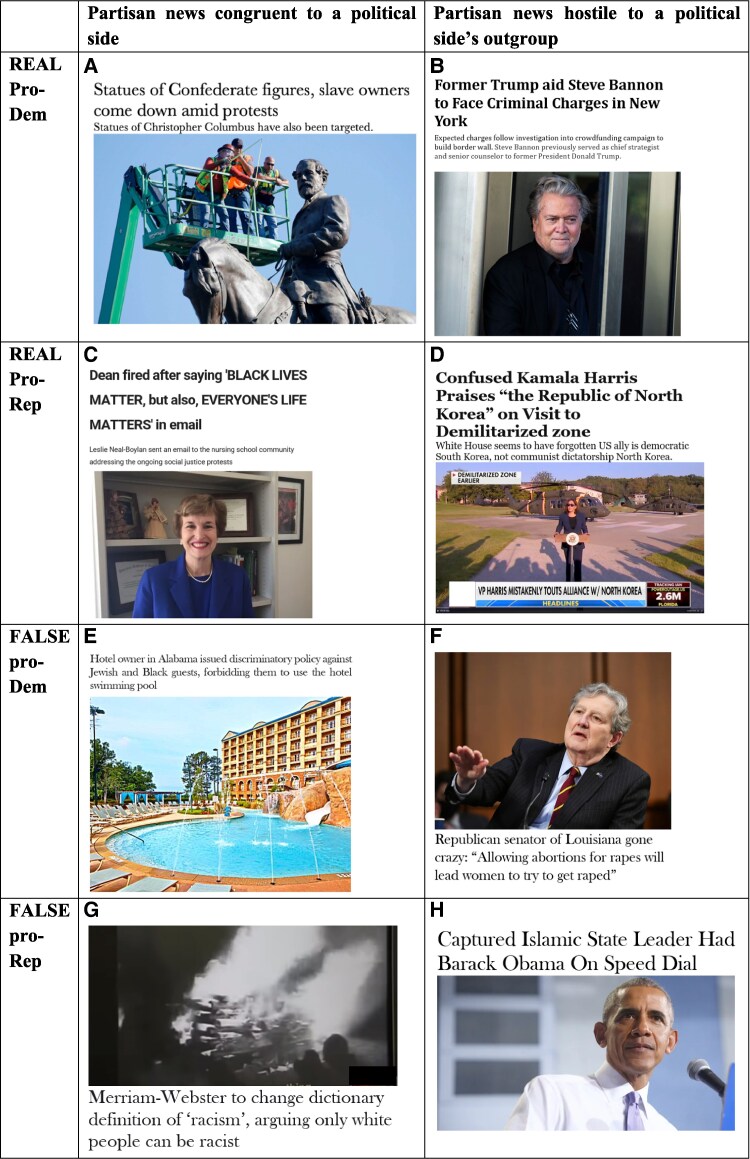
Examples from each one of the eight types of partisan news stories (A-H) that respondents were asked to imagine sharing in a Facebook group in study 2. See “Selection of the news items” section for details on their selection. Two true/real nonpolitical news stories were also included in study 2 as control stimuli. Credit: Getty Images, Associated Press, Wikipedia, ABC News, MSNBC, Fox News, Scientific American, The Sun, The Guardian, The New York Times, NPR, The Wall Street Journal, CNBC, BBC, The Atlantic.

The pretesting phase for study 2 ended when we reached our target of having three partisan news items in each of the following eight categories, as shown in Fig. 2:

See Supplementary Material, C.1 for more details on the selection of the partisan stories and for visualizations of how they varied along the target dimensions. The stimuli database for study 2 also includes two true/real nonpolitical items, covering issues of science and technology and pretested for their political neutrality, used as control items.

### Results

**Fig. 3.**
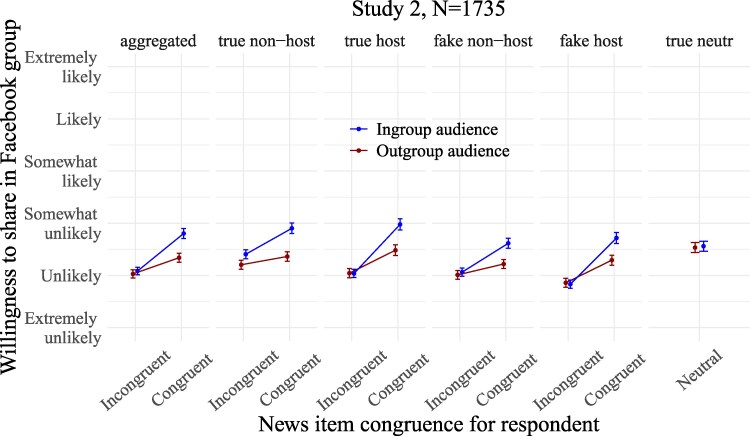
Predicted average intentions to share the news items in the Facebook group in study 2 as a function of their political congruence for the respondent and of the political congeniality of the audience (ingroup vs. outgroup users). From left to right: averaging across all partisan news types, and then breaking down by partisan news types. Whiskers are 95% CIs of the means predicted from the models.

As preregistered, intentions to share were fit using linear regression models with clustered robust standard errors around participant ID to control for the fact that each participant rated 26 items in a random order. Coefficients reported below are from models in which continuous covariates and the intention to share dependent variable were *z*-scored, so we report standardized *β*s and 95% CIs between brackets. See Fig. 3 for visualizations of the predicted values from the models (nonstandardized data), and Supplementary Material, F.2, for the same visualizations supplemented with density plots for the raw data. Average scores of intentions to share as a function of news congruence, audience composition, and news type are found in Supplementary Material, F.4–F.6.

#### Audience effects on intentions to share

Consistent with prior work (12, 14), respondents shared more news stories politically congruent to their partisanship than incongruent news (*β* = 0.32 [0.29, 0.35], *P* < 0.001), supporting H1.

As expected, the sharing preference for congruent news was greater in the presence of an ingroup than an outgroup audience (congruence×audience: *β* = 0.41 [0.31, 0.51], *P* < 0.001). Restricted analyses on pooled congruent partisan news suggested that this positive interaction was mostly driven by the presence of an ingroup audience increasing congruent news sharing compared with when dealing with an outgroup audience (*β* = 0.29 [0.21, 0.37], *P* < 0.001), supporting H2. Breaking down by news types, imagining dealing with an ingroup (vs. an outgroup) audience increased willingness to share all congruent partisan news, whether the news items were true and nonhostile (*β* = 0.34 [0.25, 0.42], *P* < 0.001), true and hostile (*β* = 0.31 [0.22, 0.40], *P* < 0.001), false and nonhostile (*β* = 0.25 [0.17, 0.33], *P* < 0.001), or false and hostile (*β* = 0.27 [0.18, 0.36], *P* < 0.001).

We said in the preregistration of study 2 that in the event of nonsignificant differences in intentions to share ideologically incongruent stories (H3a), we would run indifference tests. For simplicity purposes, we slightly deviate from this plan by simply reporting difference tests even when they are null (thereby providing absence of evidence of a statistical difference). Restricted analyses showed that the presence of an ingroup vs. outgroup audience had no statistically significant effect on sharing of incongruent partisan news pooled together (*β* = 0.03 [−0.03, 0.10], *P* = 0.31), as anticipated by H3a. Considering each news type separately, this absence of audience effect on sharing of incongruent news was observed on most news types, except the true nonhostile news, for which sharing was greater in the presence of an ingroup than an outgroup audience (true nonhostile: *β* = 0.13 [0.05, 0.20], *P* = 0.0013; true hostile: *β* = 0.00 [−0.08, 0.07], *P* = 0.92; false nonhostile: *β* = 0.03 [−0.04, 0.10], *P* = 0.35; false hostile: *β* = −0.02 [−0.09, 0.06], *P* = 0.66).

The manipulation of the audience's political orientation had no detectable effect on intentions to share true neutral news, unrelated to politics (*β* = 0.03 [−0.11, 0.16], *P* = 0.7). This suggests that the social motivations for sharing news, which we anticipated to be more activated by ingroup audiences, mostly operate with respect to content that can affect the sharer's moral reputation or the followers’ perceptions of political issues.

### Discussion

Study 2 experimentally demonstrates that intentions to share partisan news are influenced by the perceived political orientation of one's audience. Imagining surfing on a Facebook group frequented by political ingroup (vs. outgroup) members increased respondents’ willingness to share news congruent to their partisanship, whether true and false, hostile, or not. In contrast, sharing of incongruent partisan news, and of true neutral news, tended not to be affected by the manipulation. Relational or social motivations thus seem to mostly shape intentions to share stories that are politically relevant, and which simultaneously fit the users’ and the audiences’ political identities or convictions.

## Study 3: investigating the motivations driving partisan sharing

Study 3 replicated study 2, complementing it with a politically mixed audience condition. Most importantly, it tested a range of intrinsic, epistemic, and social motivations potentially driving partisan sharing, and in particular, the responsiveness of sharing intentions to the political orientation of the audience. Specifically, study 3 tested whether, in addition to perceived truth and congruence to moral convictions, being liked by one's ingroup audience, mobilizing it to support valued causes, and getting the news fact checked, were driving intentions to share.

### Method

#### Preregistered hypotheses

All the materials, hypotheses, sample size and analyses of study 3 were preregistered at: https://osf.io/cdtym/?view_only=93f206347e704e239b4c280b0f9d68be.

Like in study 2, we expected (H1) stories ideologically congruent to respondents to be generally more shared than incongruent news, regardless of audience composition. We also made the preregistered prediction (H2) that sharing of congruent news would be higher in the ingroup audience than in the outgroup audience condition. We left open which effect the audience manipulation might have on intentions to share incongruent news (H3a envisaging no effect, H3b making room for an increase in the outgroup audience condition).

Moreover, we made preregistered hypotheses about the influence of the audience manipulation on the social motivations for sharing and refraining from sharing the congruent news, which we thought would reflect the role of relational motivations in partisan sharing. First, we predicted respondents to report *reputational* concerns to be liked by the audience as a motivation for sharing congruent news more often in the ingroup audience than in the outgroup audience condition (H4a) (we also say “pleasing” the audience interchangeably). Accordingly, we expected a concern to avoid being liked less by the audience as a motivation *not* to share congruent news to be more pronounced in the outgroup than in the ingroup condition (H4b). Additionally, we predicted respondents to report *mobilization-related* motivations for sharing congruent news in the ingroup audience more often than in the outgroup audience condition (H5a). And accordingly, we hypothesized participants to report mobilization-related motivations *not* to share congruent news in the outgroup audience more often than in the ingroup audience condition (H5b).

We made no hypotheses about the influence of the audience manipulation on motivations for sharing the stories based on more intrinsic factors (i.e. perceived truth and affirmation of moral convictions).

#### Participants

Like in study 2, we powered study 3 based on a hypothetical effect of ingroup vs. outgroup audience on sharing intentions of congruent news. This was the effect we deemed most important to elicit (even if the design now had an additional mixed audience condition; see below). A power analysis ran in Gpower suggested that 1,500 participants were required to detect a small effect of ingroup vs. outgroup audience of *d* = 0.1 at 80% power (alpha level = 0.05). Although we did not formally preregister this, our goal was to arrive at a balanced sample of 50% Democratic and 50% Republican respondents. A total of 1,743 respondents were recruited in total on Prolific and Cloud Research to arrive at the target sample size.^c^ As preregistered, 106 were removed from the data because they failed one of the two pretreatment attention checks (including one intended to suppress bots, see Supplementary Material, B), or because they were neither Democrat nor Republican. Our final dataset thus included 1,637 respondents (*M*_age_ = 43, SD_age_ = 15.7, 50% women), 50% Democrat, and 50% Republican (see Supplementary Material, G.1 for more information). The study was thus even better powered than initially envisaged.

#### Design and procedure

Study 3 was run in June 2023 and took, on average, 21 min to complete. Its design was based on that of study 2, with allocation to an audience condition following a between-subjects design. Before viewing the stories, participants were asked demographic questions, and about their partisanship. The structure of study 3 changed from study 2 in two main ways to help answer the question of which motivations were driving the effect of the audience composition on the size of partisan sharing. First, a politically mixed audience condition was added as a third experimental condition. It asked participants to imagine sharing the news in a Facebook group whose users were of diverse political orientations—Democrats, Republicans, and Independents—and whose reactions to posts and shares in the group could not be predicted. This condition increased the granularity of our perceived audience manipulated variable, as it was conceptually positioned half way between the ingroup and the outgroup audiences. As a result, the three audience conditions would constitute three degrees of (expected) agreement or tolerance of the audience towards the respondents’ sharing choices. See Supplementary Material, B for the exact wording of the vignettes.

Second, and most importantly, we added seven questions asking respondents to justify their sharing intentions. To accommodate this change, responses to the willingness to share question (“How likely would you be to share this news story in this Democrat/politically mixed/Republican Facebook group?”) took the form of two alternatives: [0] “I would never share this news story in this Democrat/politically mixed/Republican Facebook group,” vs. [1] “I would potentially share this news story in this Democrat/politically mixed/Republican Facebook group.”

We dichotomized the intention to share question to allow two batteries of justificatory questions to appear on the survey page depending on their response to the intention to share question (see Table 2). If respondents said they would potentially share a news item with a given audience (Democrat, mixed, or Republican), they saw seven questions asking them why, in a random order. We made preregistered hypotheses about reputation-related and mobilization-related motivations for sharing only. Reputation-related motivations for sharing were quantified based on the average of ratings to two questions: “Because this audience would have more sympathy for me if I shared it”; “Because this audience would praise me if I shared it.” Mobilization-related motivations were quantified by averaging responses to “Because this story could be useful for mobilizing this audience against political groups I don't like” and “Because this story could persuade this audience to support political causes I identify with.” Among the seven possible motivations for sharing given on each survey page, we also offered the possibility to report more intrinsic and epistemic motivations: perceived accuracy, congruence to the respondents’ moral convictions, and need for a fact-check by the audience (Table 3).

**Table 3.** Motivations for sharing and refraining from sharing the news tested in study 3.

	[0] “I would never share this news story in this Democrat/politically mixed/Republican Facebook group”	[1] “I would potentially share this news story in this Democrat/politically mixed/Republican Facebook group”
Perceived truth	“Because I think the news story is false”	“Because I think the news story is true”
Congruence to moral conviction	“Because sharing this news story would go against my core moral beliefs or convictions”	“Because sharing this news story would affirm my core moral beliefs or convictions”
Reputation-related / Being like more (index)	“Because this audience would have less sympathy for me if I shared it” “Because this audience might insult me if I shared it"	“Because this audience would have more sympathy for me if I shared it”; “Because this audience would praise me if I shared it”
Mobilization-related (index)	“Because this story would not be useful for mobilizing this audience against political groups” “Because this story could not persuade this audience to support political causes I identify with”	“Because this story could be useful for mobilizing this audience against political groups I don't like” “Because this story could persuade this audience to support political causes I identify with”
Fact-checking	“Because I don't think this audience could help me fact-check this claim”	“Because this audience could help me fact-check this claim”

Either batch of seven justificatory questions was displayed on the survey page depending on whether the respondent said they would be willing to share the news story [1] or not [0].

All responses to the motivations for sharing questions were recorded on a five-point scale ranging from [1], “strongly disagree” to [3] “neither agree nor disagree” (midpoint) to [5] “strongly agree.” If, in contrast, a respondent reported she would *never* share a given news story, she was presented with seven questions tapping the same potential motivations described above but formulated in the negative (Table 3).

As regards the news stories employed, only two items in each one of the eight categories of partisan news used in study 2 were included in study 3 to shorten the survey, given the additional questions. We selected the two items per category of partisan news which had been subject to the greatest ingroup vs. outgroup audience effects on sharing when being congruent for participants in study 2 (see Supplementary Material, C.6, for visualizations of how the selection was done, and the end of the R analysis script of study 2). No neutral stories were included, so study 3 only contained 16 items, all partisan in tone. Each respondent viewed all 16 partisan news stories. Participants were prevented from completing study 3 if they had taken study 2.

To check their validity, principal component analyses (PCAs) were conducted on the five motivations for sharing different news types (true congruent and incongruent news, false congruent and incongruent news), in the context of two distinct audiences (ingroup and outgroup), by running the PCAs on subsets of the data. With proportions of explained variances being relatively equal across the five motivations and in the 15–20% range, PCAs confirm that the five main motivations measured in study 3 capture distinct psychological constructs. See Supplementary Material, H.4 for detailed results.

### Results

As preregistered, intentions to share and the seven motivations for sharing were fit using linear regression with clustered robust standard errors around participant ID to account for the fact that each participant rated several items. The dichotomous willingness to share dependent variable was kept as is, but ratings of the seven motivations for sharing and not sharing questions were *z*-scored.

#### Audience effects on intentions to share

**Fig. 4.**
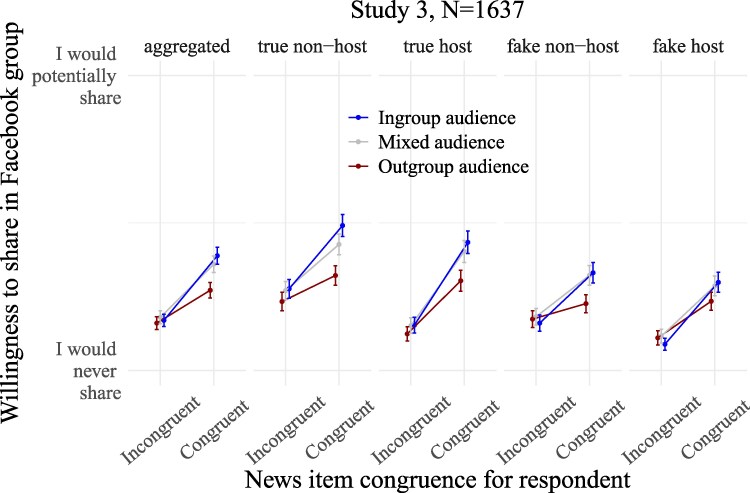
Predicted average intentions to share the news items in the Facebook group in study 3 as a function of their ideological congruence for the respondent and of the political congeniality of the audience (ingroup vs. mixed vs. outgroup audience). From left to right: averaging across all partisan news types, and then breaking down by partisan news types. No neutral items were present in study 3. Whiskers are 95% CIs of the means predicted from the models.

Figure 4 plots the effects of the audience manipulation on intentions to share, displaying predicted values from the models (nonstandardized data). As preregistered, all partisan news are pooled together in the analyses. See Supplementary Material, G.2 for the same visualizations supplemented with density plots of the raw data. All average scores and analyses of intentions to share are found in Supplementary Material, G.3–G.5.

In line with study 2 and prior research, respondents reported higher intentions to share news stories politically congruent to their partisanship than incongruent ones in study 3 (*b* = 0.17, [0.16, 0.18], *P* < 0.001), supporting H1.

Replicating study 2, the presence of an ingroup audience amplified the sharing preference for congruent news compared with when the audience was the outgroup (congruence×audience: *b* = 0.11 [0.07, 0.14], *P* < 0.001). Analyses restricted to congruent news showed that this positive interaction was mainly driven by the ingroup audience increasing intentions to share congruent news compared with the outgroup audience (audience on aggregated congruent news: *b* = 0.12 [0.08, 0.16], *P* < 0.001), confirming H2. Imagining dealing with an ingroup (vs. an outgroup) audience increased sharing of all congruent partisan news, whether true and nonhostile (*b* = 0.17 [0.12, 0.22], *P* < 0.001), true and hostile (*b* = 0.13 [0.08, 0.18], *P* < 0.001), false and nonhostile (b = 0.10 [0.06, 0.15], *P* < 0.001), or false and hostile (*b* = 0.06 [0.02, 0.11], *P* = 0.0059). As indicated by the size of the coefficients, audience effects on congruent news tended to be stronger on the less epistemically problematic partisan news than on more problematic ones.

We said in the preregistration of study 3 that in the event of nonsignificant differences in intentions to share ideologically incongruent stories (H3a), we would use indifference tests. To reduce the complexity of the analyses, we slightly deviate from the preregistration by simply reporting difference tests even when they are null (thereby providing absence of evidence of a statistical difference). Replicating study 2, analyses restricted to incongruent news found that the presence of an ingroup vs. outgroup audience had no significant effect on sharing of incongruent news considered in the aggregate (audience on incongruent news: *b* = 0.01 [−0.02, 0.04], *P* = 0.54), as anticipated by H3a. This lack of a statistically significant audience effect was observed on all incongruent news types (true nonhostile: *b* = 0.04 [−0.00, 0.09], *P* = 0.058; true hostile: *b* = 0.03 [−0.01, 0.07], *P* = 0.11; false nonhostile: *b* = −0.01 [−0.05, 0.03], *P* = 0.49; false hostile: *b* = −0.02 [−0.05, 0.01], *P* = 0.17).

As is visible in Fig. 4, intentions to share congruent news followed a clear pattern: they tended to be lowest when imagining an outgroup audience, comparatively higher when facing a mixed audience, and highest when dealing with an ingroup audience. The mixed audience (composed of Democrats, Republicans, and Independents) operates as a control condition—neither particularly friendly nor unfriendly—and assists in interpreting the sharing trends. The fact that congruent news sharing is tendentially lower in the outgroup audience than in the mixed audience condition suggests that sharing of congruent news is inhibited by a fear of annoying or being viewed negatively by the outgroup. The fact that congruent news sharing is tendentially higher in the ingroup audience than in the mixed audience condition suggests that intentions to share congruent news are driven by motivations to be liked by and potentially influence the ingroup. In a nutshell, participants are sensitive to varying levels of political congeniality among their audience, and adjust their sharing intentions accordingly to produce social effects.

#### Motivations for sharing and not sharing the news

**Fig. 5.**
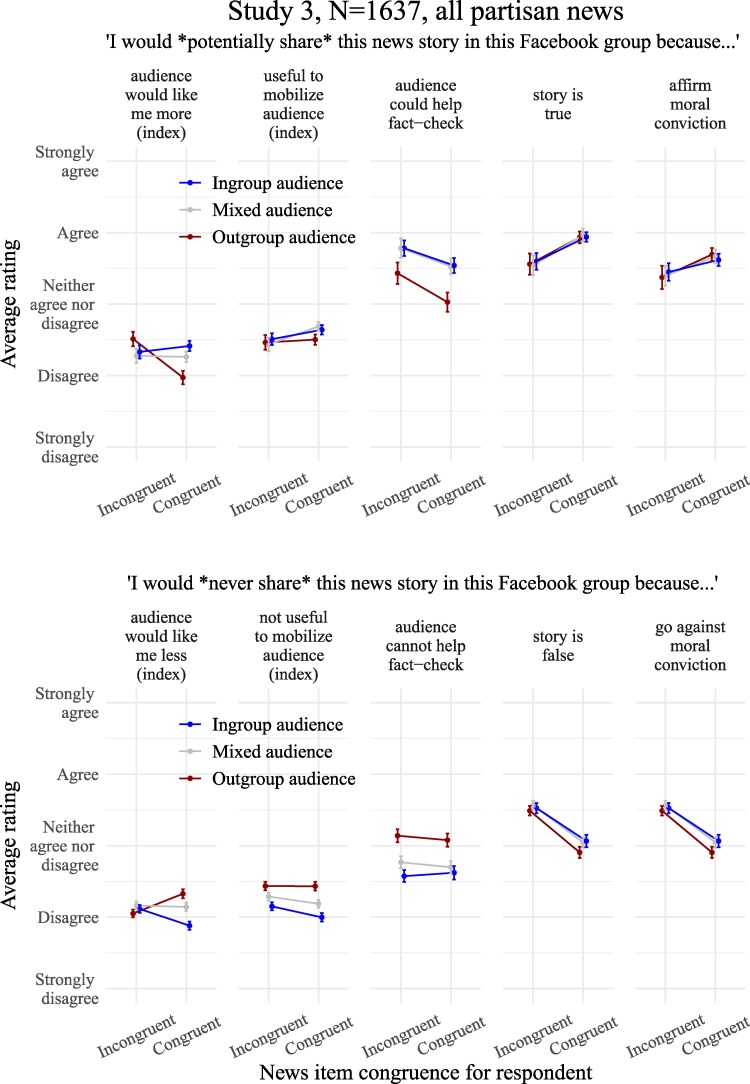
Predicted average motivations for sharing (top) and refrain from sharing (bottom) the aggregated partisan news stories in the Facebook group in study 3 as a function of their ideological congruence for the respondent and of the political composition of the audience. No neutral items were present in study 3. Whiskers are 95% CIs of the means predicted from the models.

In addition to measuring sharing intentions, study 3 examined the variation in self-reported motivations for sharing (and not sharing) the stories in response to the manipulation of the audience composition. As preregistered, all partisan news are pooled together in the analyses. Figure 5 plots the effects of the audience manipulation on the five motivations for sharing tested, showing predicted values from the models (nonstandardized data). See Supplementary Material, H.6 and H.7 and H.10 and H.11 for the same visualizations supplemented with density plots of the raw data. All average scores and analyses of the motivations for sharing (or not sharing) are found in Supplementary Material, H.5 and H.9.

#### Relative importance of the motivations for sharing and not sharing the news

We had no particular expectations with respect to the relative importance of each one of the five main motivations for sharing. A look at Fig. 5 suggests that the main considerations driving motivations for sharing congruent and incongruent partisan news (considered in the aggregate) are perceptions of the stories as true, as promoting an important moral conviction, and as needing to be fact checked. Accordingly, refusal to share is most often motivated by perceptions of the stories as false, or as undermining moral convictions, with average ratings falling between the “Agree” and “Neither agree nor disagree” regions. In contrast, the two social motivations for sharing the news we tested—being liked more by audiences and mobilizing them—feature less prominently in respondents’ motivations, with ratings falling between the “Neither agree nor disagree” and “Disagree” area. For instance, we obtained *b*s = 1.21–1.53, *P* < 0.001, when comparing mean ratings of motivations for sharing all news types based on “audience could help fact-check,” “story is true,” and “affirm moral conviction,” to the social motivation the “audience would like me more,” defined as baseline. See Supplementary Material, H.1–H.3 for tables displaying average scores and associated tests.

#### Audience effects on the motivations for sharing and not sharing ideologically congruent stories

Our first preregistered prediction was that reputation-related motivations for sharing congruent news would be reported more often when the audience is the ingroup rather than the outgroup. Supporting our expectation, participants reported higher motivations for sharing congruent stories in order to be liked by the audience when the audience was the political ingroup (vs. the outgroup) (*b* = 0.44 [0.32, 0.56], *P* < 0.001). Participants also reported higher motivations to refrain from sharing congruent news to avoid being liked less by the audience in the outgroup audience than in the ingroup audience condition (*b* = −0.45 [−0.53, −0.36], *P* < 0.001).

The second preregistered prediction was that mobilization-related motivations for sharing congruent news would be reported more often when the audience is the ingroup rather than the outgroup. In support of this, when the audience was the political ingroup (vs. the outgroup), respondents reported higher willingness to share congruent news to mobilize the audience to support liked causes (*b* = 0.14 [0.04, 0.23], *P* = 0.006). Participants also reported higher motivations to refrain from sharing congruent news because they were not fit for mobilizing the audience in the outgroup than in the ingroup audience condition (*b* = *−*0.44 [−0.52, −0.35], *P* < 0.001).

Another motivation was influenced by the composition of the audience: respondents did report higher willingness to share congruent partisan stories to get them fact checked by the audience when the audience was the ingroup (vs. the outgroup) (*b* = 0.51 [0.34, 0.68], *P* < 0.001). They also reported higher motivations to refrain from sharing congruent news because the audience could not help fact-check them in the outgroup than in the ingroup audience condition (*b* = −0.46 [−0.59, −0.32], *P* < 0.001).

In contrast with the social motivations (being liked more and mobilizing), motivations for sharing the news based on intrinsic and epistemic factors like perceived accuracy and congruence to one's moral convictions were not detectably affected by change in the political composition of the audience. Belief that a congruent news story is true was an equally strong reason to share it in the ingroup than in the outgroup audience condition (*b* = 0.01 [−0.10, 0.11], *P* = 0.924). Belief that a congruent story is false only slightly increased motivations not to share it in the ingroup (vs. outgroup) audience condition (*b* = 0.16 [0.04, 0.28], *P* = 0.008).

Perception of a congruent story as affirming one's moral convictions was an equally strong motivation for sharing it in the ingroup than in the outgroup audience condition (*b* = −0.08 [−0.20, 0.05], *P* = 0.239). Perception of a congruent story as going against one's moral convictions was an equally strong motivation not to share it in the ingroup than the outgroup audience condition (*b* = −0.06 [−0.19, 0.08], *P* = 0.419).

#### Audience effects on the motivations for sharing and not sharing ideologically incongruent news

We had no preregistered expectations as regards the effects of the audience manipulation on the potential motivations for sharing ideologically incongruent news.

Let us begin with the social motivations for sharing. When the audience was ingroup (vs. outgroup), participants reported slightly lower motivations for sharing incongruent news in order to be liked more by the audience (*b* = −0.18 [−0.32, −0.04], *P* = 0.010). The audience being ingroup (vs. outgroup) had no statistically detectable effect on motivations to refrain from sharing incongruent news to avoid being liked less by the audience (*b* = 0.06 [−0.01, 0.14], *P* = 0.109).

The ingroup (vs. outgroup) audience had no effect on motivations for sharing incongruent news to mobilize the audience to support liked causes (*b* = 0.05 [−0.09, 0.18], *P* = 0.488). The audience being ingroup (vs. outgroup) decreased motivations for not sharing incongruent news because they were not fit for mobilizing the audience (*b* = −0.28 [−0.37, −0.20], *P* < 0.001).

Moreover, the ingroup (vs. outgroup) audience increased motivations for sharing incongruent news to get them fact checked by the audience (*b* = 0.35 [0.16, 0.54], *P* < 0.001). The audience being ingroup (vs. outgroup) decreased motivations to refrain from sharing incongruent stories because the audience could not help fact-check them (*b* = −0.57 [−0.69, −0.44], *P* < 0.001).

We now report effects on the intrinsic motivations for sharing incongruent news. The audience being ingroup (vs. outgroup) did not affect motivations for sharing incongruent news because they were seen as true (*b* = 0.04 [−0.15, 0.23], *P* = 0.687). It did not affect motivations for not sharing incongruent news seen as false (*b* = 0.04 [−0.06, 0.14], *P* = 0.44).

The audience being ingroup (vs. outgroup) had no effect on motivations for sharing incongruent news perceived as affirming one's moral convictions (*b* = 0.07 [−0.13, 0.28], *P* = 0.47). It slightly decreased motivations for not sharing incongruent news perceived as going against one's moral convictions (*b* = −0.16 [−0.28, −0.04], *P* = 0.010).

### Discussion

Replicating study 2, study 3 found that imagining navigating a Facebook group frequented by political ingroup (vs. outgroup) members consistently amplified respondents’ partisan sharing, whether the stories were true or false, and hostile or not. Intentions to share incongruent news were not affected, however. As regards the motivations for sharing, believing that a story is true and affirms a moral conviction where crucial reasons to share it, and to refuse to share it when the story clashed with beliefs and convictions. Supporting the idea that partisan sharing is partly driven by social motivations, being liked by ingroup audiences and avoiding clash with outgroup audiences emerged as reasons to share and refrain from sharing congruent news. This supports the notion that partisan sharing of political news is driven by a mix of intrinsic factors and social or relational considerations.

## General discussion

In the United States and other western countries, the diffusion of strongly partisan, hostile, and false news on social media is suspected of fueling ideological and affective polarization between political groups (1–4). Through three well-powered studies, combining online experiments with actual social media data (total *n* = 4,680), we find that tendencies to selectively share more news stories congruent to one's partisanship than incongruent ones is all the more pronounced as users believe they are being read by ingroup members (vs. political opponents). Specifically, sharing of ideologically congruent news tended to increase when the ingroup rather than the outgroup was watching, in all studies. In contrast, sharing of incongruent news, which was consistently lower, tended to slightly decrease (study 1, real news) or be unaffected by the presence of an ingroup (vs. outgroup) audience (studies 2 and 3 for true and false news). Tellingly, Twitter users (study 1) facing a clearly uncongenial audience—despite identifying as clear Democrat and Republican partisans—did not engage in *any* partisan sharing, whether from real or false news domains. Overall, the evidence for audience effects on partisan sharing in the field study 1 is mostly clear for real news (less for false news), and it is compelling on both true and false news in experimental studies 2 and 3.

Contrary to the studies presented above, prior investigations of intentions to communicate political content online have tended to neglect manipulating the political orientation or identity of the audience (12, 14, 19). Consequently, a key condition of partisan communication online has been left unexamined. Since those studies tended to observe marked partisan sharing, participants likely imagined they were dealing with mostly politically friendly audiences—as is often the case on actual social media, and in our own Twitter sample (6, 9–11, 51).

Our results add to the growing literature exploring the social motivations underpinning the diffusion of partisan messages online, such as hostile and false news and posts (21, 24, 61, 62). They corroborate results from recent studies showing that decisions to share messages on digital media depend on a combination of considerations: not only on the messages’ perceived accuracy and congruence with one's moral commitments or identity, but also on the messages’ (perceived) potential for managing relations with like-minded followers (5, 40, 41, 63). Specifically, examination of the reasons for sharing the news in study 3 confirms that individuals care about the accuracy of the content they publish (20), being willing to get it fact checked if necessary, and communicate selectively in ways that promote deep moral convictions (14, 61). But our observation of consistent audience effects (studies 1–3) and analysis of the motivations for sharing (study 3) indicate that accuracy and private moral convictions are not the only determinants. Among the drivers of partisan sharing are also social concerns like enhancing one's reputation. Those can be fulfilled by sharing ideologically congruent news one expects will be liked by political friends, and holding on from sharing claims which may damage one's image or trigger backlash from political foes (40, 41). Additionally, partisan sharing can be motivated by goals of influence and mobilization: by attempts to remind like-minded followers to engage for causes they should care about, and against disliked political actors (26, 34, 37). In line with their social nature, both of these latter motivations for sharing stories increased in the presence of ingroup audiences in study 3.

Several limitations of our studies bear emphasizing. First, all data were collected in the US context, and our audience manipulation in studies 2 and 3 were tailored to its bipolar political spectrum. In multi-party systems and societies divided into more than two major political and cultural coalitions, partisan sharing is likely to come in more numerous forms than mainly a pro-liberal and a pro-conservative variant. Moreover, the degree to which motivations to please or mobilize like-minded audiences translate into political news posting is likely to be constrained in countries enjoying less free speech online than the United States of the Biden presidency. While it is cheap to propagate news criticizing out-party elites or the government in a Western democracy, it can entail major personal costs in authoritarian countries like Turkey, Russia, or Iran (64, 65). In China where the administration massively censors the internet, sharing explicitly critical political content publicly might not even be envisaged (66, 67).

Second, our experimental studies 2 and 3 used vignettes designed to causally elicit audience effects on intentions to share, to compensate the observational nature of study 1. We cannot rule out entirely the possibility of the audience effects being partly strengthened by respondents’ cooperating with the manipulation. Third, it is useful to flag the fact that when audience influences on intentions and decisions to share *false* news were observed in our data (studies 1–3), we cannot know whether participants engaged with the false news because they believed them to be accurate, neglected to assess their plausibility (16), knew them to be false but did not care, or shared them precisely *because* they knew them to be false, to disinform (68). Without measures of perceived credibility of the news shared, it is impossible to answer this question. A default interpretation is that when social media users and experimental participants share fake news, it is because they believe them to be true, or plausible enough. Fourth, our exploration of the motivations for sharing the news in study 3 is based on self-reports, which may suffer from introspection limitations, and they capture those motivations observationally. Future research could benefit from experimentally manipulating features of the news stories—such as, e.g. their potential for mobilizing followers or signaling allegiances—in order to better identify which reasons for sharing are at play.

Considering the broader picture, our studies align with models of moral and political cognition and communication as structured by multiple motives, some epistemic and others social, sometimes in tension with each other (21, 24, 69, 70). When they reason and talk about abstract topics of society and politics, citizens do not just care about the truth. Their attitudes and speech on these topics are also driven by attempts to be seen in a good light by significant others, build communities with like-minded people, and influence others to take action against disliked groups and social issues (26, 36, 37, 40).^d^ Cross-cultural research shows that communication in small scale societies (75) is similarly structured by social goals of signaling, community making, and policing, in ways that likely reflect evolved adaptations for social life (76–79).

Finally, it is relevant to discuss how problematic partisan sharing and its amplification by ingroup audiences are if they occur on a large scale. To the extent partisans share news and messages that they believe to be true, partisan sharing aligns with Bayesian theory and is subjectively rational (80, 81). But selective sharing at scale can become harmful when the social goals that bolster it work against accuracy and nuance. News and messages that demonize political rivals or exaggerate societal threats are typically better tools for signaling political allegiances and mobilizing others to act than more neutral ones (“Where it bleeds, it leads,” as journalists say) (34, 36, 82, 83). The same for news headlines and articles that suppress nuance and cherry-pick the facts. Given this, misleading, dualistic and hostile claims, and in some circles even fabricated news, can potentially be amplified among people who share the same beliefs and worldviews (3). As a result, circulation of biased accounts among networks who think alike (often referred to as “echo chambers”) can maintain discrepancies in beliefs about what is true, making it more difficult to share a common social reality, e.g. about vaccines’ or GMOs’ safety, the consequences of immigration or the legality of a national election (84). Repeated exposure to caricatural claims on one side can also annoy the other side, thereby reinforcing partisan identities and affective polarization (33, 85).

All this suggests that the information ecologies of our democracies may improve by creating stronger incentives for individuals and platforms to overcome the desire to be praised by and galvanize like-minded followers, to limit the spread of misleading and divise content (33). Recent, encouraging initiatives include rewarding participants to online interactions for demonstrations of intellectual humility (86), promoting social media accounts from less-polarizing politicians (e.g. the “Bipartisan leaderboard” from Duke University's Polarization Lab), and rewarding people for more accurate information sharing (87).

## Supplementary Material

pgaf197_Supplementary_Data

## Data Availability

All data and code are available at https://osf.io/2xdmy/?view_only=b609eafb21e546cdbf113d3d05485cc7.

## Notes

a As argued by (49) in their blog post “Getting Started with Negative Binomial Regression Modeling,” paragraphs 2 and 3: “A Poisson distribution is parameterized by λ, which is both its mean and variance. While convenient to remember, it's not often realistic. A distribution of counts will usually have a variance that's not equal to its mean. When we see this happen with data that we assume (or hope) is Poisson distributed, we say we have under- or overdispersion, depending on if the variance is smaller or larger than the mean. Performing Poisson regression on count data that exhibits this behavior results in a model that doesn't fit well. One approach that addresses this issue is Negative Binomial Regression. The negative binomial distribution, like the Poisson distribution, describes the probabilities of the occurrence of whole numbers greater than or equal to 0. Unlike the Poisson distribution, the variance and the mean are not equivalent. This suggests it might serve as a useful approximation for modeling counts with variability different from its mean.” Retrieved from: https://library.virginia.edu/data/articles/getting-started-with-negative-binomial-regression-modeling.

b Exponentiated coefficients are to be interpreted as multiplicative effects on the original scale of the outcome with coefficients >1 entailing an increase and coefficients <1 a decrease in the outcome variable for a one unit increase in the predictor. Exponentiated coefficients indicate how many times larger (or smaller) the expected count becomes when the predictor increases by one unit, *ceteris paribus*. For instance, an estimate = 1.5 means a one-unit increase in the predictor is associated with a 50% increase in the expected count of shares.

c Prolific has more Democratic than Republican clients in the United States (60). As is often done with such services when using convenience samples, collection of responses was conducted while monitoring the proportion of Democrats and Republicans progressively taking our survey. In the last days of data collection (which took place on 2023 June 4–13), we requested Prolific to collect only Republican respondents, and prevent Democrats from taking it, to arrive at a balanced sample. This enabled us to reach a final total sample of ∼50% Democrats and 50% Republicans. Of course, results from study 3 were not examined before completion of the data collection.

d Those social goals might even operate at the level of private belief formation to an extent, by subtly biasing people towards adopting the worldviews that facilitate acceptance within their social circles, and that favor their self-interests (71–74).

## Preprints

The manuscript was posted on a preprint server prior to publication: https://osf.io/preprints/osf/nmg9h_v3.
