# Supplementary material for: Motivations to connect with like-minded audiences increase partisan sharing on social media
Source: PNAS Nexus. 2025 Jun 14;4(7):pgaf197. doi: 10.1093/pnasnexus/pgaf197 (PMC12210954; doi:10.1093/pnasnexus/pgaf197)
Supplement: pgaf197_Supplementary_Data [file pgaf197_supplementary_data.pdf]

Motivations To Connect With Like-Minded Audiences Increase Partisan Sharing On Social Media

ANTOINE MARIE

MICHAEL BANG PETERSEN

Corresponding author: antoine.marie.sci@gmail.com

Supplementary Materials

Table of Contents

A. Partisanship and personality measures ***4***

Study 1 (Twitter data) **4**

Study 2 (Qualtrics survey) **4**

Studies 2-3 (Qualtrics surveys) **4**

B. Other materials ***6***

Ethical review **6**

Attention checks **7**

Vignettes of Study 3 .... **10**

C. News items used in Studies 2, 3 .... ***11***

C.1. Results of the pretest study for Study 2 .... **11**

Accuracy (all partisan news) 11

Congruence (all partisan news) 11

Hostility 12

C.2 True, non-hostile items **15**

C.3 True, hostile items **20**

C.4 False, non-hostile items **26**

C.5 False, hostile items **31**

C.6 Selection of news items for Study 3 .... **34**

D. Codebook for regression analyses ***36***

E. Analyses of Study 1 (shares, Twitter data) ***37***

E.1 Histograms of distributions of shares **37**

E.2 Distributions of like-minded followers among Democrat and Republican Twitter users **38**

E.3 Groupwise numbers of observations of shares from real and false news domains (congruent and incongruent news) **40**

E.4 Regressions: followers' like-mindedness on shares **42**

E.4.1 Congruent real news (main Allsides' classification) 42

E.4.2 Congruent real news (Bakshy's alternative classification) 48
E.4.3 Incongruent real news (main Allsides' classification) 55
E.4.4 Incongruent real news (Bakshy's alternative classification) 62
E.4.5 Congruent false news (main Allcott et al. 2019's classification) 69
E.4.6 Congruent false news (Grindberg's alternative classification) 76
E.4.7 Incongruent false news (main Allcott et al. 2019's classification) 83
E.4.8 Incongruent false news (Grindberg's alternative classification) 89

E.5 Regressions: associations between political slant of the followers and shares from domains congenial to the followers 101
E.5.1 Shares from real news domains (main Allsides' classification) 101
E.5.2 Shares from false news domains (main Allcott et al. 2019's classification) 102

F. Analyses of Study 2 (intentions to share) 104

F.1 Proportion of Democrats and Republicans as a function of exclusion criteria 104

F.2 Plot: Sharing supplemented with density plots for raw data 104

F.3 Mean values of intentions to share 104

F.4 Regressions: sharing averaging across all partisan news (baseline: Outgroup audience) 106
Congruence & Congruence x audience 106
Audience on congruent news only 107
Audience on incongruent news only 107

F.5 Regressions: sharing true neutral news (baseline: Outgroup audience) 107

F.6 Regressions: sharing breaking down by partisan news types (baseline: Outgroup audience) 108
Audience on congruent news only 108
Audience on incongruent news only 109

G. Analyses of Study 3 (intentions to share) 111

G.1 Proportion of Democrats and Republicans as a function of exclusion criteria 111

G.2 Plot: Sharing supplemented with density plots for raw data 111

G.3 Mean values of intentions to share 112

G.4 Regressions: sharing averaging across all partisan news types (baseline: Outgroup audience) 113
Congruence & Congruence x audience 113
Audience on congruent news only 114
Audience on incongruent news only 114

G.5 Regressions: sharing breaking down by partisan news types (baseline: Outgroup audience) 115
Audience on congruent news only 115
Audience on incongruent news only 116

H. Analyses of Study 3 (motivations for sharing and not sharing) 119

H.1 Mean values of motivations to share 119

H.2 Mean values of motivations not to share 120

H.3 Tests comparing motivations for sharing 121

H.4 Principal component analysis of the main motivations for sharing 122

H.5 Regressions: motivations for sharing, averaging across all partisan news types (baseline: Outgroup audience) 126

H.6 Plot: motivations for sharing supplemented with density plots for raw data 131

H.7 Plots: motivations for sharing, breaking down by partisan news types............................132

H.8 Tests comparing motivations for not sharing...135

H.9 Regressions: motivations for not sharing, averaging across all partisan news types (baseline: Outgroup audience)..135

H.10 Plot: motivations for not sharing supplemented with density plots for raw data..........140

H11. Plots: motivations for not sharing, breaking down by partisan news types...................141

A. Partisanship and personality measures

Study 1 (Twitter data)

Partisanship (question asked in the YouGov survey)

Generally speaking, do you usually think of yourself as a...

1 = Strong Republican
2 = Not very strong Republican
3 =Closer to the Republican party
4 = Independent
5 = Closer to the Democratic party
6 = Not very strong Democrat
7 = Strong Democrat

Study 2 (Qualtrics survey)

Personality

Before being exposed to one of the two Facebook audience vignettes, in Study 2 only, respondents reported their personality and cognitive characteristics by answering batteries of questions, the order of which was randomized (and items within them randomized also). They can be found in the .doc version of the Qualtrics survey on the OSF archive (https://osf.io/2xdmy/) but results were not analysed in this paper. The personality scales we included were Social dominance orientation (8 items), Agreeableness (9 items), Intellectual humility (12 items), Partisan feeling thermometers towards Democrats and Republicans (2 x 6 items), Political knowledge (5 items), Political interest (1 item), and the Cognitive reflection test (4 items). Those questions were seen in a random order before exposure to the experimental treatment so they are very unlikely to have affected intentions to share and the treatment effects.

Studies 2-3 (Qualtrics surveys)

Partisanship

Generally speaking, do you usually think of yourself as a...

	Strong Democrat (1)	Moderate Democrat (2)	Leaning Democrat (3)	Leaning Republican (4)	Moderate Republican (5)	Strong Republican (6)	I refuse identification to either party (99)
	○	○	○	○	○	○	○

B. Other materials

Ethical review

DEPARTMENT OF POLITICAL SCIENCE
AARHUS UNIVERSITY

To Whom It May Concern

Exemption from ethical review

As the Department Head of the Department of Political Science, Aarhus University, I confirm that the study "Motivations To Connect With Like-Minded Audiences Increase Partisan Sharing On Social Media" by Antoine Marie and Michael Bang Petersen has been conducted in full compliance with the law of Denmark, and with the regulations at Aarhus University. Furthermore, as specified in the law of the Danish National Committee on Health Research Ethics (§14.2), surveys "that do not involve human biological material" are exempt from further ethical review (https://researchethics.dk/information-for-researchers/overview-of-mandatory-reporting).

Kind regards

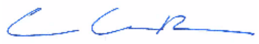

Christoffer Green-Pedersen
Professor, Head of Department

Department of Political Science

Christoffer Green-Pedersen
Professor, Head of Department

Date: 10 June 2025

Direct Tel.: +45 8716 5692
E-mail: cgp@ps.au.dk
Web: au.dk/en/cgp@ps

Sender's CVR no.: 31119103

Department of Political Science
Aarhus University
Bartholins Allé 7
DK-8000 Aarhus C
Denmark

Tel.: +45 8715 0000
Fax: +45 8613 9839
E-mail: ps@au.dk
Web: ps.au.dk/en

AACSB ACCREDITED
ASSOCIATION OF MBAs ACCREDITED
EQUIS ACCREDITED

Attention checks

Study 2

Pre-treatment attention check

videogame

Now imagine you are playing video games with a friend and at some point your friend says: I don't want to play this game anymore! To make sure that you read the instructions, please write the three following words "I pay attention" in the box below. I really dislike this game. Do you agree with your friend?

____

This check was positioned at the beginning of the survey, before allocation to an experimental condition. Respondents who provided text responses that did not contain at least the expression 'pay attention' (where the ideal response was "I pay attention") were filtered out of the final dataset.

Audience manipulation check

manip_check

Just to double check, we've asked you to imagine you are scrolling through posts in a Facebook group that is:
o Democrat (1)
o Republican (2)
o religious (3)
o atheist (4)

This check was positioned immediately after viewing the Facebook audience vignette in the survey. NB: To avoid post-treatment bias, those who provided answers that didn't match the condition they were allocated to were **kept** in the data. In other words, this check was not used to filter out participants.

Study 3

Pre-treatment attention checks

NB: the two following attention checks were presented before random allocation to an audience condition, as part of the demographic questions, to avoid post-treatment bias. They were applied to filter out inattentive respondents or potential bots.

Check_21

[image displaying figure '21']

Please enter the number you see in the image above (use numerical digits):
____

NB: Respondents who did not write '21' were deleted from the data; this was intended to remove potential bots.

Check

Help us keep track of who is paying attention, please select "Somewhat disagree" in the options below.

- ○ Strongly agree (1)
- ○ Agree (2)
- ○ Somewhat agree (3)
- ○ Neither agree nor disagree (4)
- ○ Somewhat disagree (5)
- ○ Disagree (6)
- ○ Strongly disagree (7)

NB: Respondents who did not select '5' were deleted from the data.

Audience manipulation check

manip_check Just to double check, we've asked you to imagine you are navigating on a Facebook group that is:

- ○ Quite Democrat (1)
- ○ Quite Republican (2)
- ○ Quite religious (3)
- ○ Quite atheist (4)
- ○ Politically mixed (5)

This check was positioned immediately after viewing the Facebook audience vignette in the survey. NB: To avoid post-treatment bias, those who provided answers that didn't match the condition they were allocated to were **kept** in the data. In other words, this check was not used to filter out participants.

Vignettes of Study 3

Democrat Facebook group (e.g., ingroup audience for Democrats)	Politically-mixed Facebook group (i.e., ingroup and outgroup members together)	Republican Facebook group (e.g., outgroup audience for Democrats)
Please read very carefully the following text, questions will be asked to you about it.<br><br>Imagine that you are navigating on a Facebook group that is dedicated to one of your hobbies (e.g. a game or sport). Most discussions on the group are, of course, related to the hobby.<br><br>But often discussions about politics also appear, and people frequently share news stories in the group. You know from reading the stories and the comments that the group's members are quite Democrat. They like to point out that Republican politicians and voters are ignorant, that sexism, racism and transphobia are widespread, etc.<br>They stand firm on their political beliefs, which they think are the only right ones. They can be pretty mean to people who don't share their values. On the other hand, they really praise those who agree with them. So if you post and write the kinds of things that they want to hear, you know for sure that they will like you.	*Please read very carefully the following text, questions will be asked to you about it.*<br><br>Imagine that you are navigating on a Facebook group that is dedicated to one of your hobbies (e.g., a game or sport). Most discussions on the group are, of course, related to the hobby.<br><br>But often discussions about politics also appear, and people frequently share news stories in the group. You know from reading the stories and the comments that the group's members are politically very diverse: some are Democrat, some are Republican, and still others are centrists and independents. A wide variety of political viewpoints is thus being expressed every day in this Facebook group. This means that you are free to post and share whatever you want in the group. It also means that you don't know in advance how people might react–some might praise you for sharing something they agree with, but others might be pretty mean if they disagree with it.	*Please read very carefully the following text, questions will be asked to you about it.*<br><br>Imagine that you are navigating on a Facebook group that is dedicated to one of your hobbies (e.g. a game or sport). Most discussions on the group are, of course, related to the hobby.<br><br>But often discussions about politics also appear, and people frequently share news stories in the group. You know from reading the stories and the comments that the group's members are quite Republican. They like to point out that Democrat politicians and voters are ignorant, that immigration, abortion and gun control must be opposed, etc.<br>They stand firm on their political beliefs, which they think are the only right ones. They can be pretty mean to people who don't share their values. On the other hand, they really praise those who agree with them. So if you post and write the kinds of things that they want to hear, you know for sure that they will like you.

C. News items used in Studies 2, 3

Credit: Getty Images, Associated Press, Wikipedia, ABC News, MSNBC, Fox News, Scientific American, The Sun, The Guardian, The New York Times, NPR, The Wall Street Journal, CNBC, BBC, The Atlantic.

C.1. Results of the pretest study for Study 2

Accuracy (all partisan news)

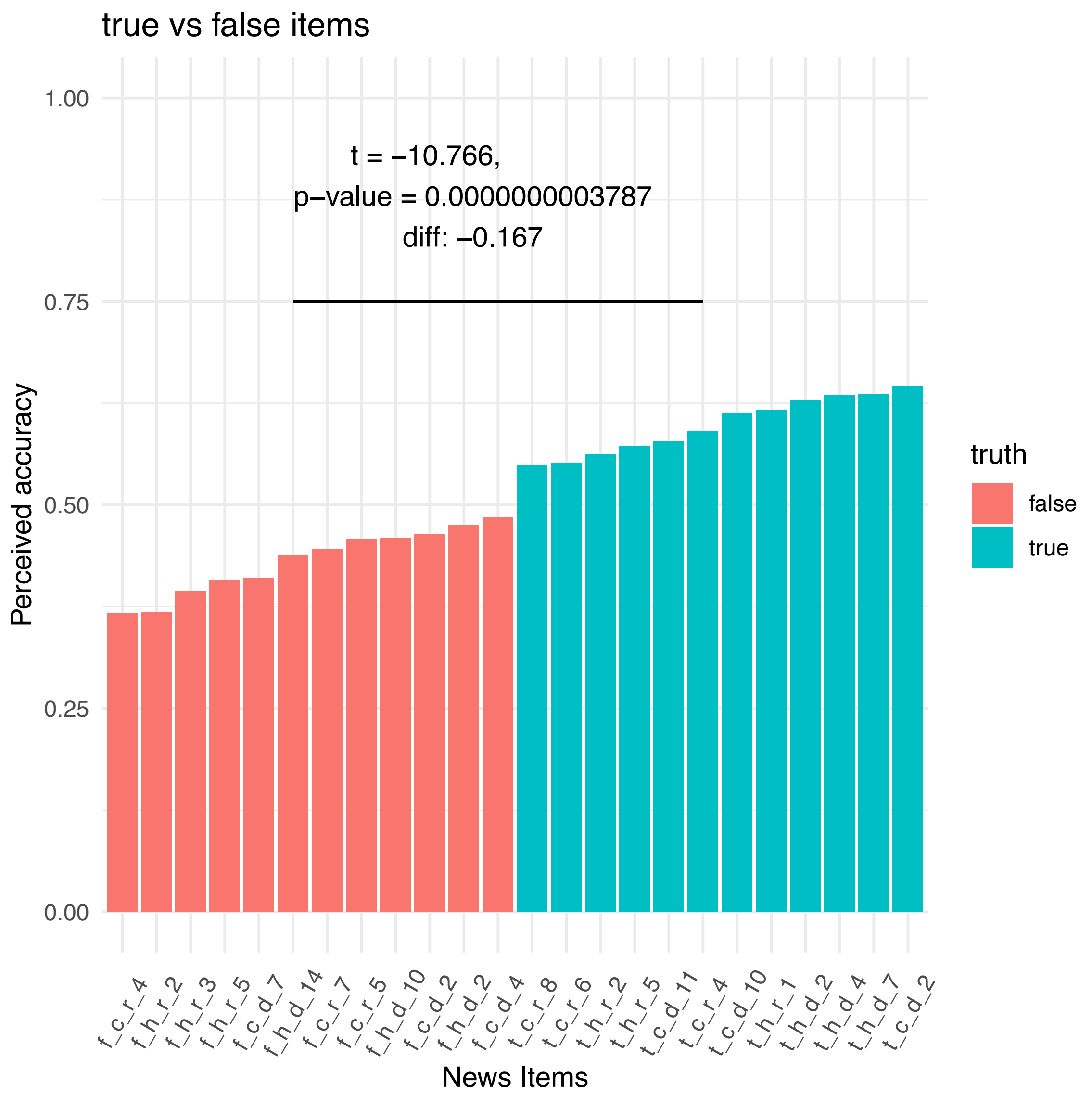

Supplementary figure 1: Results of the pretest study for Studies 2, 3: Accuracy ratings (all partisan news).

Congruence (all partisan news)

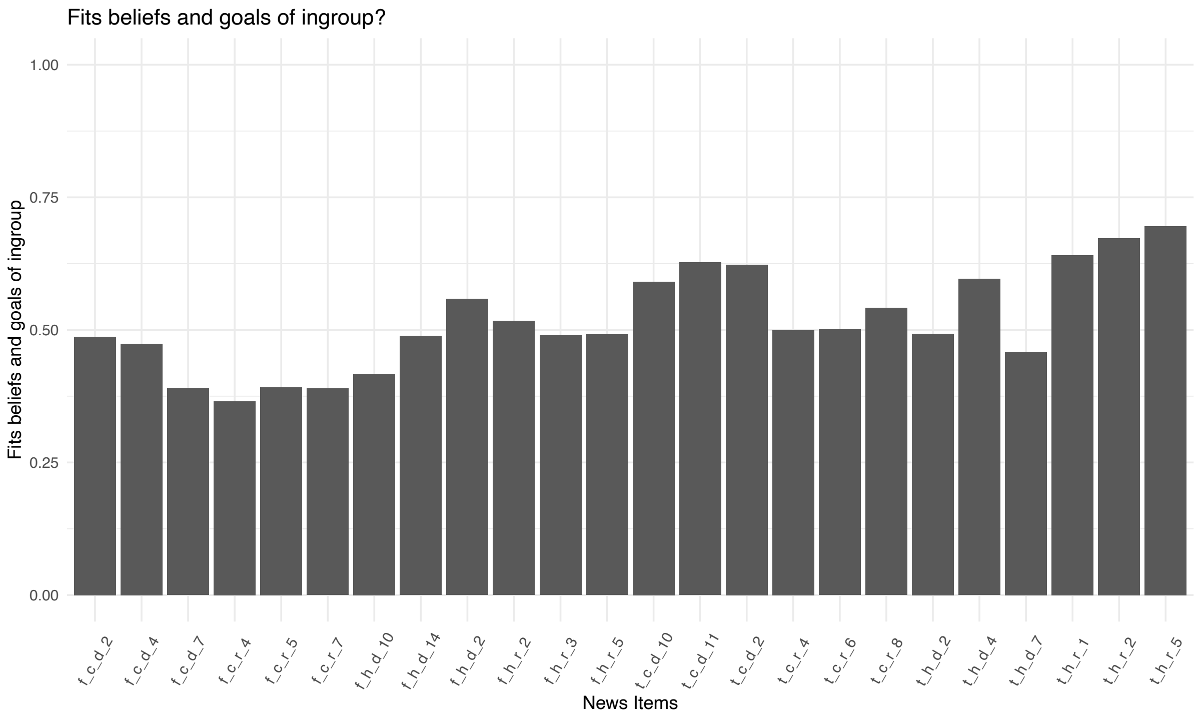

Supplementary figure 2: Results of the pretest study for Studies 2, 3: Ideological congruence ratings (all partisan news).

Hostility

True news congruent to Republicans vs. true news hostile to Democrats

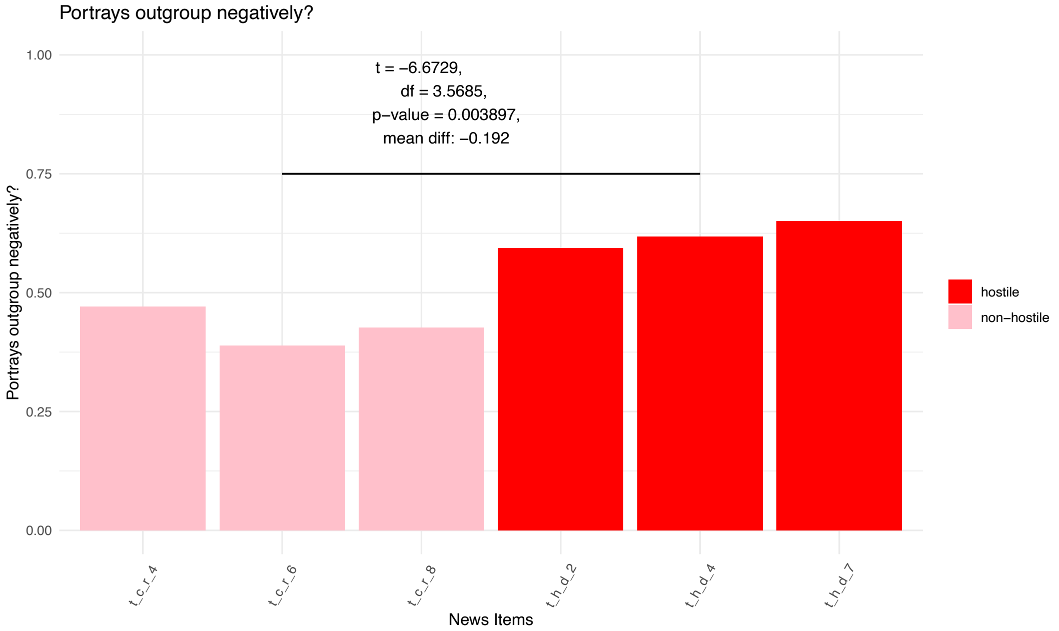

Supplementary figure 3: Results of the pretest study for Studies 2, 3: Hostility ratings of the true news congruent to Republicans vs. true news hostile to Democrats.

True news congruent to Democrats vs. true news hostile to Republicans

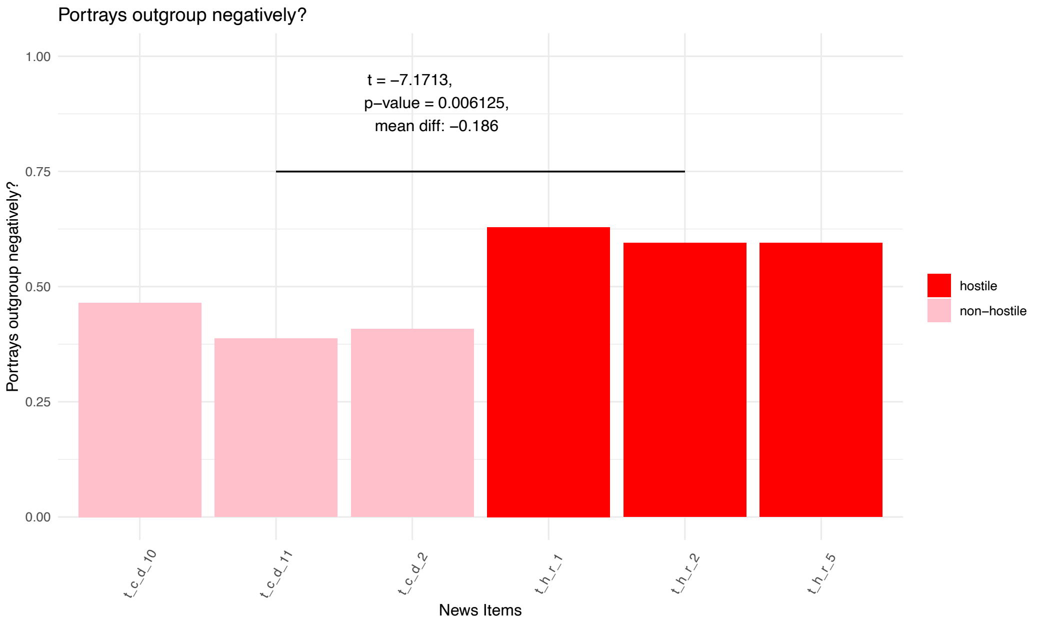

Supplementary figure 4: Results of the pretest study for Studies 2, 3: Hostility ratings of the true news congruent to Democrats vs. true news hostile to Republicans.

False news congruent to Republicans vs. false news hostile to Democrats

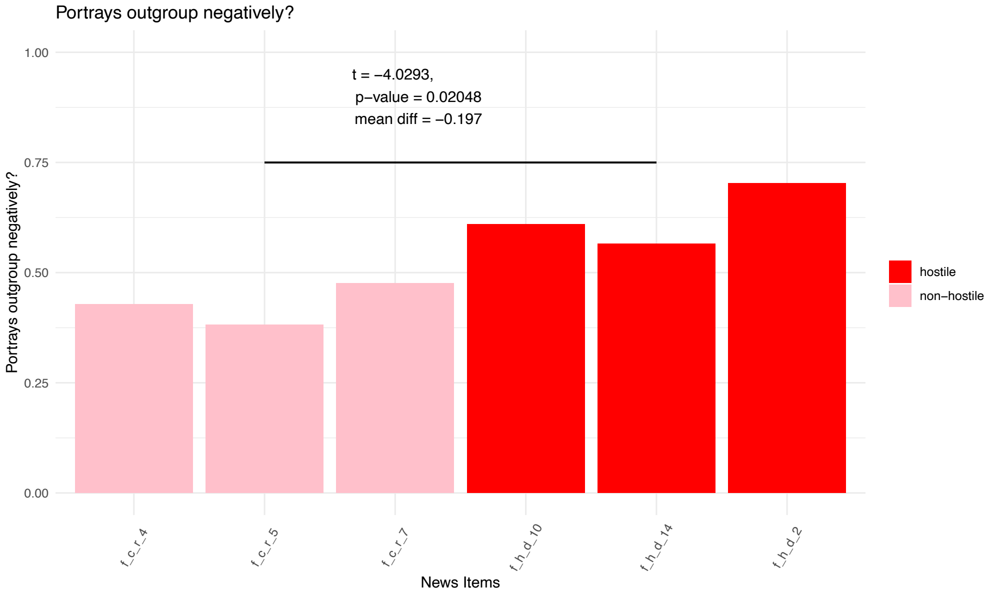

Supplementary figure 5: Results of the pretest study for Studies 2, 3: Hostility ratings of the false news congruent to Republicans vs. false news hostile to Democrats.

False news congruent to Democrats vs. false news hostile to Republicans

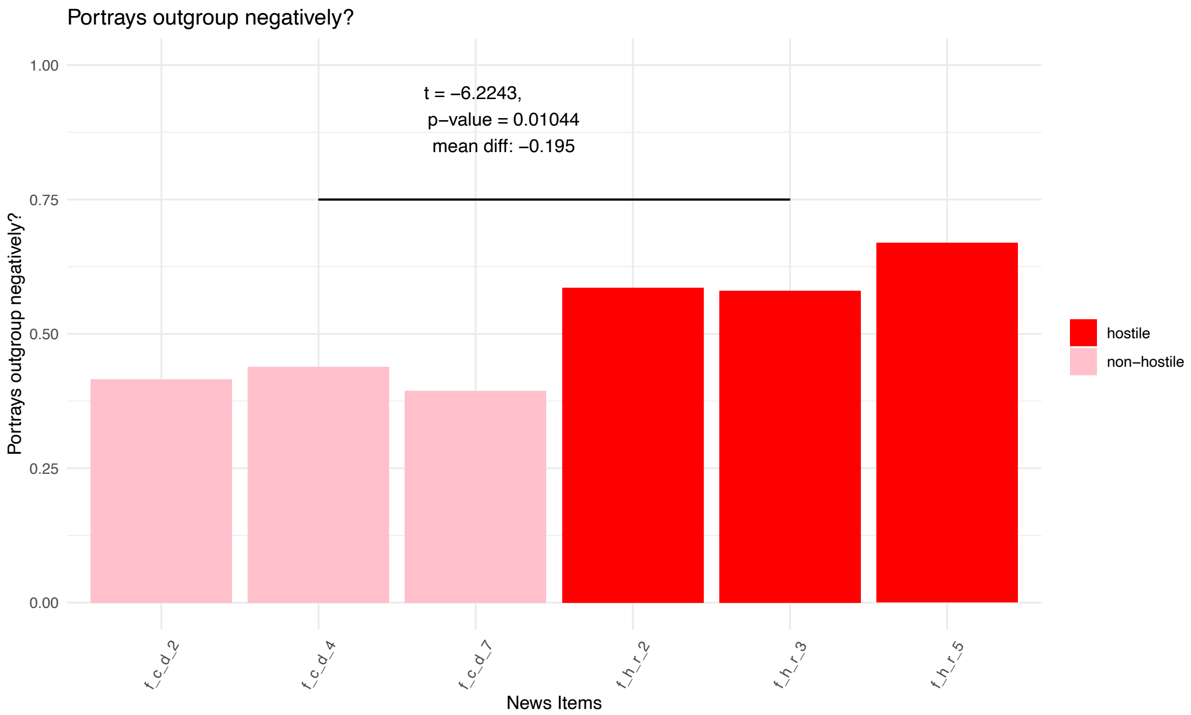

Supplementary figure 6: Results of the pretest study for Studies 2, 3: Hostility ratings of the false news congruent to Democrats vs. false news hostile to Republicans.

C.2 True, non-hostile items

t_c_d_2

Statues of Confederate figures, slave owners come down amid protests

Statues of Christopher Columbus have also been targeted.

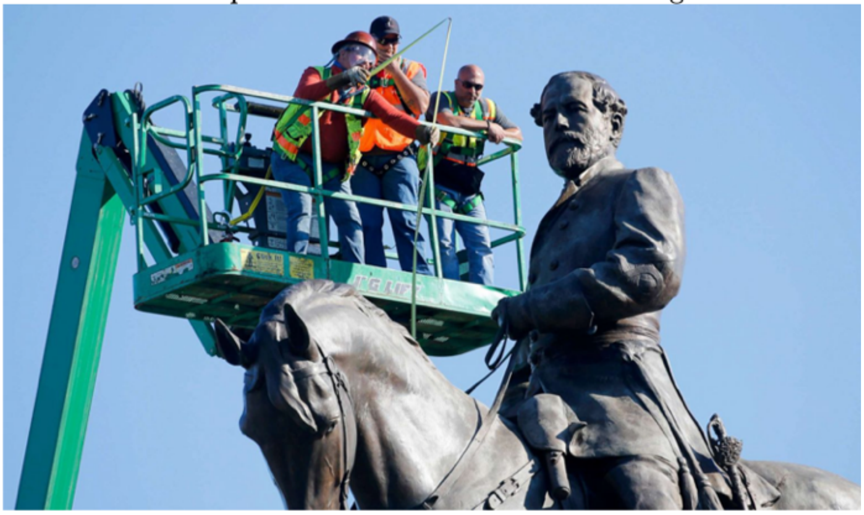

https://abcnews.go.com/US/virginia-indiana-joining-taking-confederate-monuments/story?id=71066712

t_c_d_10

New Book Says Scientists Have Been Underestimating the Pace of Climate Change

A book entitled *Discerning Experts* explains why—and what can be done about it

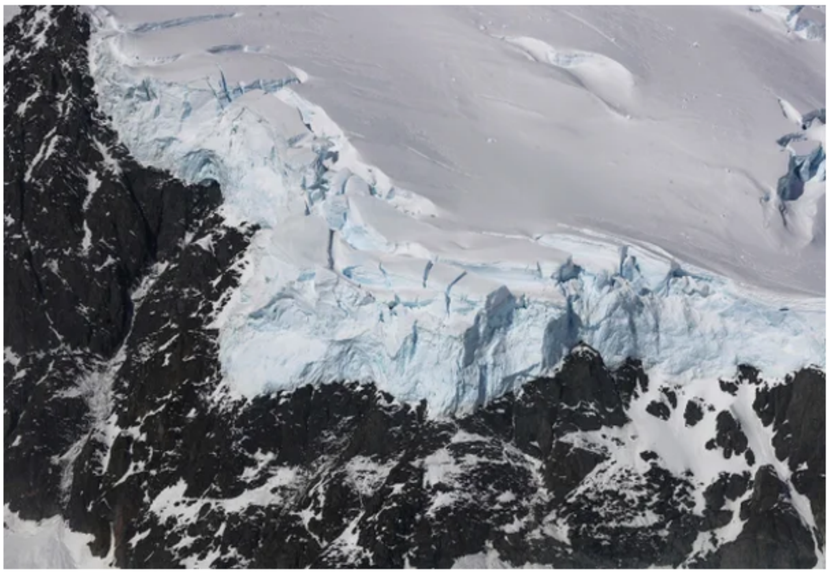

https://blogs.scientificamerican.com/observations/scientists-have-been-underestimating-the-pace-of-climate-change/

t_c_d_11

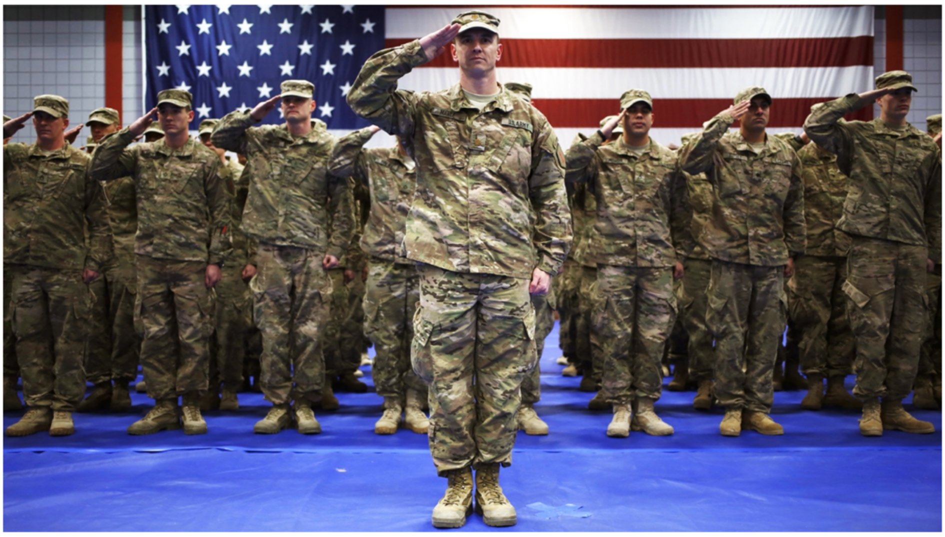

Air Force Academy diversity training tells cadets to use words that 'include all genders'

Air Force Academy also tells cadets to be 'Color Conscious' instead of 'Colorblind', and to "Use words that include all genders: 'Folks' or 'Y'all' instead of 'guys'; 'partner' vs. 'boyfriend or girlfriend.'"

https://www.foxnews.com/politics/air-force-academy-diversity-training-tells-cadets-to-use-words-that-include-all-genders-drop-mom-and-dad

t_c_r_4

Among university professors in social sciences, self-identified Democrats are at least five times as many as Republicans

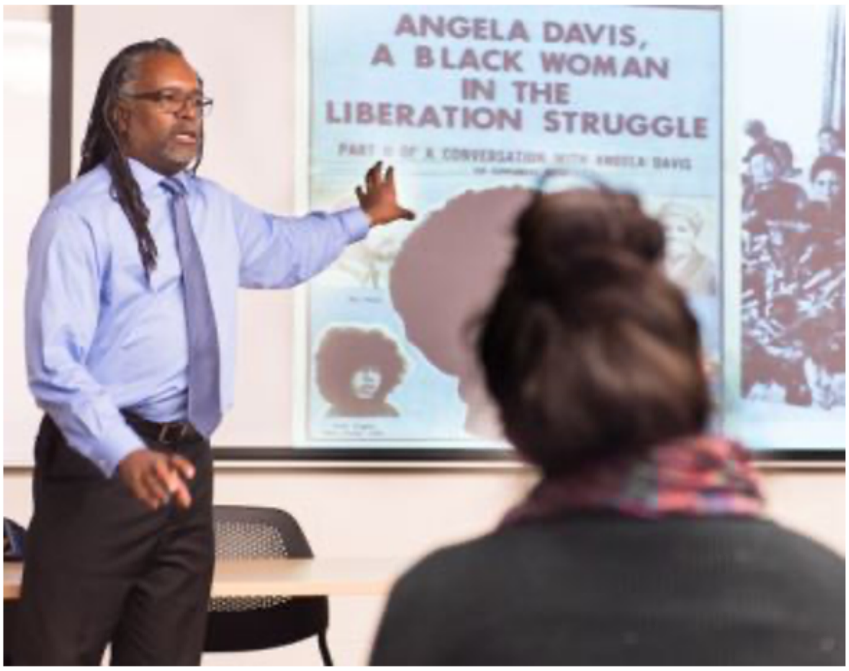

"In the social sciences and humanities, however, there is a stronger imbalance. For instance, recent surveys find that 58–66% of social science professors in the United States identify as liberals, while only 5–8% identify as conservatives, and that self-identified Democrats outnumber Republicans by ratios of at least 8 to 1 (Gross & Simmons 2007; Klein & Stern 2009; Rothman & Lichter 2008). A similar situation is found in the humanities where surveys find that 5277% of humanities professors identify as liberals, while only 4–8% identify as conservatives, and that self-identified Democrats outnumber Republicans by ratios of at least 5:1 (Gross & Simmons 2007; Rothman & Lichter 2008). In psychology, the imbalance is slightly stronger: 84% identify as liberal, whereas only 8% identify as conservative. That is a ratio of 10.5 to 1. In the United States as a whole, the ratio of liberals to conservatives is roughly 1 to 2 (Gallup poll 2010; see Saad 2010)."

Duarte JL, Crawford JT, Stern C, Haidt J, Jussim L, Tetlock PE. Political diversity will improve social psychological science. *Behavioral and Brain Sciences*. 2015;38:e130. doi:10.1017/S0140525X14000430 p. 3

t_c_r_6

Dean fired after saying 'BLACK LIVES MATTER, but also, EVERYONE'S LIFE MATTERS' in email

Leslie Neal-Boylan sent an email to the nursing school community addressing the ongoing social justice protests

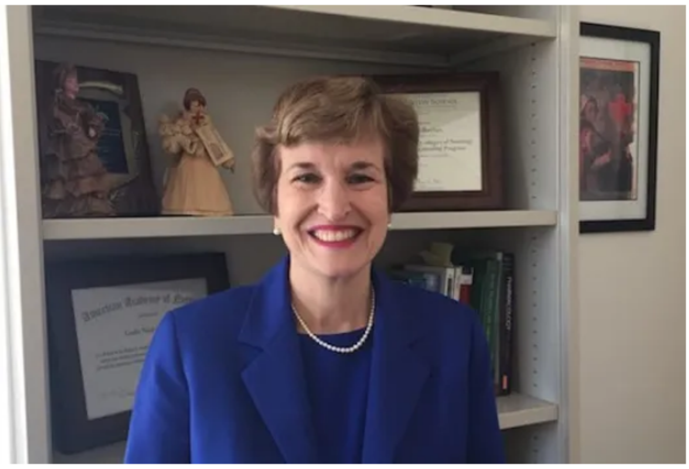

https://www.foxnews.com/us/dean-fired-after-saying-black-lives-matter-but-also-everyones-life-matters-in-email

t_c_r_8

Trump sues CNN for defamation and seeks $475m in punitive damages

Cable news has tried to smear ex-president 'with a series of ever-more scandalous, false, and defamatory labels', says court filing

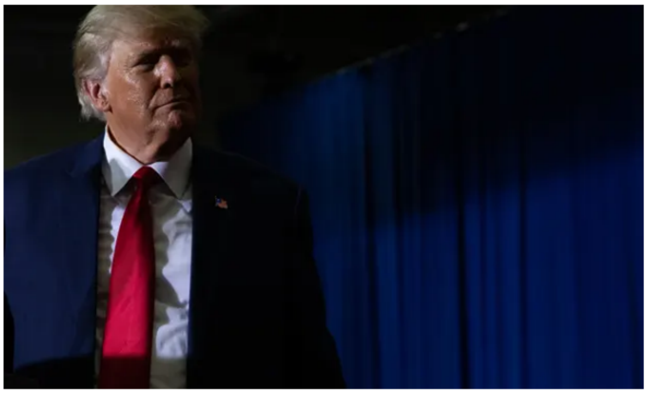

https://www.theguardian.com/us-news/2022/oct/03/trump-sues-cnn-defamation-punitive-damages

C.3 True, hostile items

t_h_d_2

Biden declared the pandemic over. But at least 400 people are dying daily, and unvaxxed Air Force pilots are still grounded.

The president made the remark in an interview that aired on CBS's "60 Minutes" on Sunday night. By Monday, the backlash was in full swing.

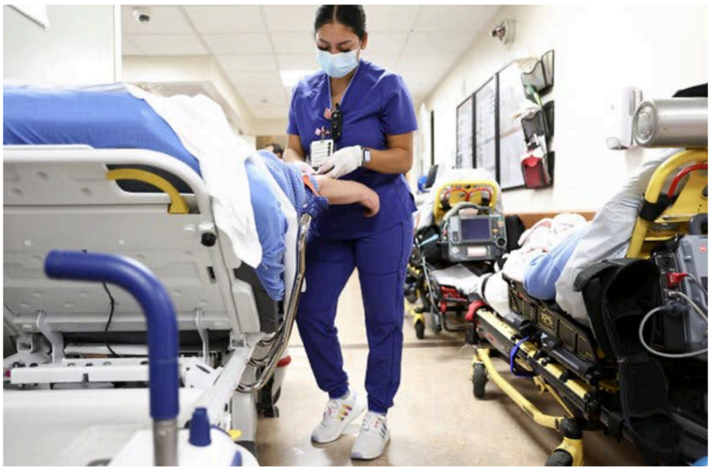

https://www.foxnews.com/politics/biden-declared-pandemic-over-unvaxxed-air-force-pilots-still-grounded
https://www.nytimes.com/2022/09/19/us/politics/biden-covid-pandemic-over.html
https://www.npr.org/sections/health-shots/2022/09/20/1123883468/biden-pandemic-over-complicates-fight

t_h_d_4

Confused Kamala Harris Praises "the Republic of North Korea" on Visit to Demilitarized zone

White House seems to have forgotten US ally is democratic South Korea, not communist dictatorship North Korea.

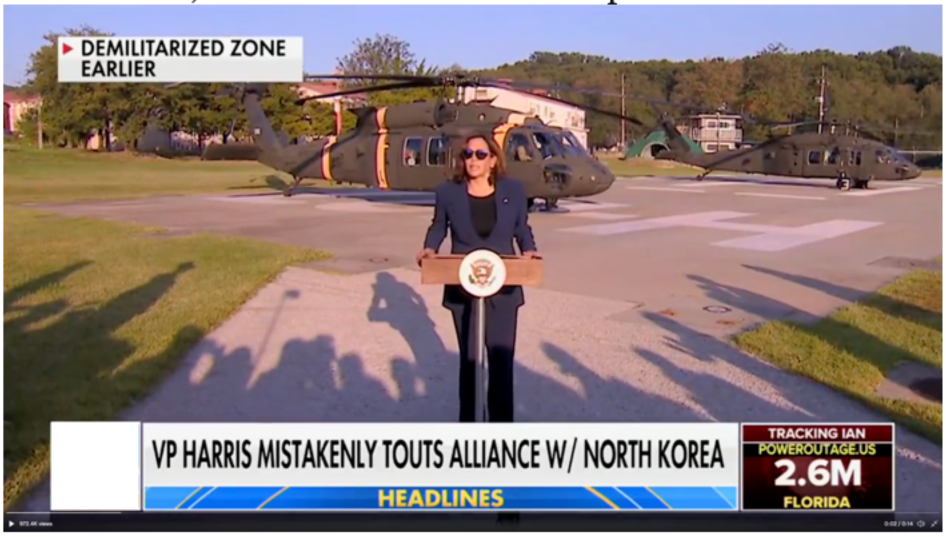

https://twitter.com/RNCResearch/status/1575465283325657095?ref_src=twsrc%5Etfw%7Ctwcamp%5Etweetembed%7Ctwterm%5E1575465283325657095%7Ctwgr%5Eab5454054164517e510809ccbefff827ee76d7ca%7Ctwcon%5Es1_&ref_url=https%3A%2F%2Fwww.breitbart.com%2Fasia%2F2022%2F09%2F29%2Fconfused-kamala-harris-praises-the-republic-of-north-korea-on-visit-to-dmz%2F

t_h_d_7

Sex trafficker Jeffrey Epstein donated to several Democrats throughout 1990s and early 2000s

Jeffrey Epstein, a wealthy financier charged with sex trafficking who killed himself in prison, has been a prolific donor–more than $80,000–to various Democratic campaigns and party committees over the past couple decades

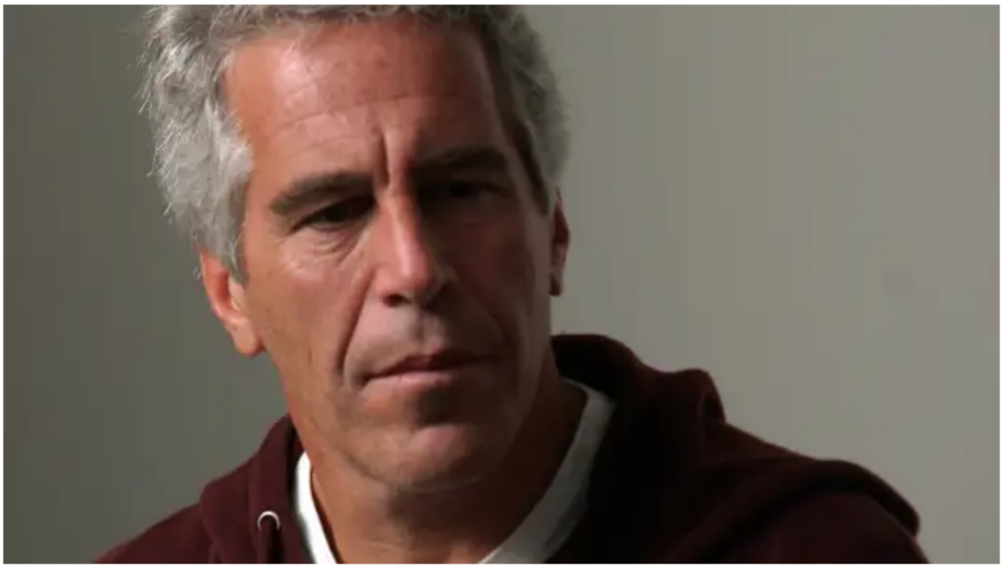

https://abcnews.go.com/Politics/jeffrey-epstein-donated-democrats-1990s-early-2000s/story?id=64255485

t_h_r_1

Former Trump aid Steve Bannon to Face Criminal Charges in New York

Expected charges follow investigation into crowdfunding campaign to build border wall. Steve Bannon previously served as chief strategist and senior counselor to former President Donald Trump.

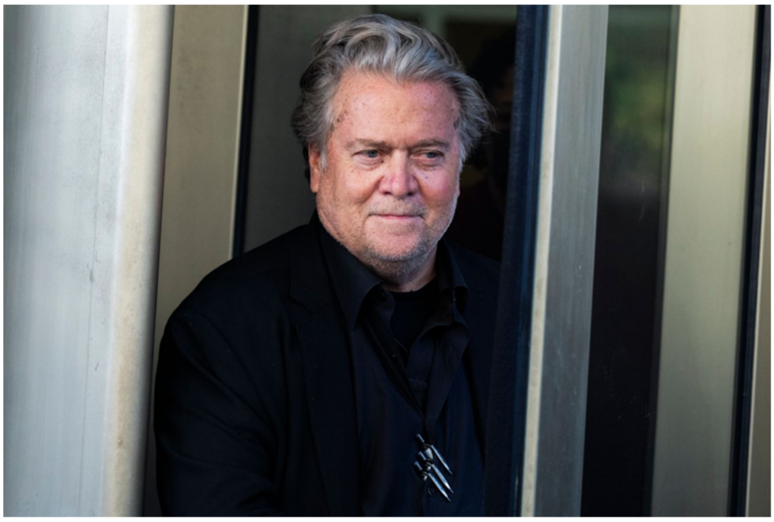

https://www.wsj.com/articles/former-trump-strategist-steve-bannon-to-face-criminal-charges-in-new-york-11662557587?mod=politics_lead_pos2

t_h_r_2

N.Y. Attorney General Accuses Trump of 'Staggering' Fraud in Lawsuit

Attorney General Letitia James of New York filed a sweeping lawsuit on Wednesday that accused Donald J. Trump, his family business and three of his children of lying to lenders and insurers by fraudulently overvaluing his assets by billions of dollars.

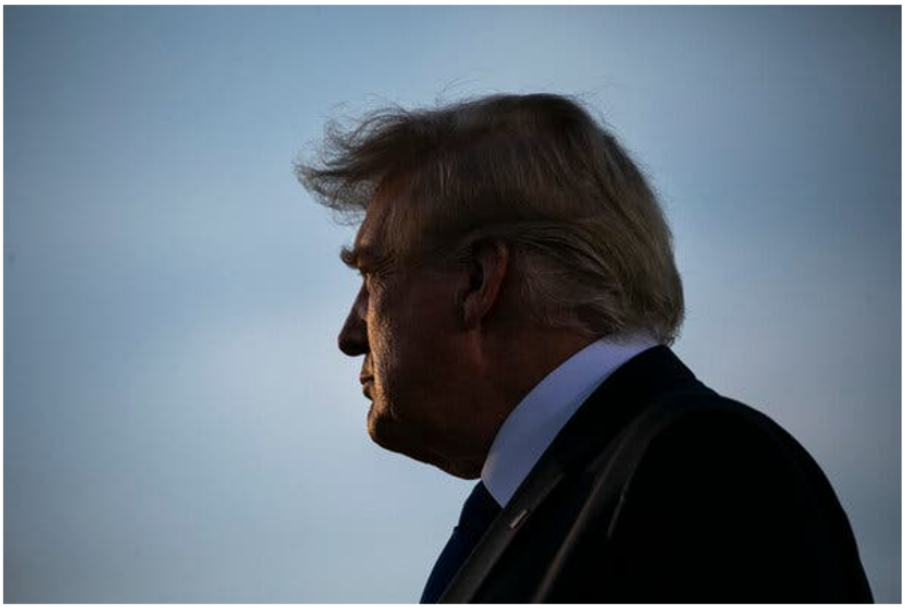

https://www.nytimes.com/2022/09/21/nyregion/trump-fraud-lawsuit-ny.html?searchResultPosition=5

t_h_r_5

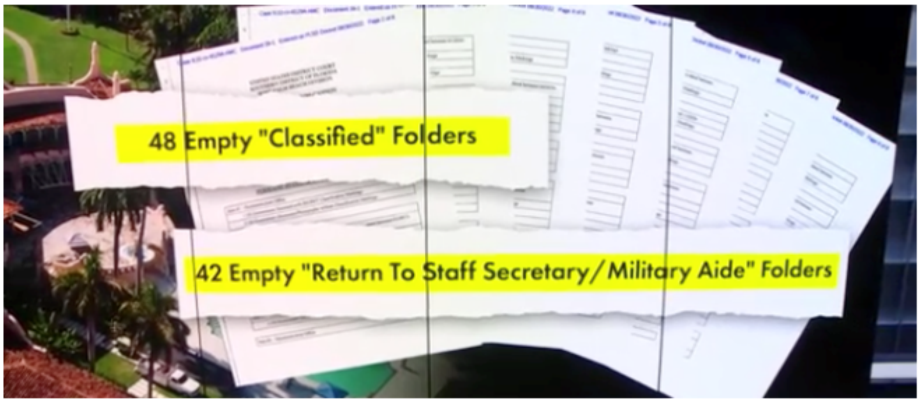

Four dozen empty folders marked 'CLASSIFIED' illegally found in Trump Mar-a-Lago raid, DOJ reveals

FBI agents found four dozen empty document folders marked "CLASSIFIED" during their raid of former President Donald Trump's residence at his Mar-a-Lago club, a court filing shows.

https://www.cnbc.com/2022/09/02/trump-fbi-raid-documents-about-mar-a-lago-search-unsealed.html

C.4 False, non-hostile items

f_c_d_2

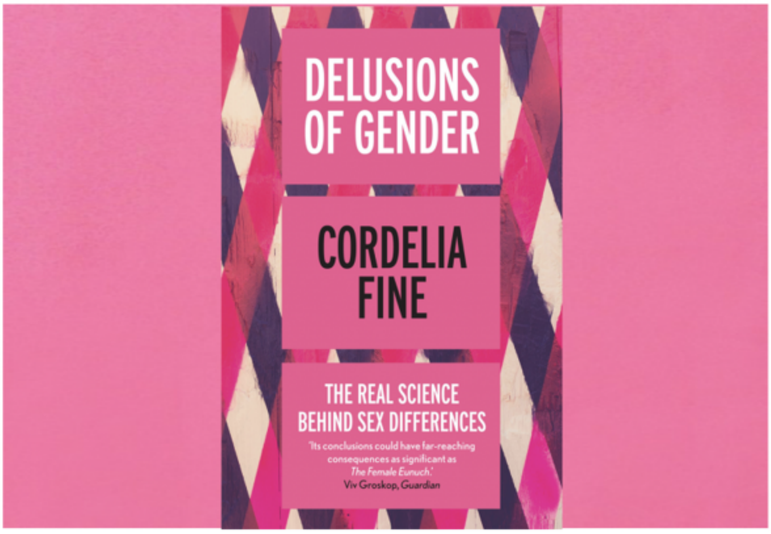

Wide scientific consensus that psychological differences between men and women are entirely explained by education and socialization

f_c_d_4

Black men are fatally shot 1 out of 10 times when arrested by cops

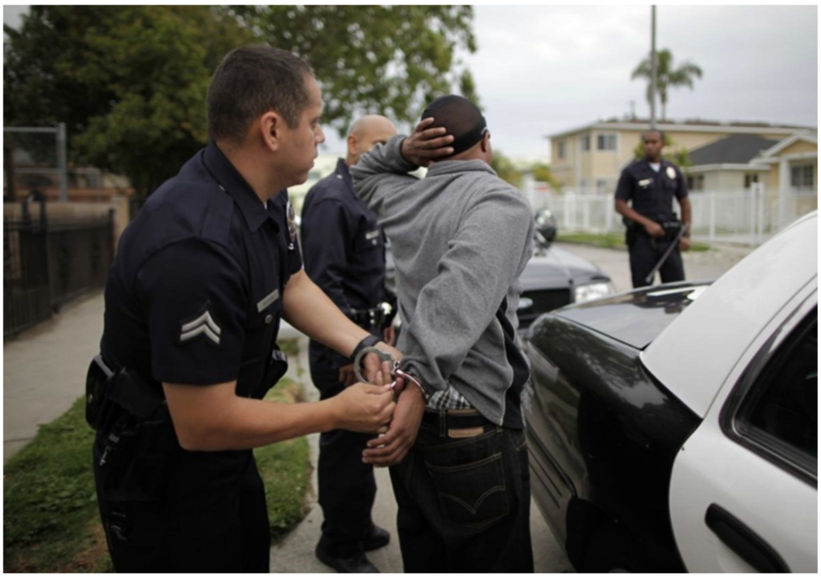

f_c_d_7

Hotel owner in Alabama issued discriminatory policy against Jewish and Black guests, forbidding them to use the hotel swimming pool

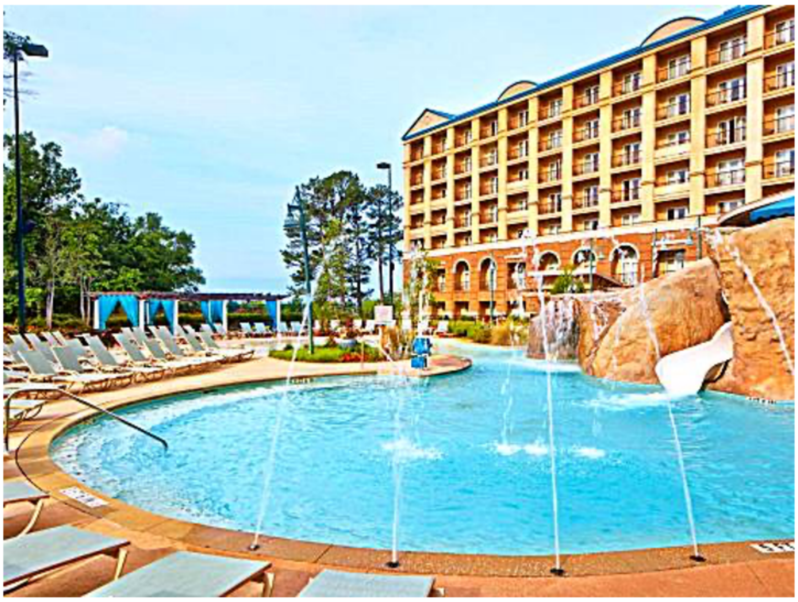

https://www.snopes.com/fact-check/switzerland-hotel-jewish-guests/

f_c_r_4

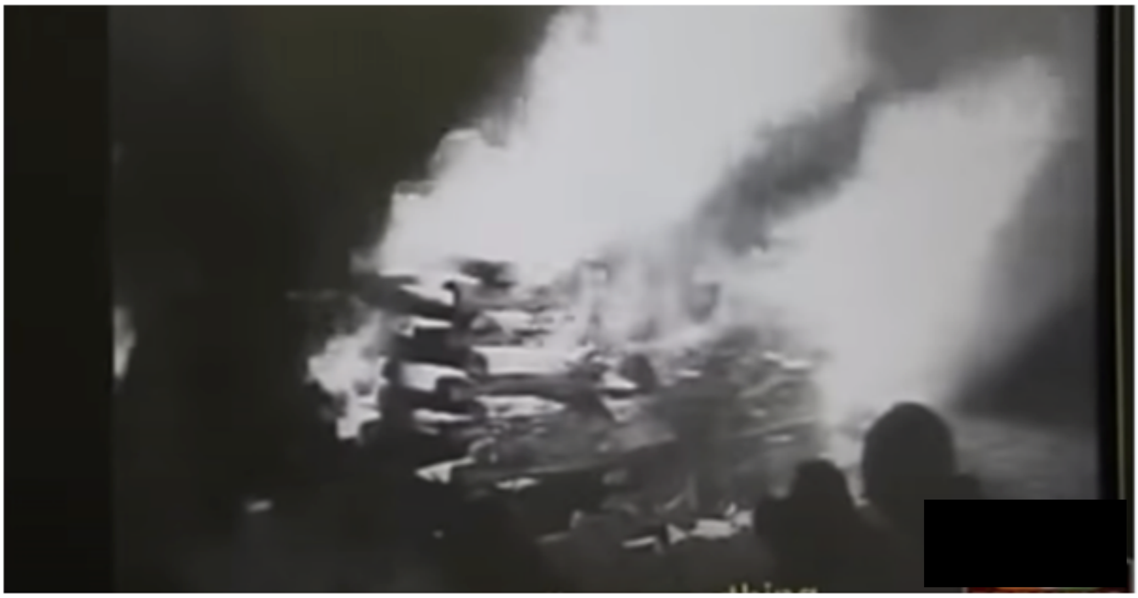

Merriam-Webster to change dictionary definition of ‘racism’, arguing only white people can be racist

https://www.nytimes.com/2020/06/10/us/merriam-webster-racism-definition.html
https://www.theatlantic.com/ideas/archive/2020/06/dictionary-definition-racism-has-change/613324/
https://www.bbc.com/news/world-us-canada-52993306

f_c_r_5

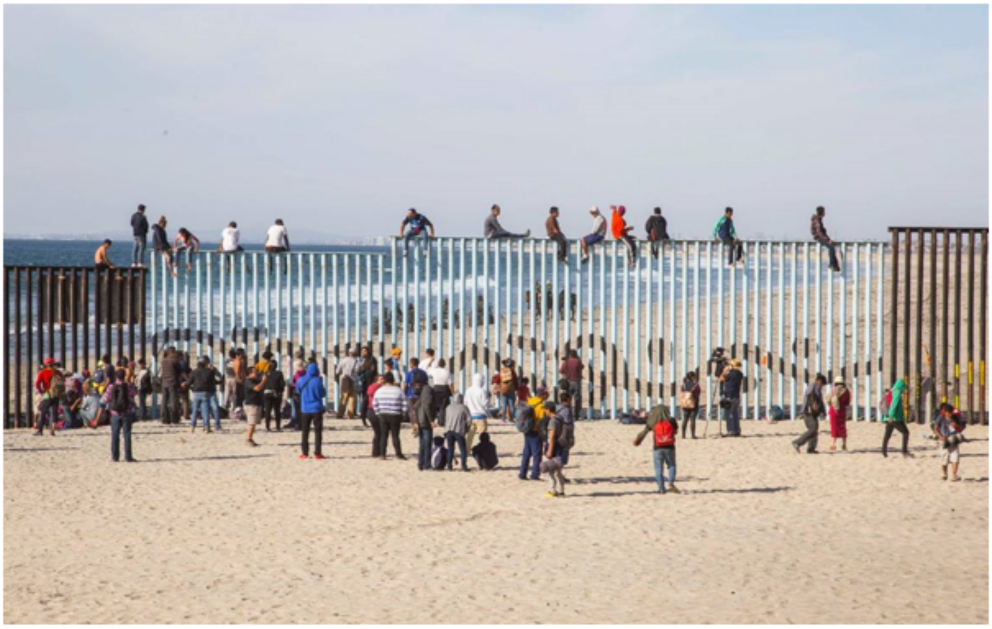

Study estimates 'Mohammed' and 'Juan' will be the two most frequently given names in U.S. by 2040

https://www.snopes.com/fact-check/is-mohammed-popular-name-netherlands/

f_c_r_7

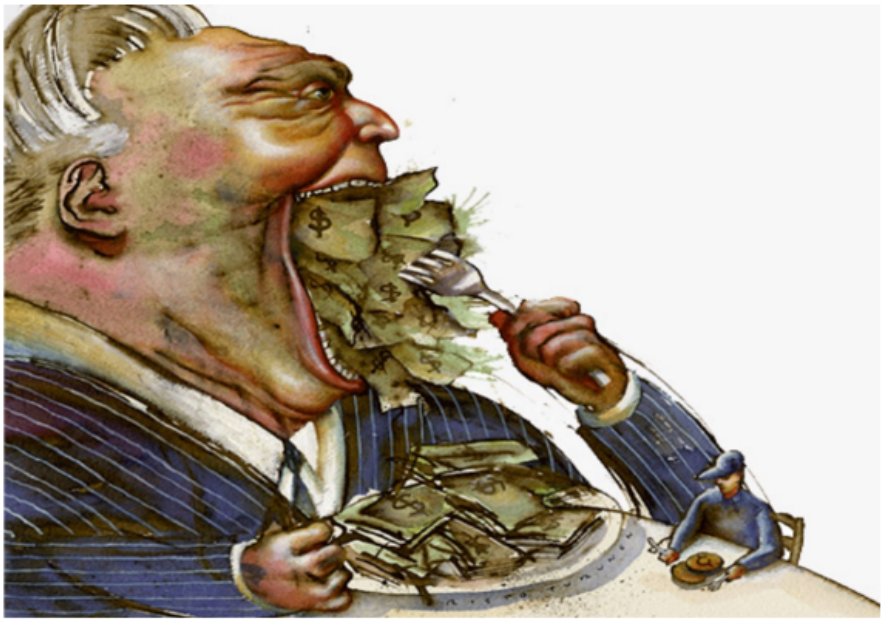

Most climate scientists say human-caused global warming is happening–because that's how they get government grant money

Climate misinformation inspired from DOI: 10.1002/gch2.201600008
Inoculating the Public against Misinformation about Climate Change Sander van der Linden,* Anthony Leiserowitz, Seth Rosenthal, and Edward Maibach

C.5 False, hostile items

f_h_d_2

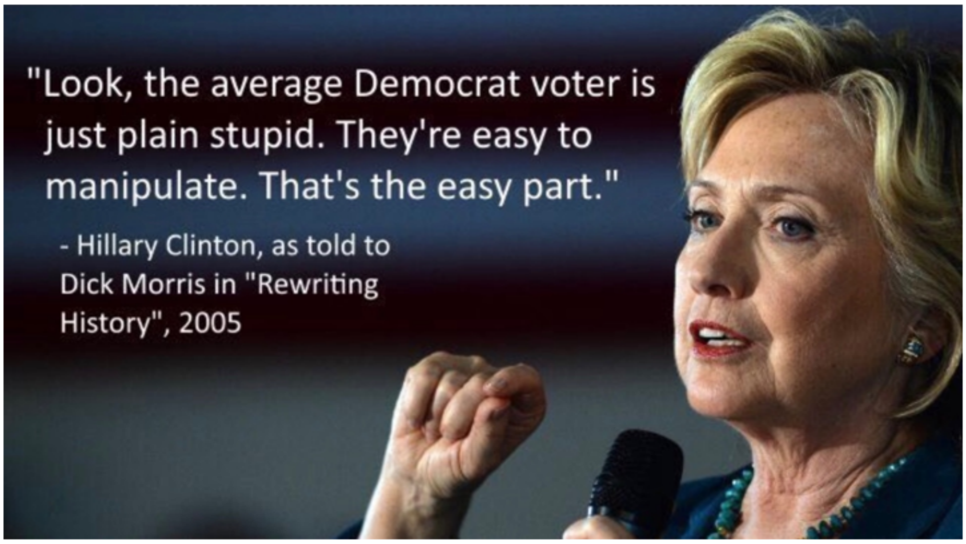

https://twitter.com/SheriffClarke/status/947201898075115520?s=20&t=nMPNarZGfEKPYhVfBsoy3w

f_h_d_10

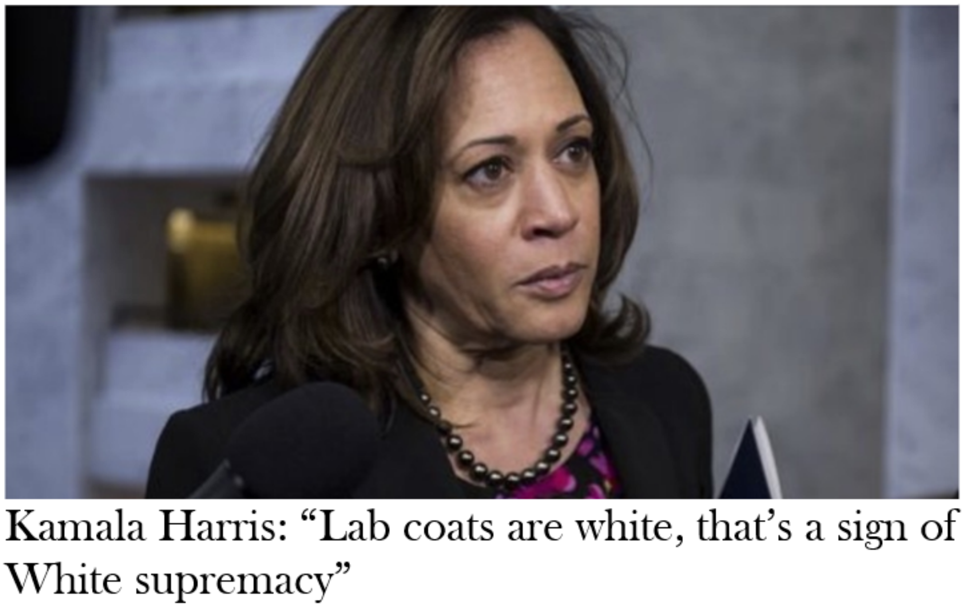

https://genesiustimes.com/kamala-harris-white-lab-coats-a-sign-of-doctors-racism/

f_h_d_14

Captured Islamic State Leader Had Barack Obama On Speed Dial

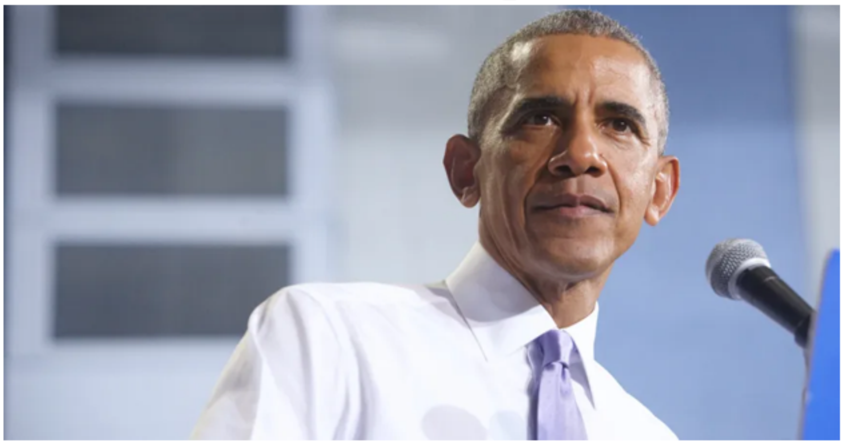

https://www.snopes.com/fact-check/did-captured-islamic-state-obama/

f_h_r_2

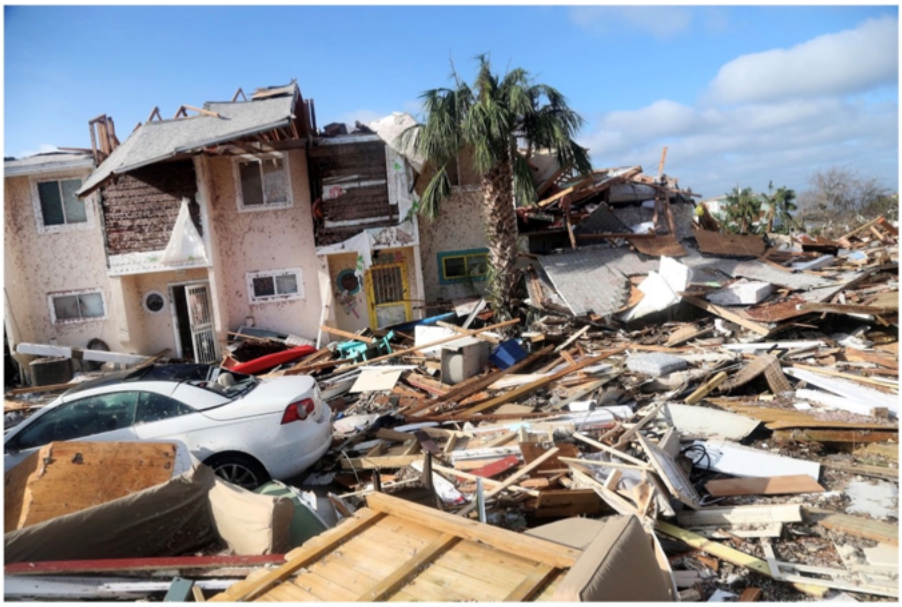

Trump's federal government intentionally breached flood levees in Florida during the 2018 hurricanes season so that poor neighborhoods would be flooded and middle-class neighborhoods would be spared

f_h_r_3

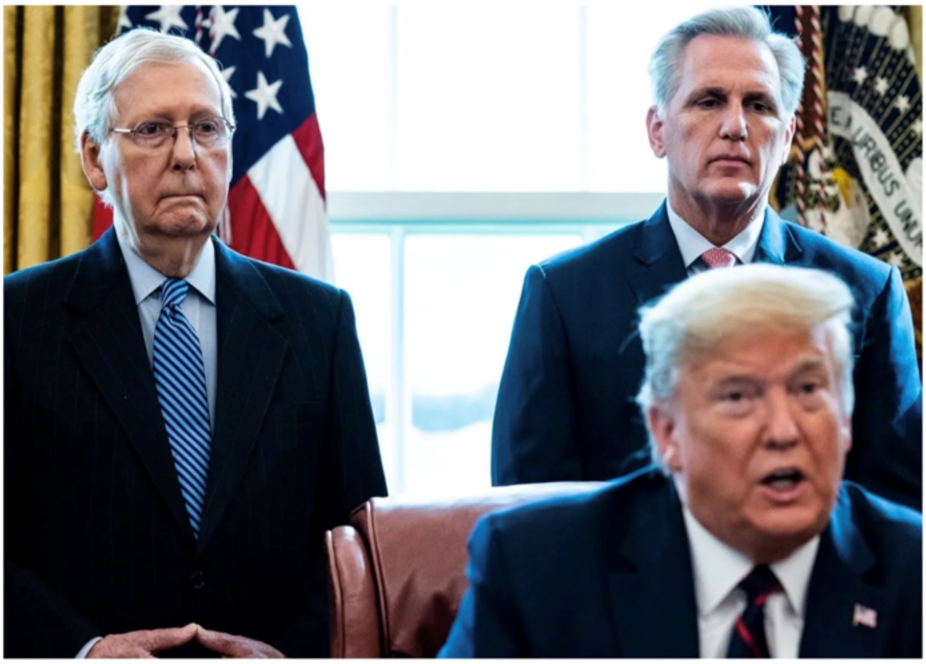

Under the Republican tax bill passed in December 2019, Medicare will stop covering cancer and HIV treatments

f_h_r_5

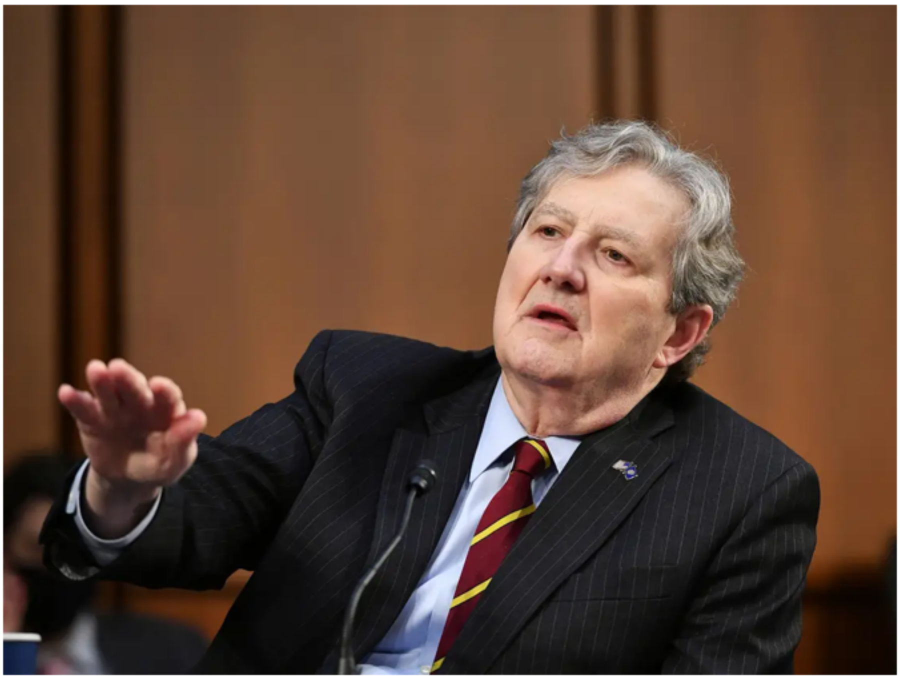

Republican senator of Louisiana gone crazy: “Allowing abortions for rapes will lead women to try to get raped”

C.6 Selection of news items for Study 3

Study 3 used only 2 stories per category of partisan news used in Study 2 (instead of 3), and no neutral items.

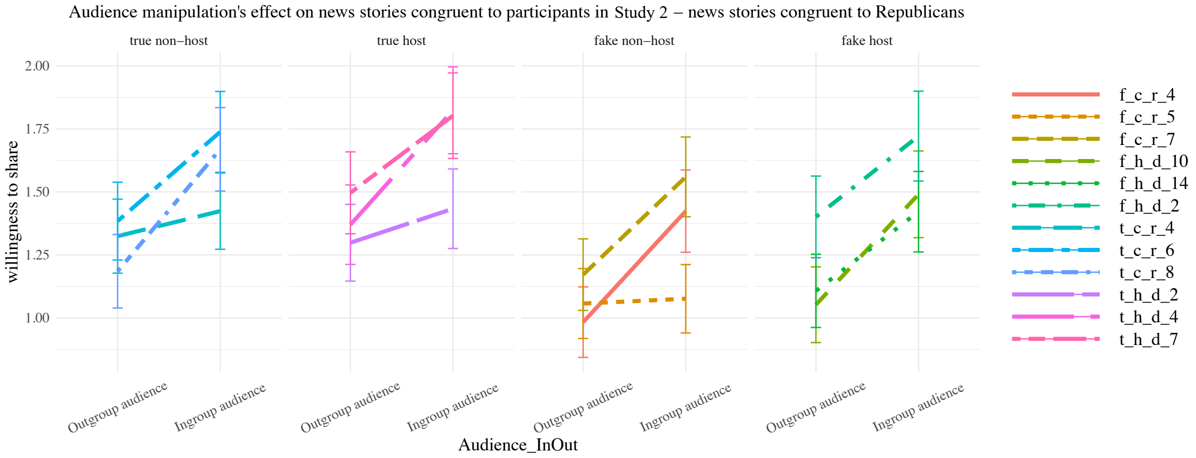

Supplementary figure 7: Effects of the audience manipulation on news stories congruent to Republicans in Study 2.

The following items congruent to Republicans, displaying the strongest audience effects in Study 2, were used in Study 3:

t_c_r_6
t_c_r_8

t_h_d_4
t_h_d_7

f_c_r_7
f_c_r_4

f_h_d_2
f_h_d_10

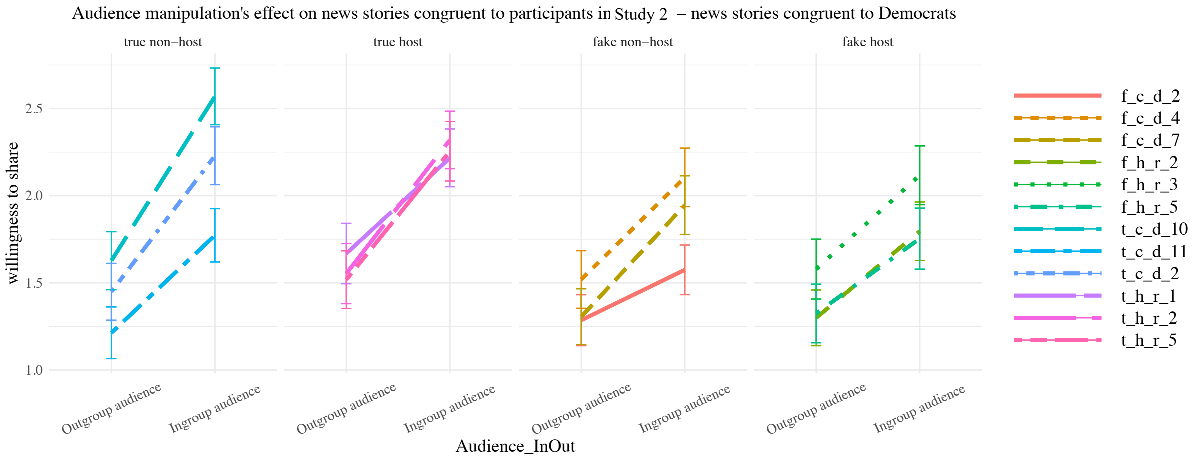

Supplementary figure 8: Effects of the audience manipulation on news stories congruent to Democrats in Study 2.

The following items congruent to Democrats, displaying the strongest audience effects in Study 2, were used in Study 3:
t_c_d_10
t_c_d_2

t_h_r_2
t_h_r_5

f_c_d_4
f_c_d_7

f_h_r_5
f_h_r_2

D. Codebook for regression analyses

Study 1

share: count of news stories shared from a domain on Twitter in Study 1

followers likemindedness: in Study 1, the higher the score, the more politically like-minded to the respondent the Twitter followers were perceived as being

followers: in Study 1, the number of followers each respondent's Twitter account has

source_resp_congruence: degree to which news domain of the news item shared on Twitter is politically congruent to a respondent's partisanship (e.g., pro-Democrat domain if she is Democrat-leaning)

source_netw_congen: degree of political congeniality of news domain from which a news item is shared to the Twitter followers of the respondent

highered: respondent has higher education (dichotomous)

white: respondent is ethnically white (dichotomous)

age: respondent's age (4 groups)
1 = 18-29
2 = 30-44
3 = 45-64
4 = 65+

Studies 2, 3

w2s: willingness to share the news in the experiments Studies 2-3

news_congruence: news item is politically congruent to a respondent's partisanship (e.g., pro-Democrat if she is Democrat-leaning)

Audience InOut [Ingroup audience]: Whether the imagined audience was the ingroup or the outgroup in Study 2. Level as baseline in squared brackets.

Audience InMixOut [Ingroup audience]: Whether the imagined audience was the ingroup or the outgroup or mixed in Study 3. Level as baseline in squared brackets.

E. Analyses of Study 1 (shares, Twitter data)

E.1 Histograms of distributions of shares

Counts of shares from news domains on U.S. Twitter (N=1308)

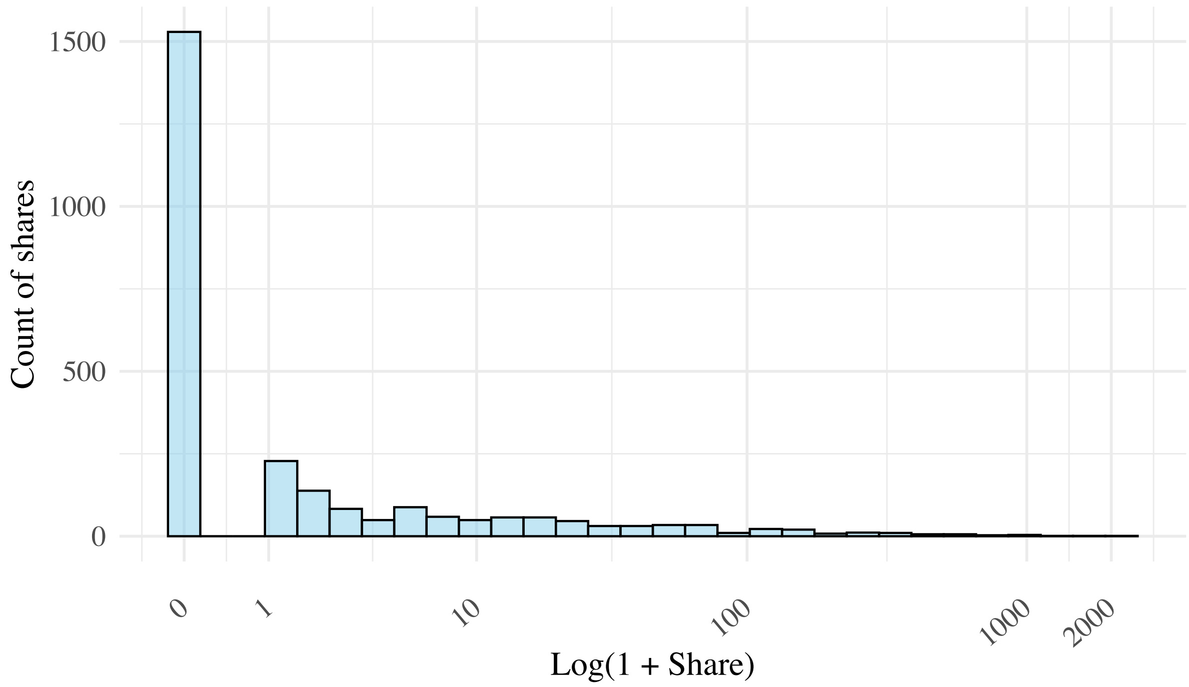

Counts of shares from real news domains on U.S. Twitter (N=1308)

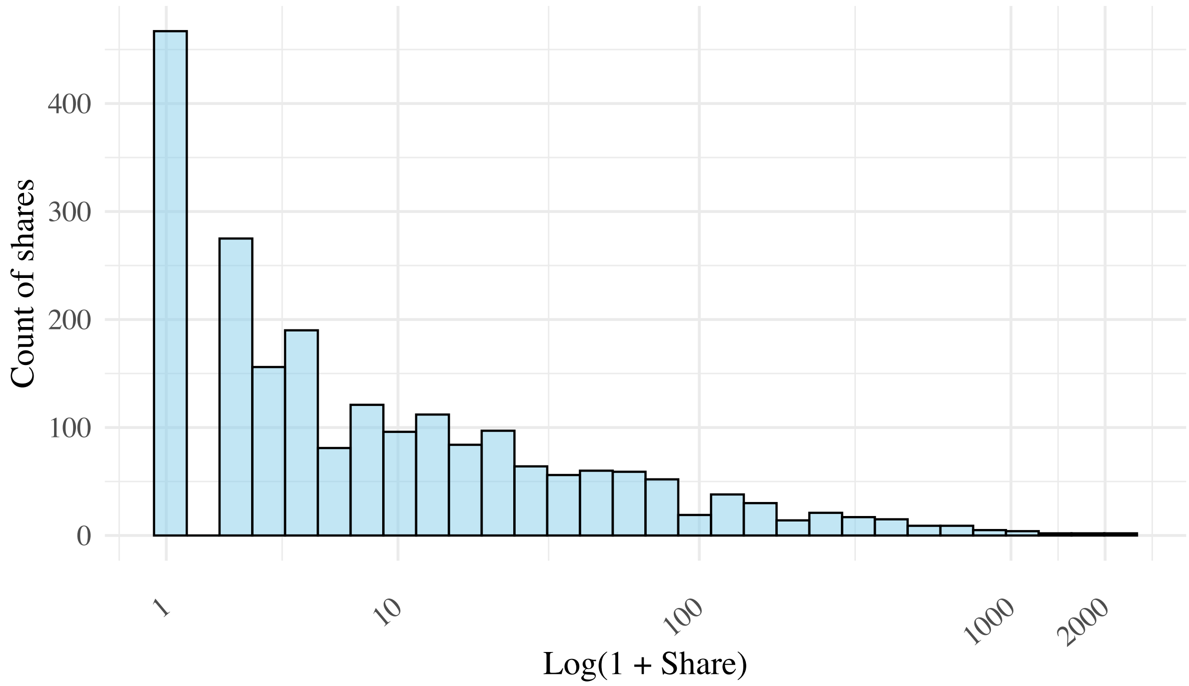

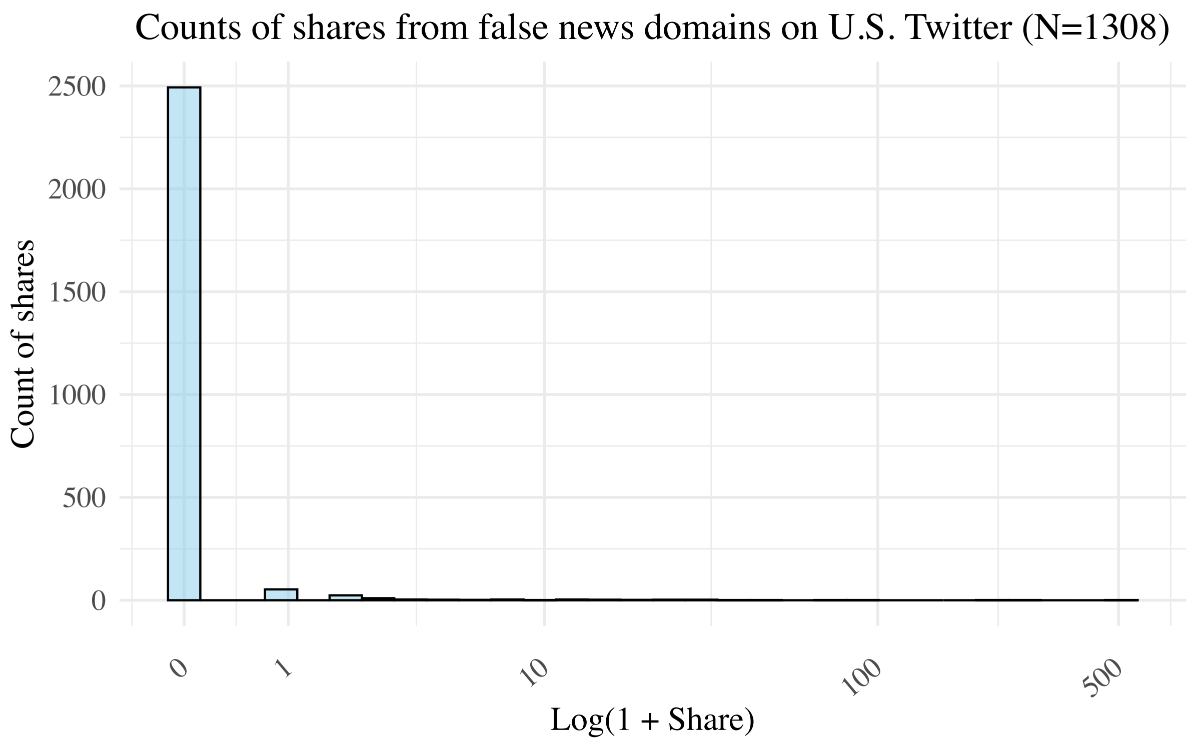

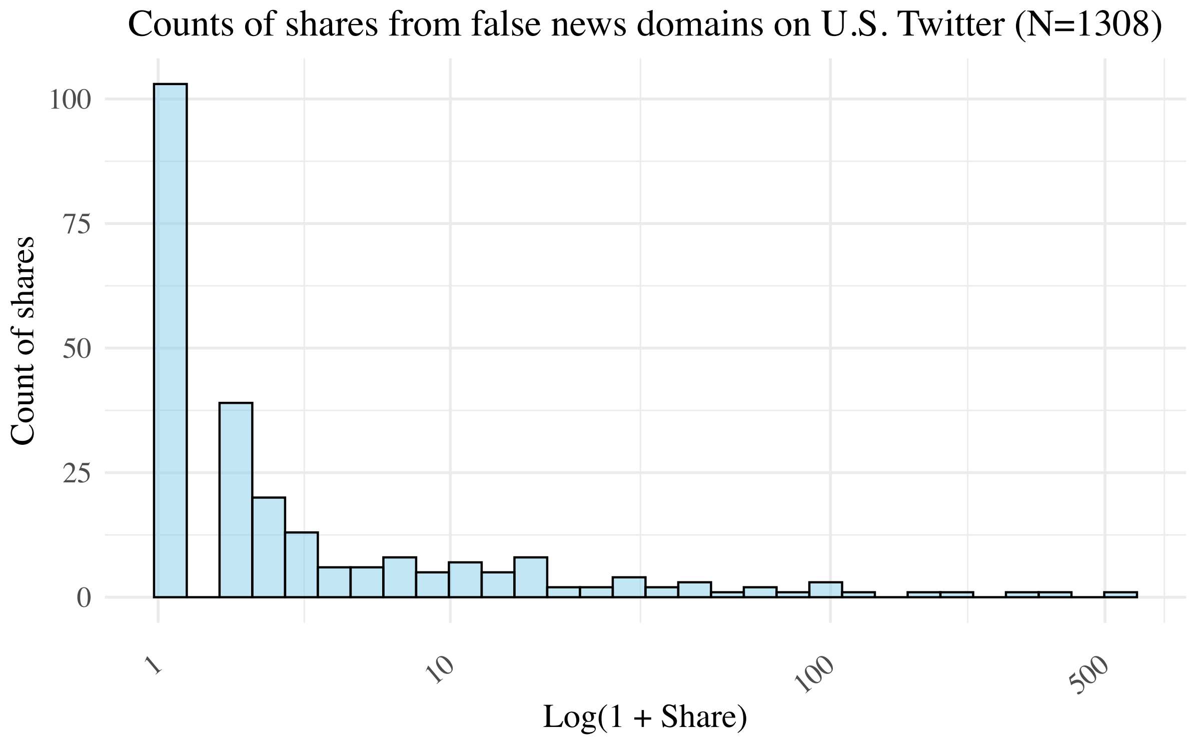

Supplementary figure 9 : histogram of distribution of shares from real and false news domains in Study 1, including and excluding no shares to better visualise the distributions.

E.2 Distributions of like-minded followers among Democrat and Republican Twitter users

Only Democrats and Republican Twitter users were kept in the data, so that it is straightforward to determine whether, say, Democratic followers are like-minded for them or not.
NB: Each row adds to 100%: rows display percentages of followers low vs. high in likemindedness among the total number of followers of Democratic, and then Republican Twitter users, respectively.

Like-mindedness of Twitter followers (1 = low; 7 = high)

partyid_3lev	1	2	3	4	5	6	7
Democrats	0.2%	1.3%	3.1%	19.9%	27.3%	33.1%	15.0%
Republicans	1.5%	3.7%	7.8%	25.2%	24.0%	26.5%	11.3%

E.3 Groupwise numbers of observations of shares from real and false news domains (congruent and incongruent news)

The table below displays groupwise numbers of observations of shares, according to the plotting scheme chosen in the paper for Study 1.

Left columns: real news domains; right columns: false news domains
1 to 7 in left-side columns of each panel denote the degrees to which followers and shares are likeminded
Numbers in round brackets are numbers of observations

Shares from real news domains

followers_like mindedness	Congruent	Incongruent
1	0.3% (16)	0.3% (16)
2	1.0% (54)	1.0% (54)
3	2.3% (120)	2.3% (120)
4	10.8% (564)	10.8% (564)
5	13.1% (688)	13.1% (688)
6	15.5% (812)	15.5% (812)
7	6.9% (362)	6.9% (362)

Shares from false news domains

followers_like mindedness	Congruent	Incongruent
1	0.3% (16)	0.3% (16)
2	1.0% (54)	1.0% (54)
3	2.3% (120)	2.3% (120)
4	10.8% (564)	10.8% (564)
5	13.1% (688)	13.1% (688)
6	15.5% (812)	15.5% (812)
7	6.9% (362)	6.9% (362)

E.4 Regressions: followers' like-mindedness on shares

Effects of Twitter followers' being like-minded for respondents (i.e., from the political ingroup) on shares of congruent and incongruent real news, and then on shares of congruent and incongruent false news.

By default, we follow Osmudsen et al. 2021, Partisan Polarization Is the Primary Psychological Motivation behind Political Fake News Sharing on Twitter, *American Political Science Review,* 115(3), 10.1017/S0003055421000290 in categorizing the news domains as producing real or false stories. The classification of news domains as producing real news is based on the list by the Allsides website (www.allsides.com/media-bias/), and the classification of domains producing false news is based on Allcott, H., Gentzkow, M., & Yu, C. (2019). Trends in the diffusion of misinformation on social media. *Research & Politics*, *6*(2), 2053168019848554.

E.4.1 Congruent real news (main Allsides' classification)

Zero-inflated negative binomial, Log odds:

Stu 1 - followers likemindedness on shares of congruent real partisan news (log)

	share		
Predictors	*Log-Mean*	*CI*	*p*
**Count Model**			
(Intercept)	3.32	2.87,3.78	**<0.001**
followers likemindedness	0.38	0.23,0.54	**<0.001**
female [1]	-0.03	-0.30,0.24	0.846
hh income	0.09	-0.07,0.25	0.263
highered [1]	0.08	-0.23,0.38	0.624
age [2]	0.08	-0.35,0.52	0.705
age [3]	0.50	0.07,0.93	**0.023**
age [4]	0.82	0.22,1.42	**0.007**
followers	0.24	-0.10,0.57	0.163
partyid	-0.01	-0.08,0.05	0.695
**Zero-Inflated Model**			
(Intercept)	-55.90	-76.35,-35.44	**<0.001**
followers likemindedness	0.06	-0.21,0.33	0.652

female [1]	0.74	0.19,1.30	**0.008**
hh income	0.28	-0.01,0.57	0.057
highered [1]	-0.05	-0.61,0.51	0.862
age [2]	-0.95	-1.87,-0.03	**0.044**
age [3]	-1.55	-2.44,-0.65	**0.001**
age [4]	-2.13	-3.27,-0.99	**<0.001**
followers	-462.69	-629.50,-295.88	**<0.001**
partyid	0.20	0.08,0.32	**0.001**
Observations	1308		
R^2 / R^2 adjusted	1.000 / 1.000		

Zero-inflated negative binomial, Exponentiated coefficients:

Stu 1 - followers likemindedness on shares of congruent real partisan news (expon.)

	share		
Predictors	*Incidence Rate Ratios*	*CI*	*p*
**Count Model**			
(Intercept)	27.77	17.67,43.63	**<0.001**
followers likemindedness	1.47	1.26,1.71	**<0.001**
female [1]	0.97	0.74,1.28	0.846
hh income	1.10	0.93,1.29	0.263
highered [1]	1.08	0.80,1.46	0.624
age [2]	1.09	0.70,1.69	0.705
age [3]	1.65	1.07,2.54	**0.023**
age [4]	2.27	1.24,4.13	**0.007**
followers	1.27	0.91,1.77	0.163
partyid	0.99	0.92,1.06	0.695
**Zero-Inflated Model**			
(Intercept)	0.00	0.00,0.00	**<0.001**

followers likemindedness	1.06	0.81,1.40	0.652
female [1]	2.10	1.21,3.66	**0.008**
hh income	1.32	0.99,1.76	0.057
highered [1]	0.95	0.54,1.66	0.862
age [2]	0.39	0.15,0.98	**0.044**
age [3]	0.21	0.09,0.52	**0.001**
age [4]	0.12	0.04,0.37	**<0.001**
followers	0.00	0.00,0.00	**<0.001**
partyid	1.22	1.08,1.38	**0.001**
Observations	1308		
R^2 / R^2 adjusted	1.000 / 1.000		

Zero-inflated negative binomial, exponentiated coefficients, without 1% greatest sharers

Stu 1 - followers likemindedness on shares of congruent real partisan news (expon.)

	share		
Predictors	*Incidence Rate Ratios*	*CI*	*p*
**Count Model**			
(Intercept)	19.03	12.35,29.34	**<0.001**
followers likemindedness	1.30	1.14,1.47	**<0.001**
female [1]	1.02	0.79,1.31	0.884
hh income	0.98	0.86,1.11	0.706
highered [1]	1.18	0.89,1.57	0.244
age [2]	1.00	0.67,1.50	0.996
age [3]	1.53	1.03,2.27	**0.033**
age [4]	1.40	0.81,2.42	0.224
followers	1.14	0.92,1.41	0.229
partyid	0.94	0.88,0.99	**0.026**

Zero-Inflated Model

(Intercept)	0.00	0.00,0.00	**<0.001**
followers likemindedness	1.02	0.79,1.32	0.853
female [1]	1.98	1.18,3.31	**0.010**
hh income	1.24	0.95,1.63	0.113
highered [1]	1.00	0.59,1.70	0.999
age [2]	0.39	0.16,0.91	**0.029**
age [3]	0.22	0.09,0.51	**<0.001**
age [4]	0.12	0.04,0.34	**<0.001**
followers	0.00	0.00,0.00	**<0.001**
partyid	1.21	1.08,1.35	**0.001**
Observations	1282		
R^2 / R^2 adjusted	0.998 / 0.998		

Negative binomial, Exponentiated coefficients:

Stu 1 - followers likemindedness on shares of congruent real partisan news (expon.)

	share		
Predictors	*Incidence Rate Ratios*	*CI*	*p*
(Intercept)	20.61	11.51,38.09	**<0.001**
followers likemindedness	1.44	1.21,1.70	**<0.001**
female [1]	0.86	0.64,1.16	0.316
white [1]	1.01	0.70,1.45	0.935
hh income	1.11	0.93,1.33	0.194
highered [1]	1.17	0.84,1.62	0.350
age [2]	1.18	0.73,1.85	0.478
age [3]	1.71	1.06,2.68	**0.019**
age [4]	2.47	1.29,4.89	**0.005**
followers	3.04		**<0.001**

partyid	0.94	0.88,1.01	0.058
Observations	1308		
R^2 Nagelkerke	0.076		

Poisson regression, non-exponentiated coefficients

Stu 1 - followers likemindedness on shares of congruent real partisan news (Poisson)(log)

	share		
Predictors	*Log-Mean*	*CI*	*p*
(Intercept)	2.83	2.78,2.87	**<0.001**
followers likemindedness	0.34	0.33,0.35	**<0.001**
female [1]	-0.07	-0.09,-0.05	**<0.001**
white [1]	0.08	0.05,0.10	**<0.001**
hh income	0.06	0.05,0.07	**<0.001**
highered [1]	0.27	0.24,0.29	**<0.001**
age [2]	0.08	0.04,0.12	**<0.001**
age [3]	0.69	0.66,0.73	**<0.001**
age [4]	0.96	0.92,1.01	**<0.001**
followers	0.06	0.06,0.07	**<0.001**
partyid	-0.05	-0.06,-0.05	**<0.001**
Observations	1308		
R^2 Nagelkerke	1.000		

Poisson regression, exponentiated coefficients

Stu 1 - followers likemindedness on shares of congruent real partisan news (Poisson)(expon.)

	share		
Predictors	*Incidence Rate Ratios*	*CI*	*p*
(Intercept)	16.86	16.12,17.63	**<0.001**

followers likemindedness	1.40	1.39,1.42	**<0.001**
female [1]	0.93	0.92,0.95	**<0.001**
white [1]	1.08	1.05,1.11	**<0.001**
hh income	1.06	1.05,1.07	**<0.001**
highered [1]	1.31	1.28,1.34	**<0.001**
age [2]	1.09	1.04,1.13	**<0.001**
age [3]	2.00	1.93,2.07	**<0.001**
age [4]	2.62	2.51,2.73	**<0.001**
followers	1.07	1.06,1.07	**<0.001**
partyid	0.95	0.94,0.95	**<0.001**
Observations	1308		
R^2 Nagelkerke	1.000		

E.4.2 Congruent real news (Bakshy's alternative classification)

Zero-inflated negative binomial fit, non-exponentiated coefficients

Stu 1 - followers likemindedness on shares of congruent real partisan news (log)

	share		
Predictors	*Log-Mean*	*CI*	*p*
**Count Model**			
(Intercept)	3.18	2.73,3.64	**<0.001**
followers likemindedness	0.33	0.17,0.49	**<0.001**
female [1]	-0.05	-0.32,0.23	0.752
hh income	0.11	-0.05,0.27	0.185
highered [1]	0.10	-0.21,0.41	0.518
age [2]	0.12	-0.32,0.57	0.595
age [3]	0.55	0.11,0.98	**0.015**
age [4]	0.97	0.35,1.59	**0.002**
followers	0.27	-0.09,0.63	0.143
partyid	-0.01	-0.08,0.06	0.760
**Zero-Inflated Model**			
(Intercept)	-66.90	-94.86,-38.95	**<0.001**
followers likemindedness	0.06	-0.23,0.34	0.693
female [1]	0.58	0.01,1.15	**0.046**
hh income	0.19	-0.10,0.49	0.204
highered [1]	-0.30	-0.89,0.30	0.332
age [2]	-0.67	-1.63,0.29	0.174
age [3]	-1.09	-2.01,-0.17	**0.021**
age [4]	-1.34	-2.50,-0.18	**0.024**
followers	-551.49	-777.98,-324.99	**<0.001**
partyid	0.14	0.01,0.26	**0.033**
Observations	1308		

R^2 / R^2 adjusted	1.000 / 1.000

Zero-inflated negative binomial fit, exponentiated coefficients

Stu 1 - followers likemindedness on shares of congruent real partisan news (expon.)

	**share**		
Predictors	*Incidence Rate Ratios*	*CI*	*p*
**Count Model**			
(Intercept)	24.16	15.29,38.16	**<0.001**
followers likemindedness	1.39	1.19,1.64	**<0.001**
female [1]	0.96	0.72,1.26	0.752
hh income	1.11	0.95,1.31	0.185
highered [1]	1.11	0.81,1.51	0.518
age [2]	1.13	0.72,1.76	0.595
age [3]	1.72	1.11,2.68	**0.015**
age [4]	2.64	1.42,4.92	**0.002**
followers	1.31	0.91,1.88	0.143
partyid	0.99	0.92,1.06	0.760
**Zero-Inflated Model**			
(Intercept)	0.00	0.00,0.00	**<0.001**
followers likemindedness	1.06	0.80,1.41	0.693
female [1]	1.79	1.01,3.16	**0.046**
hh income	1.21	0.90,1.63	0.204
highered [1]	0.74	0.41,1.35	0.332
age [2]	0.51	0.20,1.34	0.174
age [3]	0.34	0.13,0.85	**0.021**
age [4]	0.26	0.08,0.84	**0.024**
followers	0.00	0.00,0.00	**<0.001**

partyid	1.15	1.01,1.30	**0.033**
Observations	1308		
R^2 / R^2 adjusted	1.000 / 1.000		

Zero-inflated negative binomial fit, non-exponentiated coefficients, without 1% greatest sharers

**Stu 1 - followers likemindedness on shares of congruent real partisan news (log)**			
		**share**	
Predictors	*Log-Mean*	*CI*	*p*
**Count Model**			
(Intercept)	3.03	2.57,3.48	**<0.001**
followers likemindedness	0.23	0.10,0.36	**0.001**
female [1]	-0.17	-0.43,0.10	0.214
hh income	0.09	-0.05,0.22	0.222
highered [1]	0.18	-0.11,0.48	0.221
age [2]	-0.19	-0.60,0.22	0.369
age [3]	0.44	0.04,0.83	**0.032**
age [4]	0.44	-0.12,1.01	0.124
followers	0.24	-0.01,0.49	0.061
partyid	-0.09	-0.15,-0.03	**0.004**
**Zero-Inflated Model**			
(Intercept)	-60.19	-87.11,-33.27	**<0.001**
followers likemindedness	0.03	-0.25,0.30	0.832
female [1]	0.51	-0.04,1.06	0.067
hh income	0.17	-0.12,0.46	0.242
highered [1]	-0.24	-0.81,0.34	0.419
age [2]	-0.75	-1.68,0.17	0.110
age [3]	-1.12	-2.00,-0.23	**0.014**

age [4]	-1.42	-2.55,-0.30	**0.013**
followers	-498.38	-716.62,-280.14	**<0.001**
partyid	0.12	-0.00,0.24	0.057
Observations	1282		
R^2 / R^2 adjusted	1.000 / 1.000		

Zero-inflated negative binomial fit, exponentiated coefficients, without 1% greatest sharers

Stu 1 - followers likemindedness on shares of congruent real partisan news (expon.)

	share		
Predictors	*Incidence Rate Ratios*	*CI*	*p*
**Count Model**			
(Intercept)	20.64	13.13,32.43	**<0.001**
followers likemindedness	1.26	1.10,1.43	**0.001**
female [1]	0.85	0.65,1.10	0.214
hh income	1.09	0.95,1.25	0.222
highered [1]	1.20	0.90,1.61	0.221
age [2]	0.83	0.55,1.25	0.369
age [3]	1.55	1.04,2.30	**0.032**
age [4]	1.56	0.89,2.73	0.124
followers	1.27	0.99,1.63	0.061
partyid	0.92	0.86,0.97	**0.004**
**Zero-Inflated Model**			
(Intercept)	0.00	0.00,0.00	**<0.001**
followers likemindedness	1.03	0.78,1.36	0.832
female [1]	1.67	0.96,2.87	0.067
hh income	1.19	0.89,1.58	0.242
highered [1]	0.79	0.44,1.40	0.419
age [2]	0.47	0.19,1.19	0.110

age [3]	0.33	0.13,0.80	**0.014**
age [4]	0.24	0.08,0.74	**0.013**
followers	0.00	0.00,0.00	**<0.001**
partyid	1.13	1.00,1.27	0.057
Observations	1282		
R^2 / R^2 adjusted	1.000 / 1.000		

Negative binomial, Exponentiated coefficients:

Stu 1 - followers likemindedness on shares of congruent real partisan news (expon.)

	**share**		
Predictors	*Incidence Rate Ratios*	*CI*	*p*
(Intercept)	17.15	9.53,31.88	**<0.001**
followers likemindedness	1.36	1.14,1.61	**<0.001**
female [1]	0.86	0.63,1.16	0.319
white [1]	1.07	0.73,1.53	0.717
hh income	1.15	0.96,1.38	0.079
highered [1]	1.26	0.90,1.74	0.177
age [2]	1.18	0.72,1.87	0.478
age [3]	1.69	1.04,2.67	**0.023**
age [4]	2.53	1.31,5.05	**0.005**
followers	3.10		**<0.001**
partyid	0.95	0.89,1.02	0.146
Observations	1308		
R^2 Nagelkerke	0.073		

Poisson, non-exponentiated coefficients

Stu 1 - followers likemindedness on shares of congruent real partisan news (Poisson)(log)

	**share**		
Predictors	*Log-Mean*	*CI*	*p*

(Intercept)	2.70	2.65,2.74	**<0.001**
followers likemindedness	0.31	0.30,0.32	**<0.001**
female [1]	-0.06	-0.08,-0.04	**<0.001**
white [1]	0.10	0.07,0.12	**<0.001**
hh income	0.07	0.06,0.08	**<0.001**
highered [1]	0.33	0.30,0.35	**<0.001**
age [2]	0.03	-0.00,0.07	0.086
age [3]	0.65	0.62,0.69	**<0.001**
age [4]	1.01	0.96,1.05	**<0.001**
followers	0.06	0.06,0.07	**<0.001**
partyid	-0.04	-0.04,-0.03	**<0.001**
Observations	1308		
R^2 Nagelkerke	0.999		

Poisson, exponentiated coefficients

Stu 1 - followers likemindedness on shares of congruent real partisan news (Poisson)(expon.)

	share		
Predictors	*Incidence Rate Ratios*	*CI*	*p*
(Intercept)	14.85	14.18,15.56	**<0.001**
followers likemindedness	1.36	1.34,1.37	**<0.001**
female [1]	0.94	0.92,0.96	**<0.001**
white [1]	1.10	1.07,1.13	**<0.001**
hh income	1.07	1.06,1.09	**<0.001**
highered [1]	1.38	1.35,1.42	**<0.001**
age [2]	1.04	1.00,1.08	0.086
age [3]	1.92	1.85,2.00	**<0.001**
age [4]	2.73	2.62,2.86	**<0.001**
followers	1.07	1.06,1.07	**<0.001**

partyid		0.96	0.96,0.97	**<0.001**
Observations	1308			
R^2 Nagelkerke	0.999			

E.4.3 Incongruent real news (main Allsides' classification)

Zero-inflated negative binomial, Log odds:

Stu 1 - followers likemindedness on shares of incongruent real partisan news (log)

	share		
Predictors	*Log-Mean*	*CI*	*p*
**Count Model**			
(Intercept)	-1.61	-2.18,-1.05	**<0.001**
followers likemindedness	-0.30	-0.44,-0.17	**<0.001**
female [1]	-0.08	-0.39,0.23	0.624
hh income	0.18	0.03,0.32	**0.015**
highered [1]	0.83	0.50,1.16	**<0.001**
age [2]	0.66	0.18,1.15	**0.007**
age [3]	0.98	0.51,1.45	**<0.001**
age [4]	1.11	0.44,1.78	**0.001**
followers	0.03	-0.19,0.25	0.798
partyid	0.38	0.31,0.45	**<0.001**
**Zero-Inflated Model**			
(Intercept)	-39.74	-53.44,-26.04	**<0.001**
followers likemindedness	0.08	-0.23,0.40	0.604
female [1]	-0.26	-0.94,0.42	0.450
hh income	0.29	-0.07,0.65	0.120
highered [1]	0.65	-0.06,1.36	0.074
age [2]	0.62	-0.63,1.88	0.329
age [3]	0.51	-0.68,1.71	0.400
age [4]	0.29	-1.23,1.81	0.709
followers	-336.94	-449.27,-224.62	**<0.001**
partyid	-0.23	-0.38,-0.09	**0.002**

Observations	1308
R^2 / R^2 adjusted	0.950 / 0.950

Zero-inflated negative binomial, Exponentiated coefficients:

Stu 1 - followers likemindedness on shares of incongruent real partisan news (expon.)

	**share**		
Predictors	*Incidence Rate Ratios*	*CI*	*p*
**Count Model**			
(Intercept)	0.20	0.11,0.35	**<0.001**
followers likemindedness	0.74	0.65,0.85	**<0.001**
female [1]	0.93	0.68,1.26	0.624
hh income	1.20	1.04,1.38	**0.015**
highered [1]	2.30	1.65,3.20	**<0.001**
age [2]	1.94	1.20,3.15	**0.007**
age [3]	2.67	1.67,4.27	**<0.001**
age [4]	3.04	1.55,5.95	**0.001**
followers	1.03	0.82,1.29	0.798
partyid	1.47	1.37,1.57	**<0.001**
**Zero-Inflated Model**			
(Intercept)	0.00	0.00,0.00	**<0.001**
followers likemindedness	1.09	0.79,1.50	0.604
female [1]	0.77	0.39,1.52	0.450
hh income	1.33	0.93,1.91	0.120
highered [1]	1.91	0.94,3.90	0.074
age [2]	1.87	0.53,6.54	0.329
age [3]	1.67	0.50,5.55	0.400
age [4]	1.34	0.29,6.12	0.709

followers	0.00	0.00,0.00	**<0.001**
partyid	0.79	0.68,0.92	**0.002**
Observations	1308		
R^2 / R^2 adjusted	0.950 / 0.950		

Zero-inflated negative binomial, log coefficients without 1% greatest sharers:

Stu 1 - followers likemindedness on shares of incongruent real partisan news (log)

	share		
Predictors	*Log-Mean*	*CI*	*p*
**Count Model**			
(Intercept)	-1.57	-2.12,-1.01	**<0.001**
followers likemindedness	-0.22	-0.36,-0.08	**0.002**
female [1]	-0.05	-0.35,0.26	0.769
hh income	0.12	-0.02,0.26	0.104
highered [1]	0.80	0.48,1.12	**<0.001**
age [2]	0.58	0.11,1.05	**0.015**
age [3]	1.00	0.54,1.46	**<0.001**
age [4]	1.12	0.46,1.78	**0.001**
followers	0.02	-0.18,0.23	0.829
partyid	0.37	0.30,0.44	**<0.001**
**Zero-Inflated Model**			
(Intercept)	-39.48	-53.19,-25.77	**<0.001**
followers likemindedness	0.12	-0.20,0.44	0.467
female [1]	-0.23	-0.90,0.44	0.502
hh income	0.26	-0.10,0.62	0.155
highered [1]	0.62	-0.08,1.33	0.084
age [2]	0.56	-0.68,1.81	0.377
age [3]	0.51	-0.68,1.70	0.400

age [4]	0.30	-1.21,1.81	0.702
followers	-335.24	-447.56,-222.92	**<0.001**
partyid	-0.24	-0.38,-0.09	**0.001**
Observations	1307		
R^2 / R^2 adjusted	0.924 / 0.924		

Zero-inflated negative binomial, Exponentiated coefficients without 1% greatest sharers:

Stu 1 - followers likemindedness on shares of incongruent real partisan news (expon.)

	share		
Predictors	*Incidence Rate Ratios*	*CI*	*p*
**Count Model**			
(Intercept)	0.21	0.12,0.36	**<0.001**
followers likemindedness	0.80	0.70,0.92	**0.002**
female [1]	0.96	0.71,1.29	0.769
hh income	1.13	0.98,1.30	0.104
highered [1]	2.22	1.61,3.08	**<0.001**
age [2]	1.78	1.12,2.85	**0.015**
age [3]	2.72	1.72,4.30	**<0.001**
age [4]	3.06	1.59,5.91	**0.001**
followers	1.02	0.83,1.26	0.829
partyid	1.45	1.35,1.55	**<0.001**
**Zero-Inflated Model**			
(Intercept)	0.00	0.00,0.00	**<0.001**
followers likemindedness	1.13	0.82,1.55	0.467
female [1]	0.79	0.40,1.56	0.502
hh income	1.30	0.91,1.86	0.155
highered [1]	1.87	0.92,3.79	0.084
age [2]	1.75	0.50,6.08	0.377

age [3]	1.67	0.51,5.48	0.400
age [4]	1.34	0.30,6.08	0.702
followers	0.00	0.00,0.00	**<0.001**
partyid	0.79	0.68,0.91	**0.001**
Observations	1307		
R^2 / R^2 adjusted	0.924 / 0.924		

Negative binomial, Exponentiated coefficients:

Stu 1 - followers likemindedness on shares of incongruent real partisan news (expon.)

	share		
Predictors	*Incidence Rate Ratios*	*CI*	*p*
(Intercept)	0.11	0.06,0.22	**<0.001**
followers likemindedness	0.75	0.65,0.87	**<0.001**
female [1]	0.98	0.70,1.35	0.878
white [1]	1.24	0.83,1.83	0.270
hh income	1.20	1.02,1.40	**0.031**
highered [1]	2.20	1.55,3.10	**<0.001**
age [2]	1.71	1.02,2.82	**0.037**
age [3]	2.17	1.30,3.54	**0.002**
age [4]	2.37	1.20,4.81	**0.014**
followers	2.44		**<0.001**
partyid	1.48	1.37,1.59	**<0.001**
Observations	1308		
R^2 Nagelkerke	0.362		

Poisson regression, non-exponentiated coefficients

Stu 1 - followers likemindedness on shares of incongruent real partisan news (Poisson)(log)

	share		

Predictors	Log-Mean	CI	p
(Intercept)	-3.28	-3.49,-3.07	**<0.001**
followers likemindedness	-0.54	-0.56,-0.51	**<0.001**
female [1]	-0.61	-0.68,-0.53	**<0.001**
white [1]	0.61	0.51,0.72	**<0.001**
hh income	0.33	0.29,0.36	**<0.001**
highered [1]	1.15	1.05,1.25	**<0.001**
age [2]	1.21	1.06,1.36	**<0.001**
age [3]	0.60	0.45,0.75	**<0.001**
age [4]	0.82	0.65,0.99	**<0.001**
partyid	0.47	0.45,0.49	**<0.001**
followers	0.02	0.00,0.03	**0.020**
Observations	1308		
R^2 Nagelkerke	0.999		

Poisson regression, exponentiated coefficients

Stu 1 - followers likemindedness on shares of incongruent real partisan news (Poisson)(expon.)

	share		
Predictors	*Incidence Rate Ratios*	*CI*	*p*
(Intercept)	0.04	0.03,0.05	**<0.001**
followers likemindedness	0.58	0.57,0.60	**<0.001**
female [1]	0.55	0.51,0.59	**<0.001**
white [1]	1.85	1.67,2.05	**<0.001**
hh income	1.39	1.34,1.44	**<0.001**
highered [1]	3.15	2.85,3.48	**<0.001**
age [2]	3.34	2.89,3.88	**<0.001**
age [3]	1.82	1.57,2.11	**<0.001**
age [4]	2.27	1.91,2.70	**<0.001**

partyid		1.60	1.57,1.63	**<0.001**
followers		1.02	1.00,1.03	**0.020**
Observations	1308			
R^2 Nagelkerke	0.999			

E.4.4 Incongruent real news (Bakshy's alternative classification)

Zero-inflated negative binomial fit, non-exponentiated coefficients

Stu 1 - followers likemindedness on shares of incongruent real partisan news (log)

	share		
Predictors	*Log-Mean*	*CI*	*p*
**Count Model**			
(Intercept)	-0.74	-1.31,-0.17	**0.011**
followers likemindedness	-0.34	-0.48,-0.19	**<0.001**
female [1]	-0.24	-0.56,0.08	0.146
hh income	0.27	0.12,0.43	**0.001**
highered [1]	0.94	0.59,1.29	**<0.001**
age [2]	0.23	-0.27,0.72	0.369
age [3]	0.48	-0.01,0.96	0.053
age [4]	0.53	-0.17,1.24	0.139
partyid	0.27	0.20,0.34	**<0.001**
followers	0.17	-0.07,0.41	0.162
**Zero-Inflated Model**			
(Intercept)	-30.68	-41.61,-19.75	**<0.001**
followers likemindedness	0.12	-0.19,0.43	0.440
female [1]	-0.09	-0.73,0.55	0.786
hh income	0.19	-0.15,0.53	0.268
highered [1]	0.71	0.00,1.41	**0.049**
age [2]	-0.05	-1.20,1.10	0.931
age [3]	-0.05	-1.15,1.05	0.933
age [4]	0.07	-1.39,1.53	0.925
partyid	-0.16	-0.31,-0.02	**0.024**
followers	-262.93	-351.22,-174.63	**<0.001**

Observations	1308
R^2 / R^2 adjusted	0.999 / 0.999

Zero-inflated negative binomial fit, exponentiated coefficients

Stu 1 - followers likemindedness on shares of incongruent real partisan news (expon.)

	share		
Predictors	*Incidence Rate Ratios*	*CI*	*p*
**Count Model**			
(Intercept)	0.48	0.27,0.85	**0.011**
followers likemindedness	0.71	0.62,0.83	**<0.001**
female [1]	0.79	0.57,1.09	0.146
hh income	1.31	1.13,1.53	**0.001**
highered [1]	2.57	1.81,3.64	**<0.001**
age [2]	1.25	0.77,2.05	0.369
age [3]	1.61	0.99,2.61	0.053
age [4]	1.71	0.84,3.47	0.139
partyid	1.31	1.22,1.41	**<0.001**
followers	1.18	0.93,1.50	0.162
**Zero-Inflated Model**			
(Intercept)	0.00	0.00,0.00	**<0.001**
followers likemindedness	1.13	0.83,1.54	0.440
female [1]	0.92	0.48,1.73	0.786
hh income	1.21	0.86,1.70	0.268
highered [1]	2.03	1.00,4.11	**0.049**
age [2]	0.95	0.30,3.01	0.931
age [3]	0.95	0.32,2.86	0.933
age [4]	1.07	0.25,4.61	0.925

partyid	0.85	0.74,0.98	**0.024**
followers	0.00	0.00,0.00	**<0.001**
Observations	1308		
R^2 / R^2 adjusted	0.999 / 0.999		

Zero-inflated negative binomial fit, non-exponentiated coefficients, without 1% greatest sharers

Stu 1 - followers likemindedness on shares of incongruent real partisan news (log)

	share		
Predictors	*Log-Mean*	*CI*	*p*
**Count Model**			
(Intercept)	-0.70	-1.27,-0.14	**0.015**
followers likemindedness	-0.28	-0.43,-0.12	**<0.001**
female [1]	-0.22	-0.53,0.10	0.179
hh income	0.23	0.07,0.39	**0.004**
highered [1]	0.92	0.57,1.26	**<0.001**
age [2]	0.18	-0.31,0.66	0.478
age [3]	0.50	0.02,0.98	**0.042**
age [4]	0.54	-0.16,1.24	0.131
partyid	0.26	0.18,0.33	**<0.001**
followers	0.17	-0.07,0.40	0.164
**Zero-Inflated Model**			
(Intercept)	-30.52	-41.49,-19.55	**<0.001**
followers likemindedness	0.15	-0.16,0.46	0.355
female [1]	-0.08	-0.71,0.56	0.810
hh income	0.17	-0.16,0.51	0.314
highered [1]	0.69	-0.01,1.39	0.054
age [2]	-0.08	-1.23,1.07	0.892

age [3]	-0.04	-1.14,1.06	0.938
age [4]	0.07	-1.38,1.53	0.923
partyid	-0.17	-0.31,-0.03	**0.021**
followers	-261.92	-350.50,-173.33	**<0.001**
Observations	1307		
R^2 / R^2 adjusted	0.998 / 0.998		

Zero inflated negative binomial fit, exponentiated coefficients, without 1% greatest sharers

Stu 1 - followers likemindedness on shares of incongruent real partisan news (expon.)

	share		
Predictors	*Incidence Rate Ratios*	*CI*	*p*
**Count Model**			
(Intercept)	0.49	0.28,0.87	**0.015**
followers likemindedness	0.76	0.65,0.88	**<0.001**
female [1]	0.81	0.59,1.10	0.179
hh income	1.26	1.08,1.47	**0.004**
highered [1]	2.51	1.77,3.54	**<0.001**
age [2]	1.19	0.73,1.94	0.478
age [3]	1.64	1.02,2.65	**0.042**
age [4]	1.72	0.85,3.46	0.131
partyid	1.29	1.20,1.39	**<0.001**
followers	1.18	0.93,1.49	0.164
**Zero-Inflated Model**			
(Intercept)	0.00	0.00,0.00	**<0.001**
followers likemindedness	1.16	0.85,1.58	0.355
female [1]	0.92	0.49,1.75	0.810
hh income	1.19	0.85,1.66	0.314
highered [1]	2.00	0.99,4.03	0.054

age [2]	0.92	0.29,2.92	0.892
age [3]	0.96	0.32,2.87	0.938
age [4]	1.07	0.25,4.61	0.923
partyid	0.85	0.74,0.98	**0.021**
followers	0.00	0.00,0.00	**<0.001**
Observations	1307		
R^2 / R^2 adjusted	0.998 / 0.998		

Negative binomial fit, exponentiated coefficients

Stu 1 - followers likemindedness on shares of incongruent real partisan news (expon.)

	share		
Predictors	*Incidence Rate Ratios*	*CI*	*p*
(Intercept)	0.21	0.11,0.41	**<0.001**
followers likemindedness	0.72	0.61,0.84	**<0.001**
female [1]	0.83	0.59,1.15	0.244
white [1]	1.57	1.04,2.33	**0.026**
hh income	1.30	1.10,1.53	**0.002**
highered [1]	2.41	1.67,3.43	**<0.001**
age [2]	1.21	0.73,1.98	0.452
age [3]	1.46	0.87,2.36	0.138
age [4]	1.36	0.69,2.80	0.384
followers	2.36		**<0.001**
partyid	1.31	1.21,1.41	**<0.001**
Observations	1308		
R^2 Nagelkerke	0.284		

Poisson regression, non-exponentiated coefficients

Stu 1 - followers likemindedness on shares of incongruent real partisan news (Poisson)(log)

	share		
Predictors	*Log-Mean*	*CI*	*p*
(Intercept)	-2.37	-2.57,-2.17	**<0.001**
followers likemindedness	-0.50	-0.53,-0.47	**<0.001**
female [1]	-0.56	-0.63,-0.48	**<0.001**
white [1]	0.65	0.54,0.77	**<0.001**
hh income	0.36	0.32,0.39	**<0.001**
highered [1]	1.09	0.99,1.20	**<0.001**
age [2]	0.78	0.64,0.92	**<0.001**
age [3]	0.30	0.17,0.44	**<0.001**
age [4]	0.27	0.09,0.44	**0.003**
followers	0.03	0.01,0.04	**<0.001**
partyid	0.35	0.33,0.36	**<0.001**
Observations	1308		
R^2 Nagelkerke	0.993		

Poisson regression, exponentiated coefficients

Stu 1 - followers likemindedness on shares of incongruent real partisan news (Poisson)(expon.)

	share		
Predictors	*Incidence Rate Ratios*	*CI*	*p*
(Intercept)	0.09	0.08,0.11	**<0.001**
followers likemindedness	0.61	0.59,0.62	**<0.001**
female [1]	0.57	0.53,0.62	**<0.001**
white [1]	1.92	1.72,2.15	**<0.001**
hh income	1.43	1.38,1.48	**<0.001**
highered [1]	2.99	2.69,3.32	**<0.001**
age [2]	2.18	1.90,2.50	**<0.001**
age [3]	1.35	1.18,1.55	**<0.001**
age [4]	1.30	1.09,1.55	**0.003**
followers	1.03	1.01,1.04	**<0.001**
partyid	1.42	1.39,1.44	**<0.001**
Observations	1308		
R^2 Nagelkerke	0.993		

E.4.5 Congruent false news (main Allcott et al. 2019's classification)

Zero-inflated negative binomial, Log odds:

Stu 1 - followers likemindedness on shares of congruent false partisan news (log)

		share	
Predictors	*Log-Mean*	*CI*	*p*
**Count Model**			
(Intercept)	-4.33	-6.43,-2.23	**<0.001**
followers likemindedness	-0.75	-1.51,0.02	0.055
female [1]	1.15	0.26,2.04	**0.012**
hh income	-0.02	-0.48,0.44	0.929
highered [1]	-0.17	-1.42,1.09	0.797
age [2]	1.14	-0.97,3.26	0.290
age [3]	2.24	0.32,4.15	**0.022**
age [4]	3.80	1.58,6.01	**0.001**
followers	7.96	1.69,14.24	**0.013**
partyid	0.59	0.42,0.76	**<0.001**
**Zero-Inflated Model**			
(Intercept)	-2.03	-7.90,3.84	0.497
followers likemindedness	-1.34	-2.43,-0.25	**0.016**
female [1]	1.32	-0.28,2.92	0.106
hh income	0.27	-0.46,0.99	0.470
highered [1]	-0.49	-2.14,1.15	0.559
age [2]	2.53	-2.18,7.25	0.293
age [3]	0.72	-3.71,5.16	0.749
age [4]	1.35	-3.36,6.06	0.575
followers	0.75	-0.79,2.28	0.342

partyid	-0.06	-0.34,0.22	0.694
Observations	1308		
R^2 / R^2 adjusted	1.000 / 1.000		

Zero-inflated negative binomial, Exponentiated coefficients:

Stu 1 - followers likemindedness on shares of congruent false partisan news (expon.)

		**share**	
Predictors	*Incidence Rate Ratios*	*CI*	*p*
**Count Model**			
(Intercept)	0.01	0.00,0.11	**<0.001**
followers likemindedness	0.47	0.22,1.02	0.055
female [1]	3.15	1.29,7.66	**0.012**
hh income	0.98	0.62,1.56	0.929
highered [1]	0.85	0.24,2.98	0.797
age [2]	3.14	0.38,26.17	0.290
age [3]	9.35	1.38,63.37	**0.022**
age [4]	44.49	4.86,407.09	**0.001**
followers	2877.45	5.41,1530319.02	**0.013**
partyid	1.80	1.52,2.13	**<0.001**
**Zero-Inflated Model**			
(Intercept)	0.13	0.00,46.30	0.497
followers likemindedness	0.26	0.09,0.78	**0.016**
female [1]	3.75	0.76,18.54	0.106
hh income	1.31	0.63,2.69	0.470
highered [1]	0.61	0.12,3.17	0.559
age [2]	12.59	0.11,1407.96	0.293
age [3]	2.06	0.02,174.65	0.749
age [4]	3.85	0.03,428.14	0.575

followers	2.11	0.45,9.79	0.342
partyid	0.95	0.71,1.25	0.694
Observations	1308		
R^2 / R^2 adjusted	1.000 / 1.000		

Zero-inflated negative binomial, non-exponentiated coefficients, without 1% greater sharers:

Stu 1 - followers likemindedness on shares of congruent false partisan news (log)

	share		
Predictors	*Log-Mean*	*CI*	*p*
**Count Model**			
(Intercept)	-3.89	-7.33,-0.45	**0.027**
followers likemindedness	-0.62	-1.11,-0.12	**0.016**
female [1]	0.47	-0.34,1.27	0.256
hh income	-0.01	-0.49,0.46	0.961
highered [1]	-0.22	-1.07,0.63	0.617
age [2]	2.51	0.08,4.94	**0.043**
age [3]	2.16	-0.08,4.40	0.058
age [4]	2.83	0.27,5.38	**0.030**
followers	2.91	-1.06,6.87	0.151
partyid	0.28	0.10,0.47	**0.002**
**Zero-Inflated Model**			
(Intercept)	-0.52	-9.15,8.11	0.906
followers likemindedness	-0.97	-2.02,0.08	0.070
female [1]	0.55	-0.44,1.55	0.275
hh income	0.10	-0.46,0.66	0.725
highered [1]	-0.32	-1.36,0.73	0.553
age [2]	2.87	-4.30,10.04	0.432
age [3]	1.15	-5.01,7.31	0.714

age [4]	2.10	-4.73,8.92	0.547
followers	0.38	-1.41,2.16	0.680
partyid	-0.01	-0.24,0.23	0.954
Observations	1281		
R^2 / R^2 adjusted	1.000 / 1.000		

Zero-inflated negative binomial, Exponentiated coefficients, without 1% greater sharers:

Stu 1 - followers likemindedness on shares of congruent false partisan news (expon.)

	**share**		
Predictors	*Incidence Rate Ratios*	*CI*	*p*
**Count Model**			
(Intercept)	0.02	0.00,0.64	**0.027**
followers likemindedness	0.54	0.33,0.89	**0.016**
female [1]	1.59	0.71,3.56	0.256
hh income	0.99	0.61,1.59	0.961
highered [1]	0.80	0.34,1.89	0.617
age [2]	12.34	1.09,140.14	**0.043**
age [3]	8.67	0.93,81.10	0.058
age [4]	16.88	1.31,216.97	**0.030**
followers	18.29	0.35,962.23	0.151
partyid	1.33	1.11,1.59	**0.002**
**Zero-Inflated Model**			
(Intercept)	0.60	0.00,3343.57	0.906
followers likemindedness	0.38	0.13,1.08	0.070
female [1]	1.74	0.64,4.71	0.275
hh income	1.11	0.63,1.93	0.725
highered [1]	0.73	0.26,2.07	0.553
age [2]	17.66	0.01,22949.41	0.432

age [3]	3.17	0.01,1496.03	0.714
age [4]	8.14	0.01,7502.82	0.547
followers	1.46	0.24,8.69	0.680
partyid	0.99	0.78,1.26	0.954
Observations	1281		
R^2 / R^2 adjusted	1.000 / 1.000		

Negative binomial fit, exponentiated coefficients

Stu 1 - followers likemindedness on shares of congruent false partisan news (expon.)

	share		
Predictors	*Incidence Rate Ratios*	*CI*	*p*
(Intercept)	0.02	0.01,0.08	**<0.001**
followers likemindedness	0.98	0.70,1.38	0.917
female [1]	1.23	0.61,2.49	0.569
hh income	0.66	0.46,0.96	**0.028**
highered [1]	0.94	0.43,2.03	0.874
age [2]	0.87	0.27,2.84	0.818
age [3]	5.26	1.72,16.14	**0.004**
age [4]	31.86	7.23,140.33	**<0.001**
followers	5.64	4.17,7.65	**<0.001**
partyid	1.72	1.48,2.00	**<0.001**
Observations	1308		
R^2 Nagelkerke	0.356		

Poisson regression (log odds)

Stu 1 - followers likemindedness on shares of congruent false partisan news (log)(Poisson)

	share

Predictors	*Log-Mean*	*CI*	*p*
(Intercept)	-4.18	-4.52,-3.85	**<0.001**
followers likemindedness	0.31	0.25,0.36	**<0.001**
female [1]	0.47	0.37,0.57	**<0.001**
white [1]	-0.28	-0.40,-0.15	**<0.001**
hh income	-0.14	-0.20,-0.09	**<0.001**
highered [1]	1.51	1.35,1.66	**<0.001**
age [2]	-1.11	-1.48,-0.75	**<0.001**
age [3]	1.47	1.21,1.74	**<0.001**
age [4]	2.96	2.70,3.24	**<0.001**
followers	0.10	0.07,0.12	**<0.001**
partyid	0.46	0.44,0.49	**<0.001**
Observations	1308		
R^2 Nagelkerke	0.971		

Poisson (exponentiated)

Stu 1 - followers likemindedness on shares of congruent false partisan news (expon.)(Poisson)

		share	
Predictors	*Incidence Rate Ratios*	*CI*	*p*
(Intercept)	0.02	0.01,0.02	**<0.001**
followers likemindedness	1.36	1.29,1.43	**<0.001**
female [1]	1.60	1.45,1.77	**<0.001**
white [1]	0.76	0.67,0.86	**<0.001**
hh income	0.87	0.82,0.92	**<0.001**
highered [1]	4.50	3.87,5.27	**<0.001**
age [2]	0.33	0.23,0.47	**<0.001**
age [3]	4.33	3.35,5.72	**<0.001**
age [4]	19.24	14.84,25.45	**<0.001**

followers	1.11	1.08,1.13	**<0.001**
partyid	1.59	1.55,1.62	**<0.001**
Observations	1308		
R^2 Nagelkerke	0.971		

E.4.6 Congruent false news (Grindberg's alternative classification)

Zero-inflated negative binomial fit, non-exponentiated coefficients

Stu 1 - followers likemindedness on shares of congruent false partisan news (log)

	share		
Predictors	*Log-Mean*	*CI*	*p*
**Count Model**			
(Intercept)	-0.13	-4.32,4.06	0.952
followers likemindedness	0.53	0.06,0.99	**0.026**
female [1]	0.41	-0.70,1.53	0.468
white [1]	-0.23	-1.26,0.80	0.662
hh income	-0.93	-1.32,-0.53	**<0.001**
highered [1]	1.03	0.06,1.99	**0.037**
age [2]	-2.64	-6.25,0.98	0.153
age [3]	-1.38	-4.86,2.10	0.437
age [4]	-1.64	-5.27,1.98	0.373
followers	7.88	0.42,15.33	**0.038**
partyid	0.35	0.06,0.65	**0.019**
**Zero-Inflated Model**			
(Intercept)	7.75	2.71,12.78	**0.003**
followers likemindedness	0.09	-0.70,0.88	0.820
female [1]	0.73	-0.84,2.30	0.360
hh income	-0.40	-1.32,0.53	0.403
highered [1]	-0.05	-1.59,1.48	0.946
age [2]	-4.43	-8.34,-0.52	**0.026**
age [3]	-6.36	-11.19,-1.53	**0.010**
age [4]	-5.86	-11.29,-0.42	**0.035**
followers	2.19	-1.45,5.84	0.238

partyid	-0.95	-1.63,-0.27	**0.006**
Observations	1308		
R^2 / R^2 adjusted	1.000 / 1.000		

Zero-inflated negative binomial fit, exponentiated coefficients

Stu 1 - followers likemindedness on shares of congruent false partisan news (expon.)

	share		
Predictors	*Incidence Rate Ratios*	*CI*	*p*
**Count Model**			
(Intercept)	0.88	0.01,57.83	0.952
followers likemindedness	1.70	1.07,2.70	**0.026**
female [1]	1.51	0.49,4.63	0.468
white [1]	0.79	0.28,2.23	0.662
hh income	0.40	0.27,0.59	**<0.001**
highered [1]	2.79	1.06,7.31	**0.037**
age [2]	0.07	0.00,2.66	0.153
age [3]	0.25	0.01,8.17	0.437
age [4]	0.19	0.01,7.22	0.373
followers	2631.94	1.52,4554494.10	**0.038**
partyid	1.42	1.06,1.91	**0.019**
**Zero-Inflated Model**			
(Intercept)	2311.45	15.09,354155.08	**0.003**
followers likemindedness	1.10	0.50,2.41	0.820
female [1]	2.08	0.43,9.98	0.360
hh income	0.67	0.27,1.70	0.403
highered [1]	0.95	0.20,4.40	0.946
age [2]	0.01	0.00,0.59	**0.026**

age [3]	0.00	0.00,0.22	**0.010**
age [4]	0.00	0.00,0.66	**0.035**
followers	8.96	0.23,342.69	0.238
partyid	0.39	0.20,0.76	**0.006**
Observations	1308		
R^2 / R^2 adjusted	1.000 / 1.000		

Zero-inflated negative binomial fit, non-exponentiated coefficients, without 1% greatest sharers

Stu 1 - followers likemindedness on shares of congruent false partisan news (log)

	share		
Predictors	*Log-Mean*	*CI*	*p*
**Count Model**			
(Intercept)	-5.68	-8.84,-2.52	**<0.001**
followers likemindedness	0.61	0.17,1.05	**0.006**
female [1]	-0.11	-0.84,0.62	0.761
white [1]	-0.61	-1.41,0.18	0.131
hh income	0.28	-0.16,0.72	0.208
highered [1]	0.46	-0.38,1.30	0.279
age [2]	3.56	0.80,6.32	**0.012**
age [3]	4.23	1.49,6.97	**0.002**
age [4]	4.28	1.29,7.26	**0.005**
followers	-3.59	-6.55,-0.63	**0.017**
partyid	0.22	0.05,0.40	**0.012**
**Zero-Inflated Model**			
(Intercept)	-3.54	-11.25,4.17	0.368
followers likemindedness	0.41	-0.40,1.22	0.320
female [1]	0.42	-0.82,1.67	0.504

hh income	0.75	-0.02,1.51	0.057
highered [1]	0.35	-0.99,1.69	0.606
age [2]	2.10	-3.62,7.82	0.471
age [3]	1.31	-4.46,7.09	0.656
age [4]	1.93	-3.95,7.81	0.520
followers	-39.11	-71.20,-7.01	**0.017**
partyid	-0.40	-0.65,-0.15	**0.001**
Observations	1282		
R^2 / R^2 adjusted	0.055 / 0.047		

Zero-inflated negative binomial fit, exponentiated coefficients, without 1% greatest sharers

Stu 1 - followers likemindedness on shares of congruent false partisan news (expon.)

	share		
Predictors	*Incidence Rate Ratios*	*CI*	*p*
**Count Model**			
(Intercept)	0.00	0.00,0.08	**<0.001**
followers likemindedness	1.84	1.19,2.85	**0.006**
female [1]	0.89	0.43,1.85	0.761
white [1]	0.54	0.24,1.20	0.131
hh income	1.32	0.86,2.05	0.208
highered [1]	1.59	0.69,3.67	0.279
age [2]	35.14	2.22,556.13	**0.012**
age [3]	68.64	4.45,1059.41	**0.002**
age [4]	72.05	3.65,1422.34	**0.005**
followers	0.03	0.00,0.53	**0.017**
partyid	1.25	1.05,1.49	**0.012**
**Zero-Inflated Model**			
(Intercept)	0.03	0.00,64.54	0.368

followers likemindedness	1.51	0.67,3.38	0.320
female [1]	1.53	0.44,5.30	0.504
hh income	2.11	0.98,4.54	0.057
highered [1]	1.42	0.37,5.41	0.606
age [2]	8.19	0.03,2491.08	0.471
age [3]	3.71	0.01,1196.20	0.656
age [4]	6.90	0.02,2469.09	0.520
followers	0.00	0.00,0.00	**0.017**
partyid	0.67	0.52,0.86	**0.001**
Observations	1282		
R^2 / R^2 adjusted	0.055 / 0.047		

Negative binomial fit, exponentiated coefficients

Stu 1 - followers likemindedness on shares of congruent false partisan news (expon.)

	share		
Predictors	*Incidence Rate Ratios*	*CI*	*p*
(Intercept)	0.00	0.00,0.01	**<0.001**
followers likemindedness	1.29	0.93,1.81	0.128
female [1]	1.24	0.62,2.49	0.542
white [1]	1.42	0.61,3.31	0.413
hh income	0.55	0.38,0.80	**0.001**
highered [1]	2.19	1.00,4.78	**0.049**
age [2]	2.12	0.54,8.40	0.284
age [3]	12.13	3.21,45.82	**<0.001**
age [4]	8.63	1.64,45.56	**0.011**
followers	1.12	0.85,1.48	0.428
partyid	2.21	1.91,2.57	**<0.001**
Observations	1308		
R^2 Nagelkerke	0.461		

Poisson (non-exponentiated)

Stu 1 - followers likemindedness on shares of congruent false partisan news (log)(Poisson)

	share		
Predictors	*Log-Mean*	*CI*	*p*
(Intercept)	-5.37	-5.96,-4.86	**<0.001**
followers likemindedness	0.28	0.23,0.33	**<0.001**
female [1]	0.65	0.54,0.75	**<0.001**
white [1]	-0.96	-1.07,-0.85	**<0.001**
hh income	-0.55	-0.62,-0.49	**<0.001**
highered [1]	1.34	1.21,1.48	**<0.001**
age [2]	1.12	0.62,1.70	**<0.001**
age [3]	3.32	2.85,3.88	**<0.001**
age [4]	3.19	2.69,3.76	**<0.001**
followers	0.10	0.07,0.12	**<0.001**
partyid	0.48	0.46,0.51	**<0.001**
Observations	1308		
R^2 Nagelkerke	0.957		

Poisson (exponentiated)

Stu 1 - followers likemindedness on shares of congruent false partisan news (expon.)(Poisson)

	share		
Predictors	*Incidence Rate Ratios*	*CI*	*p*
(Intercept)	0.00	0.00,0.01	**<0.001**
followers likemindedness	1.32	1.25,1.40	**<0.001**
female [1]	1.91	1.72,2.11	**<0.001**

white [1]	0.38	0.34,0.43	**<0.001**
hh income	0.57	0.54,0.61	**<0.001**
highered [1]	3.82	3.35,4.38	**<0.001**
age [2]	3.07	1.86,5.47	**<0.001**
age [3]	27.75	17.31,48.41	**<0.001**
age [4]	24.20	14.77,42.87	**<0.001**
followers	1.10	1.07,1.12	**<0.001**
partyid	1.62	1.58,1.66	**<0.001**
Observations	1308		
R^2 Nagelkerke	0.957		

E.4.7 Incongruent false news (main Allcott et al. 2019's classification)

Zero-inflated negative binomial, Log odds:

Stu 1 - followers likemindedness on shares of incongruent false partisan news (log)

	share		
Predictors	*Log-Mean*	*CI*	*p*
**Count Model**			
(Intercept)	-3.76	-8.30,0.79	0.105
followers likemindedness	-0.18	-0.68,0.32	0.477
female [1]	-2.02	-3.61,-0.43	**0.013**
hh income	-0.25	-0.68,0.18	0.254
highered [1]	-1.32	-2.91,0.26	0.102
age [2]	4.76	0.39,9.13	**0.033**
age [3]	4.36	0.07,8.65	**0.046**
age [4]	0.26	-4.37,4.88	0.913
followers	-0.10	-0.22,0.03	0.126
partyid	0.32	-0.01,0.64	0.054
**Zero-Inflated Model**			
(Intercept)	0.92	-5.45,7.29	0.777
followers likemindedness	0.23	-0.33,0.79	0.419
female [1]	-1.94	-3.95,0.06	0.057
hh income	0.16	-0.46,0.79	0.615
highered [1]	-1.65	-3.77,0.46	0.126
age [2]	4.56	-1.65,10.76	0.150
age [3]	2.75	-3.35,8.84	0.377
age [4]	-3.25	-16.23,9.72	0.623
followers	-0.97	-1.69,-0.24	**0.009**
partyid	0.33	-0.03,0.68	0.069

Observations	1308
R^2 / R^2 adjusted	0.005 / -0.002

Zero-inflated negative binomial, Exponentiated coefficients:

Stu 1 - followers likemindedness on shares of incongruent false partisan news (expon.)

	share		
Predictors	*Incidence Rate Ratios*	*CI*	*p*
**Count Model**			
(Intercept)	0.02	0.00,2.20	0.105
followers likemindedness	0.83	0.50,1.38	0.477
female [1]	0.13	0.03,0.65	**0.013**
hh income	0.78	0.51,1.20	0.254
highered [1]	0.27	0.05,1.30	0.102
age [2]	116.52	1.48,9204.47	**0.033**
age [3]	78.32	1.08,5703.27	**0.046**
age [4]	1.29	0.01,131.73	0.913
followers	0.91	0.80,1.03	0.126
partyid	1.38	0.99,1.91	0.054
**Zero-Inflated Model**			
(Intercept)	2.51	0.00,1467.51	0.777
followers likemindedness	1.26	0.72,2.20	0.419
female [1]	0.14	0.02,1.06	0.057
hh income	1.17	0.63,2.19	0.615
highered [1]	0.19	0.02,1.59	0.126
age [2]	95.46	0.19,47270.27	0.150
age [3]	15.57	0.04,6887.13	0.377
age [4]	0.04	0.00,16707.15	0.623

followers	0.38	0.18,0.79	**0.009**
partyid	1.39	0.98,1.97	0.069
Observations	1308		
R^2 / R^2 adjusted	0.005 / -0.002		

Zero-inflated negative binomial, Exponentiated coefficients, without 1% greater sharers:

Stu 1 - followers likemindedness on shares of incongruent false partisan news (expon.)

	share		
Predictors	*Incidence Rate Ratios*	*CI*	*p*
**Count Model**			
(Intercept)	0.02	0.00,2.20	0.105
followers likemindedness	0.83	0.50,1.38	0.477
female [1]	0.13	0.03,0.65	**0.013**
hh income	0.78	0.51,1.20	0.254
highered [1]	0.27	0.05,1.30	0.102
age [2]	116.52	1.48,9204.47	**0.033**
age [3]	78.32	1.08,5703.27	**0.046**
age [4]	1.29	0.01,131.73	0.913
followers	0.91	0.80,1.03	0.126
partyid	1.38	0.99,1.91	0.054
**Zero-Inflated Model**			
(Intercept)	2.51	0.00,1467.51	0.777
followers likemindedness	1.26	0.72,2.20	0.419
female [1]	0.14	0.02,1.06	0.057
hh income	1.17	0.63,2.19	0.615
highered [1]	0.19	0.02,1.59	0.126
age [2]	95.46	0.19,47270.27	0.150

age [3]	15.57	0.04,6887.13	0.377
age [4]	0.04	0.00,16707.15	0.623
followers	0.38	0.18,0.79	**0.009**
partyid	1.39	0.98,1.97	0.069
Observations	1308		
R^2 / R^2 adjusted	0.005 / -0.002		

Negative binomial fit, exponentiated coefficients

Stu 1 - followers likemindedness on shares of incongruent false partisan news (expon.)

	share		
Predictors	*Incidence Rate Ratios*	*CI*	*p*
(Intercept)	0.01	0.00,0.06	**<0.001**
followers likemindedness	0.68	0.39,1.13	0.112
female [1]	0.94	0.32,2.82	0.910
white [1]	0.51	0.14,1.70	0.265
hh income	0.78	0.47,1.27	0.360
highered [1]	1.68	0.54,5.57	0.379
age [2]	2.36	0.28,52.16	0.483
age [3]	11.39	1.53,243.35	**0.037**
age [4]	3.82	0.23,114.47	0.359
followers	2.06		**<0.001**
partyid	0.96	0.72,1.24	0.697
Observations	1308		
R^2 Nagelkerke	0.186		

Poisson regression (log odds)

Stu 1 - followers likemindedness on shares of incongruent false partisan news (log)

		share	
Predictors	*Log-Mean*	*CI*	*p*
(Intercept)	-5.32	-8.29,-3.54	**<0.001**
followers likemindedness	-0.27	-0.55,0.01	0.060
female [1]	-0.16	-0.84,0.50	0.635
white [1]	-0.55	-1.24,0.20	0.129
hh income	-0.33	-0.68,0.01	0.058
highered [1]	0.52	-0.21,1.31	0.176
age [2]	0.96	-0.81,3.91	0.373
age [3]	2.43	0.88,5.32	**0.017**
age [4]	1.38	-0.97,4.46	0.260
followers	0.09	-0.06,0.17	0.121
partyid	0.01	-0.14,0.15	0.885
Observations	1308		
R^2 Nagelkerke	0.124		

Poisson regression (expon.)

Stu 1 - followers likemindedness on shares of incongruent false partisan news (expon.)

		share	
Predictors	*Incidence Rate Ratios*	*CI*	*p*
(Intercept)	0.00	0.00,0.03	**<0.001**
followers likemindedness	0.76	0.58,1.01	0.060
female [1]	0.85	0.43,1.64	0.635
white [1]	0.58	0.29,1.22	0.129
hh income	0.72	0.50,1.01	0.058
highered [1]	1.68	0.81,3.71	0.176
age [2]	2.62	0.44,49.71	0.373
age [3]	11.42	2.41,204.29	**0.017**
age [4]	3.99	0.38,86.22	0.260

followers		1.09	0.94,1.19	0.121
partyid		1.01	0.87,1.16	0.885
Observations	1308			
R^2 Nagelkerke	0.124			

E.4.8 Incongruent false news (Grindberg's alternative classification)

Zero-inflated negative binomial fit, non-exponentiated coefficients

Stu 1 - followers likemindedness on shares of incongruent false partisan news (log)

	share		
Predictors	*Log-Mean*	*CI*	*p*
**Count Model**			
(Intercept)	-4.44	-7.01,-1.87	**0.001**
followers likemindedness	-0.57	-1.06,-0.09	**0.021**
female [1]	-0.84	-1.93,0.25	0.131
white [1]	-0.09	-1.26,1.08	0.880
hh income	0.47	-0.12,1.05	0.119
highered [1]	-0.13	-1.18,0.93	0.815
age [2]	-0.33	-2.80,2.14	0.793
age [3]	1.45	-0.89,3.80	0.225
age [4]	1.10	-1.88,4.08	0.469
followers	-1.16	-3.87,1.55	0.401
partyid	0.72	0.32,1.12	**<0.001**
**Zero-Inflated Model**			
(Intercept)	-141.68	-639.68,356.33	0.577
followers likemindedness	-79.34	-294.39,135.71	0.470
female [1]	-310.48	-1111.07,490.10	0.447
hh income	199.65	-282.12,681.42	0.417
highered [1]	47.53	-59.98,155.04	0.386
age [2]	-452.35	-1684.70,780.01	0.472
age [3]	-361.93	-1407.81,683.94	0.498
age [4]	6.85	-2072.28,2085.97	0.995
followers	-624.13	-2180.42,932.16	0.432

partyid	145.77	-215.03,506.58	0.428
Observations	1308		
R^2 / R^2 adjusted	0.032 / 0.024		

Zero-inflated negative binomial fit, exponentiated coefficients

**Stu 1 - followers likemindedness on shares of incongruent false partisan news (expon.)**				
	**share**			
Predictors	*Incidence Rate Ratios*	*CI*	*p*	
**Count Model**				
(Intercept)	0.01	0.00,0.15	**0.001**	
followers likemindedness	0.56	0.35,0.92	**0.021**	
female [1]	0.43	0.15,1.28	0.131	
white [1]	0.91	0.28,2.96	0.880	
hh income	1.59	0.89,2.87	0.119	
highered [1]	0.88	0.31,2.53	0.815	
age [2]	0.72	0.06,8.48	0.793	
age [3]	4.28	0.41,44.75	0.225	
age [4]	3.00	0.15,58.93	0.469	
followers	0.31	0.02,4.70	0.401	
partyid	2.05	1.37,3.08	**<0.001**	
**Zero-Inflated Model**				
(Intercept)	0.00	0.00,56347372590341573980301856347024042160063967367165463841754964751520421969471665630627283426795312098065010859036092893771770995	0.577	

		65853545371 68393210072 92416.00		
followers likemindedness	0.00	0.00,8635919 36305829238 94205346337 28085337486 85539665987 20847872.00	0.470	
female [1]	0.00	0.00,7070377 00955538554 23043816401 96476234751 38149067179 81302147765 59104993034 92706856321 55692604402 25800081986 28532288920 20591993414 04559020592 37654699044 09553611769 89266941805 53012585738 49796874043 68771891469 90346240.00	0.447	
hh income	51008504587 80582665974 14498196004 93815864718 67142373026 81426224989 10024312661 5441145856.0 0	0.00,8656465 16398719774 71515312637 93681816551 91535603897 14991363362 50306226219 87362426670 35035822217 72994145114 77211416502 40634661678 01181130076 45571429747 13308204472 48731129680 25156772773	0.417	

		23985608629<br>65706658192<br>53600883359<br>49159373405<br>88041847317<br>62580994636<br>41371534401<br>10508757400<br>06173537520<br>69297827479<br>552.00		
highered [1]	43838630832<br>7821541376.0<br>0	0.00,2144790<br>64157522239<br>99409946615<br>52990816171<br>59485893134<br>17311566813<br>986816.00	0.386	
age [2]	0.00	0.00,Inf	0.472	
age [3]	0.00	0.00,1073934<br>36751119063<br>89599102839<br>63351854811<br>91371935793<br>26028715614<br>58402780174<br>75683518593<br>36744249215<br>41398898494<br>91765135396<br>10774000224<br>94265408309<br>87642311545<br>26236501858<br>26926614278<br>81319375320<br>64900407815<br>48611234871<br>40501824331<br>85510387846<br>12198735806<br>59259201715<br>95114238328<br>32399363512<br>49626942803	0.498	

		63067094325 00224.00		
age [4]	939.68	0.00,Inf	0.995	
followers	0.00	0.00,Inf	0.432	
partyid	20379549563 82291423387 59247890425 11038249437 47375413587 383156736.00	0.00,1015396 27441550609 11618894017 87519992740 61298219533 89416274409 71021719159 90561231243 59023389710 85857973583 07134311058 29404724289 10017559684 39401326484 08382121468 09351697738 36006956887 39768857717 45145524699 64753872512 61440.00	0.428	
Observations	1308			
R^2 / R^2 adjusted	0.032 / 0.024			

Zero-inflated negative binomial fit, non-exponentiated coefficients, without 1% greatest sharers

Stu 1 - followers likemindedness on shares of incongruent false partisan news (log)

	share		
Predictors	*Log-Mean*	*CI*	*p*
**Count Model**			
(Intercept)	-4.65	-7.13,-2.17	**<0.001**
followers likemindedness	-0.63	-1.15,-0.10	**0.019**
female [1]	-0.60	-1.67,0.46	0.266
white [1]	0.02	-1.18,1.23	0.972

hh income	0.51	-0.08,1.09	0.090
highered [1]	-0.22	-1.27,0.82	0.677
age [2]	-0.10	-2.51,2.32	0.938
age [3]	1.64	-0.65,3.93	0.161
age [4]	1.15	-1.76,4.06	0.440
followers	-1.73	-4.25,0.79	0.179
partyid	0.61	0.24,0.97	**0.001**
**Zero-Inflated Model**			
(Intercept)	-202.39	-631.63,226.85	0.355
followers likemindedness	-121.85	-378.30,134.61	0.352
female [1]	-210.86	-654.45,232.73	0.352
hh income	154.95	-173.36,483.25	0.355
highered [1]	95.86	-106.22,297.94	0.353
age [2]	-377.13	-1178.80,424.54	0.357
age [3]	-264.00	-823.91,295.91	0.355
age [4]	-1.95	-59.93,56.03	0.947
followers	-404.36	-1258.86,450.15	0.354
partyid	125.55	-140.42,391.52	0.355
Observations	1306		
R^2 / R^2 adjusted	0.011 / 0.003		

Zero-inflated negative binomial fit, exponentiated coefficients, without 1% greatest sharers

**Stu 1 - followers likemindedness on shares of incongruent false partisan news (expon.)**				
	**share**			
Predictors	*Incidence Rate Ratios*	*CI*	*p*	

**Count Model**				
(Intercept)	0.01	0.00,0.11	**<0.001**	
followers likemindedness	0.53	0.32,0.90	**0.019**	
female [1]	0.55	0.19,1.59	0.266	
white [1]	1.02	0.31,3.41	0.972	
hh income	1.66	0.92,2.97	0.090	
highered [1]	0.80	0.28,2.28	0.677	
age [2]	0.91	0.08,10.15	0.938	
age [3]	5.13	0.52,50.68	0.161	
age [4]	3.15	0.17,57.86	0.440	
followers	0.18	0.01,2.21	0.179	
partyid	1.83	1.27,2.65	**0.001**	
**Zero-Inflated Model**				
(Intercept)	0.00	0.00,3300570718848250428425920748005797709723609263688813295801303908166134026946854379294038325188 36224.00	0.355	
followers likemindedness	0.00	0.00,287296254567134651554249583959170562716889543372855 96250112.00	0.352	
female [1]	0.00	0.00,1181604418893724583089256784262020423080852144680730604610637137683026945347879925571685787322 49579520.00	0.352	
hh income	19582638111115056598532181607160506308408263	0.00,7498321466341906778060675608134627357478	0.355	

	0551895233934396447784 96.00	2576528803480007384968359085976138753587994030167105219524296654773910990276961189468331720616943100703082471041915457419883498562099077120343527996272074926544229551 3088.00		
highered [1]	427971805511098574307104798272252956114944.00	0.00,24747299640634265829829671928611908218359500108454511107773998778548646062082374343392698904867928010974495388602048615832008785 92.00	0.353	
age [2]	0.00	0.00,2378730999759504262722976214624637597419985841030116649752633909972096282662629676520079944197441813282455009740862021081122318919629790245288677174243093560199803341056014	0.357	

		3081315762176.00		
age [3]	0.00	0.00,32636128520184518789046037624319528524266879069231401306875765252529505076707208692231317114485184819019019328869613978909251993 6.00	0.355	
age [4]	0.14	0.00,21563463215080392401879 04.00	0.947	
followers	0.00	0.00,31404852445857256528871984012868634107432801026601106696609550520445162616842449142010669548660409816877937660187369439779091328138818168941157863002055850524687845325877739141441560081968534650 88.00	0.354	
partyid	3357838981887591847143606124448920064252192896722141184.00	0.00,10806317061410225407167103094310148516568234656309072797183848492631088192128931916418384364645	0.355	

		34408072789 68313981419 51358748725 32663757515 74049842736 71515043421 0211168256.0 0		
Observations	1306			
R^2 / R^2 adjusted	0.011 / 0.003			

Negative binomial fit, exponentiated coefficients

Stu 1 - followers likemindedness on shares of incongruent false partisan news (expon.)

	share		
Predictors	*Incidence Rate Ratios*	*CI*	*p*
(Intercept)	0.01	0.00,0.09	**<0.001**
followers likemindedness	0.76	0.46,1.27	0.296
female [1]	1.36	0.47,3.89	0.568
white [1]	0.88	0.24,3.22	0.843
hh income	0.70	0.40,1.23	0.218
highered [1]	0.97	0.31,3.09	0.963
age [2]	3.29	0.31,35.22	0.324
age [3]	20.75	2.10,205.14	**0.009**
age [4]	4.09	0.21,77.91	0.349
followers	1.06	0.69,1.63	0.792
partyid	0.90	0.71,1.14	0.373
Observations	1308		
R^2 Nagelkerke	0.204		

Poisson regression (non-exponentiated)

Stu 1 - followers likemindedness on shares of incongruent false partisan news (log)(Poisson)

	share		
Predictors	*Log-Mean*	*CI*	*p*
(Intercept)	-5.75	-8.68,-4.07	**<0.001**
followers likemindedness	-0.45	-0.67,-0.24	**<0.001**
female [1]	0.26	-0.23,0.75	0.294
white [1]	-0.38	-0.92,0.20	0.174
hh income	-0.51	-0.78,-0.24	**<0.001**
highered [1]	0.46	-0.08,1.02	0.098
age [2]	1.47	-0.20,4.39	0.162
age [3]	3.16	1.64,6.03	**0.002**
age [4]	1.47	-0.88,4.54	0.232
followers	-0.05	-0.38,0.10	0.629
partyid	-0.00	-0.12,0.10	0.932
Observations	1308		
R^2 Nagelkerke	0.172		

Poisson regression (exponentiated)

Stu 1 - followers likemindedness on shares of incongruent false partisan news (expon.)(Poisson)

	share		
Predictors	*Incidence Rate Ratios*	*CI*	*p*
(Intercept)	0.00	0.00,0.02	**<0.001**
followers likemindedness	0.63	0.51,0.79	**<0.001**
female [1]	1.30	0.80,2.12	0.294
white [1]	0.68	0.40,1.22	0.174
hh income	0.60	0.46,0.78	**<0.001**
highered [1]	1.58	0.93,2.76	0.098

age [2]		4.37	0.82,80.67	0.162
age [3]		23.54	5.13,417.64	**0.002**
age [4]		4.33	0.41,93.48	0.232
followers		0.95	0.68,1.10	0.629
partyid		1.00	0.89,1.11	0.932
Observations	1308			
R^2 Nagelkerke	0.172			

E.5 Regressions: associations between political slant of the followers and shares from domains congenial to the followers

As our last set of analyses in Study 1, we examined the associations between the political slant of the followers and Twitter users' propensity to share news from domains which are politically congenial to those followers (irrespective of whether the domain is politically congruent to the user or not). This metric takes high values when, for instance, a user shares a story from a pro-Democrat domain to pro-Democrat followers, whether the story is congruent to the user (she is Democrat) or incongruent (she is Republican). The metric takes low values when, for instance, the user shares a story from a pro-Democrat domain to pro-Republican followers, whether the story is congruent to the user (she is Democrat) or incongruent (she is Republican). We ran this analysis, which ensures higher power, on news shared from real and false news domains separately. Because it involves stacking shares of news congruent and incongruent to each respondent together in the data (entailing repeated observations), clustering robust standard errors around participants' IDs was required.

E.5.1 Shares from real news domains (main Allsides' classification)

Zero-inflated negative binomial regression (non-exponentiated and exponentiated 'e' coefficients)

A tibble: 21 × 11

	term	estimate	std.error	statistic	p.value	conf.low	conf.high	truth_subset	estimate_e	conf.low_e	conf.high_e
	<chr>	<dbl>	<dbl>	<dbl>	<dbl>	<dbl>	<dbl>	<chr>	<dbl>	<dbl>	<dbl>
1	count_(Intercept)	1.65	0.380	4.34	1.46e- 5	0.905	2.40	real	5.21e+ 0	2.47e+ 0	1.10e+ 1
2	count_source_netw_congen	1.25	0.0680	18.4	4.46e-71	1.12	1.38	real	3.49e+ 0	3.06e+ 0	3.99e+ 0
3	count_female1	-0.121	0.198	-0.612	5.40e- 1	-0.509	0.267	real	8.86e- 1	6.01e- 1	1.31e+ 0
4	count_white1	-0.0237	0.207	-0.115	9.09e- 1	-0.429	0.381	real	9.77e- 1	6.51e- 1	1.46e+ 0
5	count_hh_income	0.167	0.0993	1.68	9.34e- 2	-0.0280	0.361	real	1.18e+ 0	9.72e- 1	1.44e+ 0
6	count_highered1	0.220	0.257	0.856	3.92e- 1	-0.284	0.723	real	1.25e+ 0	7.53e- 1	2.06e+ 0
7	count_age2	0.264	0.361	0.730	4.65e- 1	-0.444	0.971	real	1.30e+ 0	6.41e- 1	2.64e+ 0
8	count_age3	0.614	0.324	1.90	5.81e- 2	-0.0210	1.25	real	1.85e+ 0	9.79e- 1	3.49e+ 0
9	count_age4	0.792	0.455	1.74	8.19e- 2	-0.100	1.68	real	2.21e+ 0	9.05e- 1	5.39e+ 0
10	count_partyid	0.135	0.0418	3.23	1.25e- 3	0.0530	0.217	real	1.14e+ 0	1.05e+ 0	1.24e+ 0
11	count_followers	0.216	0.183	1.18	2.37e- 1	-0.143	0.575	real	1.24e+ 0	8.67e- 1	1.78e+ 0
12	zero_(Intercept)	-55.1	13.1	-4.20	2.77e- 5	-80.8	-29.4	real	1.18e- 24	7.91e- 36	1.76e- 13
13	zero_followers_likemindedness	0.0329	0.120	0.275	7.83e- 1	-0.202	0.267	real	1.03e+ 0	8.17e- 1	1.31e+ 0
14	zero_female1	0.341	0.246	1.38	1.66e- 1	-0.142	0.824	real	1.41e+ 0	8.68e- 1	2.28e+ 0
15	zero_hh_income	0.294	0.132	2.23	2.56e- 2	0.0360	0.553	real	1.34e+ 0	1.04e+ 0	1.74e+ 0
16	zero_highered1	0.0751	0.264	0.285	7.76e- 1	-0.442	0.592	real	1.08e+ 0	6.43e- 1	1.81e+ 0
17	zero_age2	-0.386	0.451	-0.856	3.92e- 1	-1.27	0.499	real	6.80e- 1	2.80e- 1	1.65e+ 0
18	zero_age3	-0.829	0.419	-1.98	4.81e- 2	-1.65	-0.00697	real	4.36e- 1	1.92e- 1	9.93e- 1
19	zero_age4	-1.30	0.524	-2.48	1.32e- 2	-2.33	-0.273	real	2.73e- 1	9.76e- 2	7.61e- 1
20	zero_partyid	0.0160	0.0537	0.299	7.65e- 1	-0.0892	0.121	real	1.02e+ 0	9.15e- 1	1.13e+ 0
21	zero_followers	-459.	106.	-4.32	1.59e- 5	-667.	-251.	real	7.00e-200	3.34e-290	1.47e-109

Zero-inflated negative binomial regression, without 1% greatest sharers (non-exponentiated and exponentiated 'e' coefficients)

A tibble: 21 × 11

	term	estimate	std.error	statistic	p.value	conf.low	conf.high	truth_subset	estimate_e	conf.low_e	conf.high_e
	<chr>	<dbl>	<dbl>	<dbl>	<dbl>	<dbl>	<dbl>	<chr>	<dbl>	<dbl>	<dbl>
1	count_(Intercept)	2.39	0.297	8.05	1.28e-15	1.81	2.97	real	10.9	6.09	19.5
2	count_source_netw_congen	0.530	0.0549	9.65	1.09e-21	0.422	0.637	real	1.70	1.53	1.89
3	count_female1	0.00187	0.130	0.0144	9.88e- 1	-0.252	0.256	real	1.00	0.777	1.29
4	count_white1	0.112	0.143	0.782	4.34e- 1	-0.168	0.391	real	1.12	0.845	1.48
5	count_hh_income	-0.0232	0.0630	-0.368	7.13e- 1	-0.147	0.100	real	0.977	0.863	1.11
6	count_highered1	0.237	0.159	1.49	1.35e- 1	-0.0743	0.549	real	1.27	0.928	1.73
7	count_age2	0.0447	0.250	0.179	8.58e- 1	-0.445	0.534	real	1.05	0.641	1.71
8	count_age3	0.364	0.242	1.50	1.33e- 1	-0.111	0.839	real	1.44	0.895	2.31
9	count_age4	0.369	0.305	1.21	2.26e- 1	-0.229	0.968	real	1.45	0.795	2.63
10	count_partyid	-0.00855	0.0307	-0.279	7.80e- 1	-0.0687	0.0516	real	0.991	0.934	1.05
11	count_followers	0.0319	0.0372	0.857	3.91e- 1	-0.0410	0.105	real	1.03	0.960	1.11
12	zero_(Intercept)	0.878	0.166	5.29	1.34e- 7	0.552	1.20	real	2.41	1.74	3.33
13	zero_followers_likemindedness	-0.0869	0.0482	-1.80	7.17e- 2	-0.181	0.00767	real	0.917	0.834	1.01
14	zero_female1	0.0432	0.0940	0.460	6.46e- 1	-0.141	0.227	real	1.04	0.868	1.26
15	zero_hh_income	-0.0697	0.0491	-1.42	1.56e- 1	-0.166	0.0267	real	0.933	0.847	1.03
16	zero_highered1	-0.216	0.108	-2.00	4.57e- 2	-0.428	-0.00409	real	0.806	0.652	0.996
17	zero_age2	-0.303	0.143	-2.12	3.37e- 2	-0.583	-0.0233	real	0.738	0.558	0.977
18	zero_age3	-0.606	0.141	-4.29	1.89e- 5	-0.883	-0.328	real	0.546	0.414	0.720
19	zero_age4	-0.547	0.194	-2.82	4.89e- 3	-0.927	-0.166	real	0.579	0.396	0.847
20	zero_partyid	0.00656	0.0220	0.298	7.66e- 1	-0.0366	0.0497	real	1.01	0.964	1.05
21	zero_followers	-0.252	0.346	-0.727	4.67e- 1	-0.931	0.427	real	0.778	0.394	1.53

Poisson regression (non-exponentiated and exponentiated 'e' coefficients)

A tibble: 11 × 11

	term	estimate	std.error	statistic	p.value	conf.low	conf.high	truth_subset	estimate_e	conf.low_e	conf.high_e
	<chr>	<dbl>	<dbl>	<dbl>	<dbl>	<dbl>	<dbl>	<chr>	<dbl>	<dbl>	<dbl>
1	(Intercept)	1.64	0.415	3.96	7.44e- 5	0.830	2.46	real	5.17	2.29	11.7
2	source_netw_congen	0.948	0.0747	12.7	6.54e-37	0.802	1.09	real	2.58	2.23	2.99
3	female1	-0.0983	0.217	-0.453	6.50e- 1	-0.523	0.327	real	0.906	0.593	1.39
4	white1	0.133	0.269	0.494	6.21e- 1	-0.394	0.659	real	1.14	0.675	1.93
5	hh_income	0.107	0.0991	1.08	2.80e- 1	-0.0872	0.301	real	1.11	0.917	1.35
6	highered1	0.323	0.276	1.17	2.41e- 1	-0.217	0.863	real	1.38	0.805	2.37
7	age2	0.179	0.346	0.516	6.06e- 1	-0.500	0.857	real	1.20	0.607	2.36
8	age3	0.659	0.326	2.02	4.30e- 2	0.0209	1.30	real	1.93	1.02	3.66
9	age4	0.964	0.487	1.98	4.75e- 2	0.0105	1.92	real	2.62	1.01	6.81
10	partyid	-0.00691	0.0435	-0.159	8.74e- 1	-0.0922	0.0783	real	0.993	0.912	1.08
11	followers	0.0466	0.0306	1.52	1.28e- 1	-0.0134	0.107	real	1.05	0.987	1.11

E.5.2 Shares from false news domains (main Allcott et al. 2019's classification)

Zero-inflated negative binomial regression (non-exponentiated and exponentiated 'e' coefficients)

A tibble: 21 × 11

	term	estimate	std.error	statistic	p.value	conf.low	conf.high	truth_subset	estimate_e	conf.low_e	conf.high_e
	<chr>	<dbl>	<dbl>	<dbl>	<dbl>	<dbl>	<dbl>	<chr>	<dbl>	<dbl>	<dbl>
1	count_(Intercept)	-2.12	1.02	-2.07	0.0388	-4.12	-0.109	fake	1.20e- 1	1.62e- 2	8.96e- 1
2	count_source_netw_congen	1.14	0.221	5.16	0.000000270	0.708	1.58	fake	3.13e+ 0	2.03e+ 0	4.83e+ 0
3	count_female1	-0.117	0.465	-0.251	0.802	-1.03	0.794	fake	8.90e- 1	3.58e- 1	2.21e+ 0
4	count_white1	-0.0139	0.449	-0.0309	0.975	-0.894	0.866	fake	9.86e- 1	4.09e- 1	2.38e+ 0
5	count_hh_income	-0.600	0.238	-2.52	0.0116	-1.07	-0.134	fake	5.49e- 1	3.44e- 1	8.75e- 1
6	count_highered1	-0.0330	0.467	-0.0706	0.944	-0.949	0.883	fake	9.68e- 1	3.87e- 1	2.42e+ 0
7	count_age2	-1.57	0.931	-1.69	0.0908	-3.40	0.250	fake	2.07e- 1	3.34e- 2	1.28e+ 0
8	count_age3	0.588	0.844	0.697	0.486	-1.07	2.24	fake	1.80e+ 0	3.44e- 1	9.43e+ 0
9	count_age4	2.59	1.16	2.24	0.0254	0.319	4.86	fake	1.33e+ 1	1.38e+ 0	1.29e+ 2
10	count_followers	-0.0951	0.0559	-1.70	0.0890	-0.205	0.0145	fake	9.09e- 1	8.15e- 1	1.01e+ 0
11	count_partyid	0.343	0.102	3.37	0.000753	0.144	0.543	fake	1.41e+ 0	1.15e+ 0	1.72e+ 0
12	zero_(Intercept)	-13.2	6.95	-1.90	0.0576	-26.8	0.428	fake	1.84e- 6	2.21e- 12	1.53e+ 0
13	zero_followers_likemindedness	-0.103	0.206	-0.501	0.616	-0.508	0.301	fake	9.02e- 1	6.02e- 1	1.35e+ 0
14	zero_female1	0.00931	0.593	0.0157	0.987	-1.15	1.17	fake	1.01e+ 0	3.16e- 1	3.23e+ 0
15	zero_hh_income	-0.322	0.316	-1.02	0.308	-0.941	0.297	fake	7.25e- 1	3.90e- 1	1.35e+ 0
16	zero_highered1	0.253	0.586	0.431	0.666	-0.897	1.40	fake	1.29e+ 0	4.08e- 1	4.06e+ 0
17	zero_age2	-1.99	1.10	-1.82	0.0691	-4.14	0.156	fake	1.36e- 1	1.59e- 2	1.17e+ 0
18	zero_age3	-1.93	0.822	-2.34	0.0192	-3.54	-0.314	fake	1.46e- 1	2.90e- 2	7.30e- 1
19	zero_age4	-0.153	1.19	-0.128	0.898	-2.49	2.19	fake	8.58e- 1	8.28e- 2	8.90e+ 0
20	zero_followers	-145.	59.7	-2.43	0.0152	-262.	-28.0	fake	1.08e-63	1.64e-114	7.09e-13
21	zero_partyid	-0.196	0.118	-1.67	0.0957	-0.428	0.0346	fake	8.22e- 1	6.52e- 1	1.04e+ 0

Zero-inflated negative binomial regression, without 1% greatest sharers (non-exponentiated and exponentiated 'e' coefficients)

```
# A tibble: 21 × 11
   term                         estimate std.error statistic  p.value  conf.low conf.high truth_subset estimate_e conf.low_e conf.high_e
   <chr>                           <dbl>     <dbl>     <dbl>    <dbl>     <dbl>     <dbl> <chr>             <dbl>      <dbl>       <dbl>
 1 count_(Intercept)             -1.08      0.923     -1.17   0.243    -2.89       0.732  fake              0.340    0.0556          2.08
 2 count_source_netw_congen       0.356     0.165      2.16   0.0306    0.0333     0.679  fake              1.43     1.03            1.97
 3 count_female1                  0.0827    0.291      0.284  0.776    -0.488      0.653  fake              1.09     0.614           1.92
 4 count_white1                   0.200     0.307      0.653  0.514    -0.401      0.802  fake              1.22     0.669           2.23
 5 count_hh_income               -0.130     0.160     -0.812  0.417    -0.445      0.184  fake              0.878    0.641           1.20
 6 count_highered1               -0.288     0.300     -0.960  0.337    -0.878      0.301  fake              0.749    0.416           1.35
 7 count_age2                    -0.131     0.753     -0.174  0.862    -1.61       1.35   fake              0.877    0.200           3.84
 8 count_age3                     0.322     0.677      0.475  0.635    -1.01       1.65   fake              1.38     0.366           5.20
 9 count_age4                     0.230     0.721      0.319  0.750    -1.18       1.64   fake              1.26     0.306           5.17
10 count_partyid                  0.175     0.0869     2.01   0.0445    0.00432    0.345  fake              1.19     1.00            1.41
11 count_followers               -0.285     0.400     -0.713  0.476    -1.07       0.499  fake              0.752    0.343           1.65
12 zero_(Intercept)               3.56      1.01       3.52   0.000446  1.58       5.55   fake             35.3      4.84          257.
13 zero_followers_likemindedness -0.167     0.121     -1.38   0.167    -0.405      0.0698 fake              0.846    0.667           1.07
14 zero_female1                   0.0675    0.318      0.212  0.832    -0.556      0.691  fake              1.07     0.573           2.00
15 zero_hh_income                 0.0539    0.177      0.304  0.761    -0.293      0.401  fake              1.06     0.746           1.49
16 zero_highered1                -0.409     0.324     -1.26   0.207    -1.04       0.226  fake              0.664    0.352           1.25
17 zero_age2                     -0.464     0.758     -0.612  0.540    -1.95       1.02   fake              0.629    0.142           2.78
18 zero_age3                     -1.20      0.697     -1.72   0.0861   -2.56       0.170  fake              0.302    0.0771          1.19
19 zero_age4                     -0.924     0.836     -1.10   0.269    -2.56       0.716  fake              0.397    0.0770          2.05
20 zero_partyid                  -0.0179    0.0917    -0.195  0.845    -0.198      0.162  fake              0.982    0.821           1.18
21 zero_followers                -3.68      3.28      -1.12   0.261   -10.1        2.74   fake              0.0252   0.0000409      15.5
```

Poisson regression (non-exponentiated and exponentiated 'e' coefficients)

```
# A tibble: 11 × 11
   term               estimate std.error statistic  p.value conf.low conf.high truth_subset estimate_e conf.low_e conf.high_e
   <chr>                 <dbl>     <dbl>     <dbl>    <dbl>    <dbl>     <dbl> <chr>             <dbl>      <dbl>       <dbl>
 1 (Intercept)         -5.23      1.60      -3.28  1.05e- 3  -8.36      -2.10  fake            0.00535    0.000234        0.122
 2 source_netw_congen   0.975     0.131      7.43  1.09e-13   0.717      1.23  fake            2.65       2.05            3.43
 3 female1              0.483     0.799      0.605 5.45e- 1  -1.08       2.05  fake            1.62       0.339           7.75
 4 white1              -0.213     0.860     -0.248 8.04e- 1  -1.90       1.47  fake            0.808      0.150           4.36
 5 hh_income           -0.138     0.197     -0.703 4.82e- 1  -0.524      0.247 fake            0.871      0.592           1.28
 6 highered1            1.47      0.548      2.68  7.37e- 3   0.394      2.54  fake            4.34       1.48           12.7
 7 age2                -1.02      0.994     -1.03  3.04e- 1  -2.97       0.927 fake            0.360      0.0512          2.53
 8 age3                 1.49      1.05       1.42  1.55e- 1  -0.564      3.55  fake            4.44       0.569          34.7
 9 age4                 3.00      1.09       2.75  5.99e- 3   0.859      5.13  fake           20.0        2.36          169.
10 followers            0.0968    0.0320     3.02  2.50e- 3   0.0341     0.160 fake            1.10       1.03            1.17
11 partyid              0.447     0.0890     5.02  5.08e- 7   0.273      0.622 fake            1.56       1.31            1.86
```

F. Analyses of Study 2 (intentions to share)

F.1 Proportion of Democrats and Republicans as a function of exclusion criteria

Study 2	Total N	number Democrats	prop Democrats	number Republicans	prop Republicans	number independents	prop independents
Before any exclusion	1829	901	0.492618917	869	0.475123018	28	0.015308912
After removing non-partisans	1770	901	0.509039548	869	0.490960452	0	0
After removing inattentive respondents	1735	877	0.505475504	858	0.494524496	0	0

F.2 Plot: Sharing supplemented with density plots for raw data

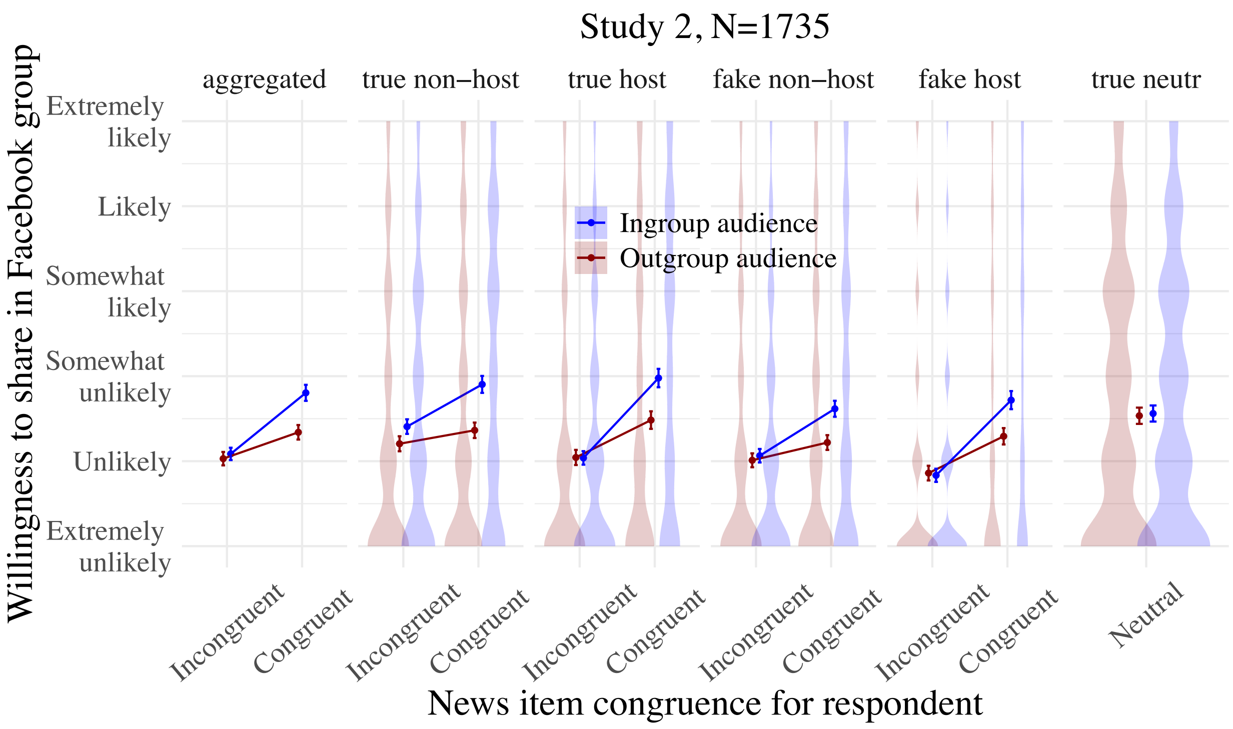

Supplementary figure 10.

F.3 Mean values of intentions to share

Sharing by Audience Type and News Congruence - aggregated

News Congruence	Audience Type	Mean Value
Incongruent	Outgroup audience	1.029671
Incongruent	Ingroup audience	1.085678
Congruent	Outgroup audience	1.339749
Congruent	Ingroup audience	1.803993
Neutral	Outgroup audience	1.535457
Neutral	Ingroup audience	1.560803

Sharing by Audience Type and News Congruence - true non-host

News Congruence	Audience Type	Mean value
Incongruent	Outgroup audience	1.204892
Incongruent	Ingroup audience	1.406766
Congruent	Outgroup audience	1.363381
Congruent	Ingroup audience	1.903226

Sharing by Audience Type and News Congruence - true host

News Congruence	Audience Type	Mean Value
Incongruent	Outgroup audience	1.043722
Incongruent	Ingroup audience	1.038174
Congruent	Outgroup audience	1.483353
Congruent	Ingroup audience	1.977979

Sharing by Audience Type and News Congruence - fake non-host

News Congruence	Audience Type	Mean Value
Incongruent	Outgroup audience	1.010425

News Congruence	Audience Type	Mean Value
Incongruent	Ingroup audience	1.064148
Congruent	Outgroup audience	1.219326
Congruent	Ingroup audience	1.616444

Sharing by Audience Type and News Congruence - fake host

News Congruence	Audience Type	Mean Value
Incongruent	Outgroup audience	0.8597194
Incongruent	Ingroup audience	0.8335956
Congruent	Outgroup audience	1.2929374
Congruent	Ingroup audience	1.7182212

F.4 Regressions: sharing averaging across all partisan news (baseline: Outgroup audience)

NB: those tables were printed with the tab_model() function in R, which automatically outputs standardized coefficients by default.

Congruence & Congruence x audience

Stu 2 - averaging across all partisan news

	w2s			w2s		
Predictors	*Estimates*	*CI*	*p*	*Estimates*	*CI*	*p*
(Intercept)	-0.17	-0.21,-0.14	**<0.001**	1.03	0.95,1.11	**<0.001**
news congruence [Congruent]	0.32	0.29,0.35	**<0.001**	0.31	0.24,0.38	**<0.001**
Audience InOut [Ingroup audience]				0.06	-0.05,0.16	0.306
news congruence [Congruent] × Audience InOut [Ingroup audience]				0.41	0.31,0.51	**<0.001**
Observations	40285			40285		

R^2 / R^2 adjusted	0.026 / 0.026	0.037 / 0.037

Audience on congruent news only

Stu 2 - congruent news only - averaging across all partisan news

	w2s		
Predictors	*Estimates*	*CI*	*p*
(Intercept)	0.00	-0.05,0.06	0.893
Audience InOut [Ingroup audience]	0.29	0.21,0.37	**<0.001**
Observations	20143		
R^2 / R^2 adjusted	0.018 / 0.018		

Audience on incongruent news only

Stu 2 - incongruent news only - averaging across all partisan news

	w2s		
Predictors	*Estimates*	*CI*	*p*
(Intercept)	-0.19	-0.24,-0.14	**<0.001**
Audience InOut [Ingroup audience]	0.03	-0.03,0.10	0.306
Observations	20142		
R^2 / R^2 adjusted	0.000 / 0.000		

F.5 Regressions: sharing true neutral news (baseline: Outgroup audience)

NB: those tables were printed with the tab_model() function in R, which automatically outputs standardized coefficients by default.

Audience on true neutral news

Stu 2 - true neutral news only

	w2s		
Predictors	*Estimates*	*CI*	*p*

(Intercept)	1.53	1.44,1.63	**<0.001**
Audience InOut [Ingroup audience]	0.03	-0.11,0.16	0.700
**Random Effects**			
σ^2	0.95		
$\tau_{00\ id}$	1.51		
ICC	0.61		
N_{id}	1680		
Observations	3358		
Marginal R^2 / Conditional R^2	0.000 / 0.613		

F.6 Regressions: sharing breaking down by partisan news types (baseline: Outgroup audience)

NB: those tables were printed with the tab_model() function in R, which automatically outputs standardized coefficients by default.

Audience on congruent news only

Stu 2 - congruent true non-host partisan news

	w2s		
Predictors	*Estimates*	*CI*	*p*
(Intercept)	0.02	-0.04,0.08	0.525
Audience InOut [Ingroup audience]	0.34	0.25,0.42	**<0.001**
Observations	5038		
R^2 / R^2 adjusted	0.026 / 0.025		

Stu 2 - congruent true host partisan news

	w2s		
Predictors	*Estimates*	*CI*	*p*
(Intercept)	0.09	0.03,0.16	**0.005**
Audience InOut [Ingroup audience]	0.31	0.22,0.40	**<0.001**

Observations	5036
R^2 / R^2 adjusted	0.020 / 0.020

Stu 2 - congruent false non-host partisan news

	w2s		
Predictors	*Estimates*	*CI*	*p*
(Intercept)	-0.07	-0.13,-0.02	**0.010**
Audience InOut [Ingroup audience]	0.25	0.17,0.33	**<0.001**
Observations	5036		
R^2 / R^2 adjusted	0.015 / 0.015		

Stu 2 - congruent false host partisan news

	w2s		
Predictors	*Estimates*	*CI*	*p*
(Intercept)	-0.03	-0.09,0.03	0.405
Audience InOut [Ingroup audience]	0.27	0.18,0.36	**<0.001**
Observations	5033		
R^2 / R^2 adjusted	0.015 / 0.015		

Audience on incongruent news only

Stu 2 - incongruent true non-host partisan news

	w2s		
Predictors	*Estimates*	*CI*	*p*
(Intercept)	-0.08	-0.14,-0.03	**0.004**
Audience InOut [Ingroup audience]	0.13	0.05,0.20	**0.001**
Observations	5036		
R^2 / R^2 adjusted	0.004 / 0.004		

Stu 2 - incongruent true host partisan news

	w2s		
Predictors	*Estimates*	*CI*	*p*
(Intercept)	-0.18	-0.24,-0.13	**<0.001**
Audience InOut [Ingroup audience]	-0.00	-0.08,0.07	0.927
Observations	5034		
R^2 / R^2 adjusted	0.000 / -0.000		

Stu 2 - incongruent false non-host partisan news

	w2s		
Predictors	*Estimates*	*CI*	*p*
(Intercept)	-0.20	-0.25,-0.15	**<0.001**
Audience InOut [Ingroup audience]	0.03	-0.04,0.10	0.356
Observations	5035		
R^2 / R^2 adjusted	0.000 / 0.000		

Stu 2 - incongruent false host partisan news

	w2s		
Predictors	*Estimates*	*CI*	*p*
(Intercept)	-0.30	-0.35,-0.24	**<0.001**
Audience InOut [Ingroup audience]	-0.02	-0.09,0.06	0.659
Observations	5037		
R^2 / R^2 adjusted	0.0 -0.000		

G. Analyses of Study 3 (intentions to share)

G.1 Proportion of Democrats and Republicans as a function of exclusion criteria

Study 3	Total N	number Democrats	prop Democrats	number Republicans	prop Repub licans	number independents	prop inde pendents
Before any exclusion	1743	814	0.467010901	823	0.472174412	17	0.009753299
After removing non-partisans	1637	814	0.497251069	823	0.502748931	0	0
After removing inattentive respondents	1637	814	0.497251069	823	0.502748931	0	0

G.2 Plot: Sharing supplemented with density plots for raw data

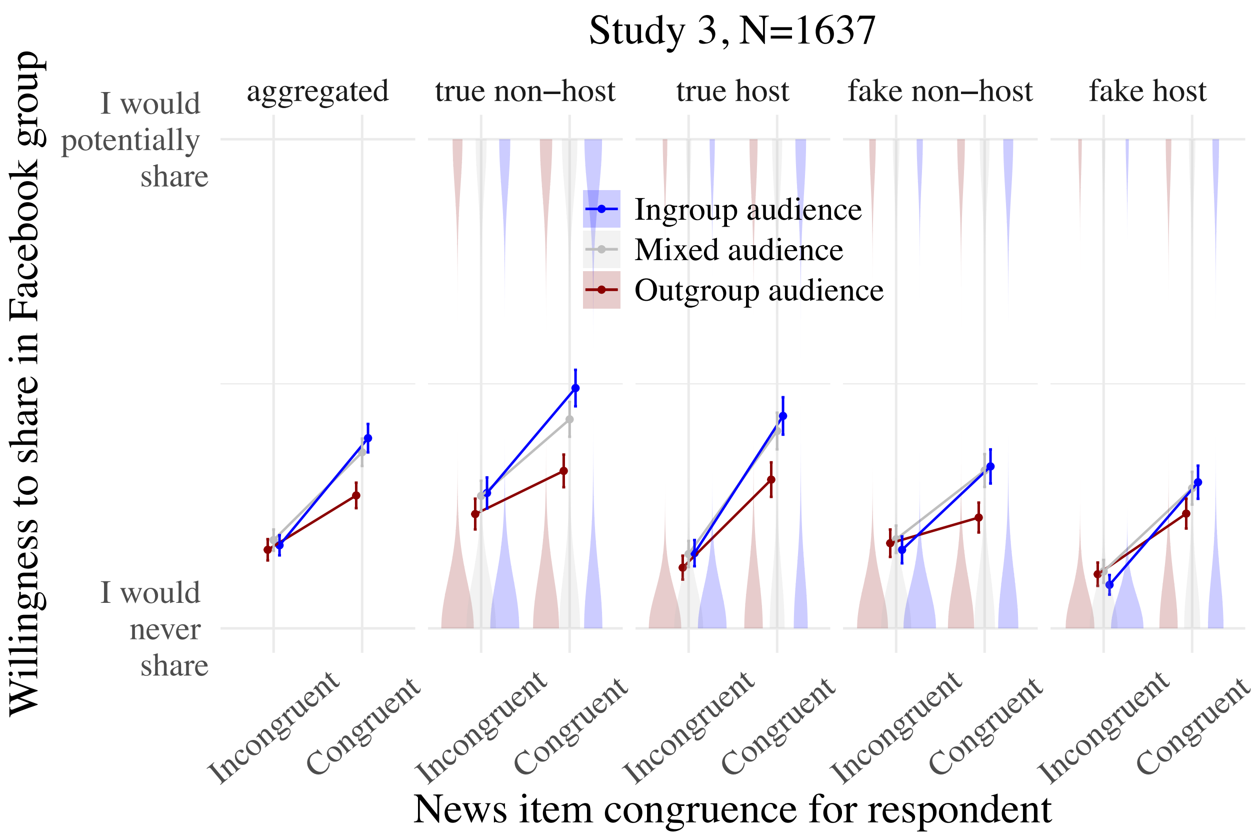

Supplementary figure 11.

G.3 Mean values of intentions to share

Sharing by Audience Type and News Congruence - aggregated

News Congruence	Audience Type	Mean Value
Incongruent	Outgroup audience	0.1606621
Incongruent	Mixed audience	0.1804311
Incongruent	Ingroup audience	0.1698663
Congruent	Outgroup audience	0.2717736
Congruent	Mixed audience	0.3599806
Congruent	Ingroup audience	0.3888619

Sharing by Audience Type and News Congruence - true non-host

News Congruence	Audience Type	Mean Value
Incongruent	Outgroup audience	0.2336904
Incongruent	Mixed audience	0.2707930
Incongruent	Ingroup audience	0.2768031
Congruent	Outgroup audience	0.3219178
Congruent	Mixed audience	0.4273256
Congruent	Ingroup audience	0.4912621

Sharing by Audience Type and News Congruence - true host

News Congruence	Audience Type	Mean Value
Incongruent	Outgroup audience	0.1241447
Incongruent	Mixed audience	0.1519845
Incongruent	Ingroup audience	0.1538462
Congruent	Outgroup audience	0.3040936
Congruent	Mixed audience	0.4034918
Congruent	Ingroup audience	0.4342746

Sharing by Audience Type and News Congruence - fake non-host

News Congruence	Audience Type	Mean Value
Incongruent	Outgroup audience	0.1741245
Incongruent	Mixed audience	0.1821705
Incongruent	Ingroup audience	0.1605058
Congruent	Outgroup audience	0.2265625
Congruent	Mixed audience	0.3226744
Congruent	Ingroup audience	0.3310547

Sharing by Audience Type and News Congruence - fake host

News Congruence	Audience Type	Mean Value
Incongruent	Outgroup audience	0.11067961
Incongruent	Mixed audience	0.11650485
Incongruent	Ingroup audience	0.08897485
Congruent	Outgroup audience	0.23466407
Congruent	Mixed audience	0.28654405
Congruent	Ingroup audience	0.29873909

G.4 Regressions: sharing averaging across all partisan news types (baseline: Outgroup audience)

Reported in main text.

NB: those tables were printed with the tab_model() function in R, which automatically outputs standardized coefficients by default.

Congruence & Congruence x audience

Stu 3 - sharing - averaging across all partisan news

		value			value	
Predictors	*Estimates*	*CI*	*p*	*Estimates*	*CI*	*p*

(Intercept)	0.17	0.16,0.18	**<0.001**	0.16	0.14,0.18	**<0.001**
news congruence [Congruent]	0.17	0.16,0.18	**<0.001**	0.11	0.09,0.14	**<0.001**
Audience InMixOut [Mixed audience]				0.02	-0.01,0.05	0.208
Audience InMixOut [Ingroup audience]				0.01	-0.02,0.04	0.546
news congruence [Congruent] × Audience InMixOut [Mixed audience]				0.07	0.03,0.10	**<0.001**
news congruence [Congruent] × Audience InMixOut [Ingroup audience]				0.11	0.07,0.14	**<0.001**
Observations	24691			24691		
R^2 / R^2 adjusted	0.038 / 0.038			0.045 / 0.044		

Audience on congruent news only

Stu 3 - sharing - congruent news only - averaging across all partisan news

		value	
Predictors	*Estimates*	*CI*	*p*
(Intercept)	0.27	0.25,0.30	**<0.001**
Audience InMixOut [Mixed audience]	0.09	0.05,0.13	**<0.001**
Audience InMixOut [Ingroup audience]	0.12	0.08,0.16	**<0.001**
Observations	12339		
R^2 / R^2 adjusted	0.011 / 0.011		

Audience on incongruent news only

Stu 3 - sharing - incongruent news only - averaging across all partisan news

		value	
Predictors	*Estimates*	*CI*	*p*

(Intercept)	0.16	0.14,0.18	**<0.001**
Audience InMixOut [Mixed audience]	0.02	-0.01,0.05	0.208
Audience InMixOut [Ingroup audience]	0.01	-0.02,0.04	0.546
Observations	12352		
R^2 / R^2 adjusted	0.000 / 0.000		

G.5 Regressions: sharing breaking down by partisan news types (baseline: Outgroup audience)

Reported in main text.

NB: those tables were printed with the tab_model() function in R, which automatically outputs standardized coefficients by default.

Audience on congruent news only

**Stu 3 - sharing - congruent true non-host partisan news**			
		**value**	
Predictors	*Estimates*	*CI*	*p*
(Intercept)	0.32	0.29,0.36	**<0.001**
Audience InMixOut [Mixed audience]	0.11	0.06,0.15	**<0.001**
Audience InMixOut [Ingroup audience]	0.17	0.12,0.22	**<0.001**
Observations	3084		
R^2 / R^2 adjusted	0.020 / 0.019		

**Stu 3 - sharing - congruent true host partisan news**			
		**value**	
Predictors	*Estimates*	*CI*	*p*
(Intercept)	0.30	0.27,0.34	**<0.001**

Audience InMixOut [Mixed audience]	0.10	0.05,0.15	**<0.001**
Audience InMixOut [Ingroup audience]	0.13	0.08,0.18	**<0.001**
Observations	3084		
R^2 / R^2 adjusted	0.013 / 0.012		

Stu 3 - sharing - congruent false non-host partisan news

		**value**	
Predictors	*Estimates*	*CI*	*p*
(Intercept)	0.23	0.20,0.26	**<0.001**
Audience InMixOut [Mixed audience]	0.10	0.05,0.14	**<0.001**
Audience InMixOut [Ingroup audience]	0.10	0.06,0.15	**<0.001**
Observations	3080		
R^2 / R^2 adjusted	0.011 / 0.010		

Stu 3 - sharing - congruent false host partisan news

		**value**	
Predictors	*Estimates*	*CI*	*p*
(Intercept)	0.23	0.20,0.27	**<0.001**
Audience InMixOut [Mixed audience]	0.05	0.01,0.10	**0.025**
Audience InMixOut [Ingroup audience]	0.06	0.02,0.11	**0.006**
Observations	3091		
R^2 / R^2 adjusted	0.004 / 0.003		

Audience on incongruent news only

Stu 3 - sharing - incongruent true non-host partisan news

	value		
Predictors	*Estimates*	*CI*	*p*
(Intercept)	0.23	0.20,0.27	**<0.001**
Audience InMixOut [Mixed audience]	0.04	-0.01,0.08	0.099
Audience InMixOut [Ingroup audience]	0.04	-0.00,0.09	0.058
Observations	3087		
R^2 / R^2 adjusted	0.002 / 0.001		

Stu 3 - sharing - incongruent true host partisan news

	value		
Predictors	*Estimates*	*CI*	*p*
(Intercept)	0.12	0.10,0.15	**<0.001**
Audience InMixOut [Mixed audience]	0.03	-0.01,0.06	0.131
Audience InMixOut [Ingroup audience]	0.03	-0.01,0.07	0.108
Observations	3083		
R^2 / R^2 adjusted	0.001 / 0.001		

Stu 3 - sharing - incongruent false non-host partisan news

	value		
Predictors	*Estimates*	*CI*	*p*
(Intercept)	0.17	0.15,0.20	**<0.001**
Audience InMixOut [Mixed audience]	0.01	-0.03,0.05	0.690
Audience InMixOut [Ingroup audience]	-0.01	-0.05,0.03	0.496
Observations	3088		
R^2 / R^2 adjusted	0.001 / -0.000		

Stu 3 - sharing - incongruent false host partisan news

	value		
Predictors	*Estimates*	*CI*	*p*
(Intercept)	0.11	0.09,0.13	**<0.001**
Audience InMixOut [Mixed audience]	0.01	-0.03,0.04	0.732
Audience InMixOut [Ingroup audience]	-0.02	-0.05,0.01	0.172
Observations	3094		
R^2 / R^2 adjusted	0.001 / 0.001		

H. Analyses of Study 3 (motivations for sharing and not sharing)

H.1 Mean values of motivations to share

Motivations to Share by Audience Type and News Congruence

**Motivation**	**News Congruence**	**Audience Type**	**Mean Value**
affirm moral conviction	Congruent	Outgroup audience	3.692998
affirm moral conviction	Congruent	Mixed audience	3.666891
affirm moral conviction	Congruent	Ingroup audience	3.617886
affirm moral conviction	Incongruent	Outgroup audience	3.374242
affirm moral conviction	Incongruent	Mixed audience	3.395973
affirm moral conviction	Incongruent	Ingroup audience	3.449213
audience could help fact-check	Congruent	Outgroup audience	3.027828
audience could help fact-check	Congruent	Mixed audience	3.526918
audience could help fact-check	Congruent	Ingroup audience	3.540338
audience could help fact-check	Incongruent	Outgroup audience	3.431818
audience could help fact-check	Incongruent	Mixed audience	3.782550
audience could help fact-check	Incongruent	Ingroup audience	3.781116
audience would like me more (index)	Congruent	Outgroup audience	1.972771
audience would like me more (index)	Congruent	Mixed audience	2.261328
audience would like me more (index)	Congruent	Ingroup audience	2.414009
audience would like me more (index)	Incongruent	Outgroup audience	2.512626
audience would like me more (index)	Incongruent	Mixed audience	2.277405
audience would like me more (index)	Incongruent	Ingroup audience	2.331426
story is true	Congruent	Outgroup audience	3.933573
story is true	Congruent	Mixed audience	3.965680
story is true	Congruent	Ingroup audience	3.938712

Motivation	News Congruence	Audience Type	Mean Value
story is true	Incongruent	Outgroup audience	3.559091
story is true	Incongruent	Mixed audience	3.542282
story is true	Incongruent	Ingroup audience	3.597997
useful to mobilize audience (index)	Congruent	Outgroup audience	2.503291
useful to mobilize audience (index)	Congruent	Mixed audience	2.687528
useful to mobilize audience (index)	Congruent	Ingroup audience	2.640609
useful to mobilize audience (index)	Incongruent	Outgroup audience	2.462626
useful to mobilize audience (index)	Incongruent	Mixed audience	2.435794
useful to mobilize audience (index)	Incongruent	Ingroup audience	2.509299

H.2 Mean values of motivations not to share

Motivations to Not Share by Audience Type and News Congruence

Motivation	News Congruence	Audience Type	Mean Value
audience cannot help fact-check	Congruent	Outgroup audience	3.077722
audience cannot help fact-check	Congruent	Mixed audience	2.698713
audience cannot help fact-check	Congruent	Ingroup audience	2.621170
audience cannot help fact-check	Incongruent	Outgroup audience	3.139501
audience cannot help fact-check	Incongruent	Mixed audience	2.767435
audience cannot help fact-check	Incongruent	Ingroup audience	2.574063
audience would like me less (index)	Congruent	Outgroup audience	2.327080
audience would like me less (index)	Congruent	Mixed audience	2.143326
audience would like me less (index)	Congruent	Ingroup audience	1.881019
audience would like me less (index)	Incongruent	Outgroup audience	2.050947
audience would like me less (index)	Incongruent	Mixed audience	2.162924
audience would like me less (index)	Incongruent	Ingroup audience	2.113973

Motivation	News Congruence	Audience Type	Mean Value
go against moral conviction	Congruent	Outgroup audience	2.802345
go against moral conviction	Congruent	Mixed audience	2.728615
go against moral conviction	Congruent	Ingroup audience	2.746120
go against moral conviction	Incongruent	Outgroup audience	3.138631
go against moral conviction	Incongruent	Mixed audience	2.971927
go against moral conviction	Incongruent	Ingroup audience	2.980386
not useful to mobilize audience (index)	Congruent	Outgroup audience	2.432831
not useful to mobilize audience (index)	Congruent	Mixed audience	2.187484
not useful to mobilize audience (index)	Congruent	Ingroup audience	1.997347
not useful to mobilize audience (index)	Incongruent	Outgroup audience	2.434938
not useful to mobilize audience (index)	Incongruent	Mixed audience	2.288613
not useful to mobilize audience (index)	Incongruent	Ingroup audience	2.150468
story is false	Congruent	Outgroup audience	2.905528
story is false	Congruent	Mixed audience	3.046556
story is false	Congruent	Ingroup audience	3.065659
story is false	Incongruent	Outgroup audience	3.488399
story is false	Incongruent	Mixed audience	3.565898
story is false	Incongruent	Ingroup audience	3.526347

H.3 Tests comparing motivations for sharing

Those comparisons were made on the mean scores of each motivation for sharing (averaging across congruent and incongruent news and audience)
Baseline: 'Audience would like me more

```
# A tibble: 5 × 7
  term                                         estimate std.error statistic   p.value conf.low conf.high
  <chr>                                           <dbl>     <dbl>     <dbl>     <dbl>    <dbl>     <dbl>
1 (Intercept)                                     2.29     0.0230      99.4 0            2.24      2.33
2 motivationuseful to mobilize audience (index)   0.285    0.0167      17.1 5.80e- 58    0.252     0.318
3 motivationaudience could help fact-check        1.21     0.0303      39.8 1.31e-213    1.15      1.27
4 motivationstory is true                         1.53     0.0301      50.9 2.45e-289    1.48      1.59
5 motivationaffirm moral conviction               1.29     0.0265      48.5 2.63e-273    1.24      1.34
```

H.4 Principal component analysis of the main motivations for sharing

NB: This analysis focuses on the 5 main motivations for sharing we decided to plot and report in the paper (Study 3). We only provide PCA outputs for the motivations to share the news (motivations for not sharing were not analyzed), breaking down by news congruence and truth vs. falsity, and exclude data from the Mixed audience condition, in order to focus on the Ingroup and Outgroup audiences.

Ingroup audience, congruent true news

```
Principal Components Analysis
Call: principal(r = mot_to_share_wide_Inaud_t_cong, nfactors = 5, rotate
= "varimax")
Standardized loadings (pattern matrix) based upon correlation matrix
                                     RC1  RC3  RC2  RC4  RC5 h2       u2
com
affirm moral conviction             0.24 0.17 0.22 0.89 0.26  1 -6.7e-16
1.6
audience could help fact-check      0.18 0.96 0.04 0.15 0.16  1  1.0e-15
1.2
audience would like me more (index) 0.91 0.20 0.05 0.23 0.28  1  1.1e-15
1.5
story is true                       0.04 0.04 0.98 0.16 0.06  1  8.9e-16
1.1
useful to mobilize audience (index) 0.41 0.25 0.09 0.35 0.80  1  4.4e-16
2.2

                       RC1  RC3  RC2  RC4  RC5
SS loadings           1.09 1.05 1.03 1.02 0.82
Proportion Var        0.22 0.21 0.21 0.20 0.16
Cumulative Var        0.22 0.43 0.63 0.84 1.00
Proportion Explained  0.22 0.21 0.21 0.20 0.16
Cumulative Proportion 0.22 0.43 0.63 0.84 1.00

Mean item complexity =  1.5
Test of the hypothesis that 5 components are sufficient.

The root mean square of the residuals (RMSR) is  0
 with the empirical chi square  0  with prob <  NA

Fit based upon off diagonal values = 1
```

Ingroup audience, incongruent true news

```
Principal Components Analysis
Call: principal(r = mot_to_share_wide_Inaud_t_incong, nfactors = 5,
    rotate = "varimax")
Standardized loadings (pattern matrix) based upon correlation matrix
                                      RC1  RC4  RC2  RC3  RC5 h2       u2
com
affirm moral conviction              0.23 0.91 0.18 0.12 0.26  1 -4.4e-16
1.4
audience could help fact-check       0.14 0.10 0.03 0.98 0.11  1  1.2e-15
1.1
audience would like me more (index) 0.91 0.23 0.11 0.17 0.29  1  1.1e-15
1.4
story is true                        0.10 0.15 0.98 0.03 0.12  1 -2.2e-16
1.1
useful to mobilize audience (index) 0.39 0.35 0.17 0.17 0.81  1  7.8e-16
2.1

                       RC1  RC4  RC2  RC3  RC5
SS loadings           1.06 1.04 1.03 1.03 0.84
Proportion Var        0.21 0.21 0.21 0.21 0.17
Cumulative Var        0.21 0.42 0.63 0.83 1.00
Proportion Explained  0.21 0.21 0.21 0.21 0.17
Cumulative Proportion 0.21 0.42 0.63 0.83 1.00

Mean item complexity =  1.4
Test of the hypothesis that 5 components are sufficient.

The root mean square of the residuals (RMSR) is  0
 with the empirical chi square  0  with prob <  NA

Fit based upon off diagonal values = 1
```

Ingroup audience, congruent false news

```
Principal Components Analysis
Call: principal(r = mot_to_share_wide_Inaud_f_cong, nfactors = 5, rotate
= "varimax")
Standardized loadings (pattern matrix) based upon correlation matrix
                                      RC1  RC2  RC3  RC4  RC5 h2       u2
com
affirm moral conviction              0.17 0.23 0.10 0.92 0.23  1 -4.4e-16
1.4
audience could help fact-check       0.12 0.04 0.98 0.09 0.11  1 -1.3e-15
1.1
audience would like me more (index) 0.93 0.08 0.14 0.17 0.30  1 -6.7e-16
1.3
story is true                        0.07 0.97 0.04 0.20 0.11  1  0.0e+00
1.1
useful to mobilize audience (index) 0.34 0.13 0.13 0.25 0.89  1 -2.2e-16
1.6

                       RC1  RC2  RC3  RC4  RC5
SS loadings           1.02 1.02 1.01 1.00 0.95
Proportion Var        0.20 0.20 0.20 0.20 0.19
Cumulative Var        0.20 0.41 0.61 0.81 1.00
Proportion Explained  0.20 0.20 0.20 0.20 0.19
Cumulative Proportion 0.20 0.41 0.61 0.81 1.00

Mean item complexity =  1.3
Test of the hypothesis that 5 components are sufficient.

The root mean square of the residuals (RMSR) is  0
 with the empirical chi square  0  with prob <  NA

Fit based upon off diagonal values = 1
```

Ingroup audience, incongruent false news

```
Principal Components Analysis
Call: principal(r = mot_to_share_wide_Inaud_f_incong, nfactors = 5,
    rotate = "varimax")
Standardized loadings (pattern matrix) based upon correlation matrix
                                    RC5  RC3  RC2  RC4  RC1 h2       u2
com
affirm moral conviction             0.21 0.24 0.08 0.92 0.21  1 -6.7e-16
1.4
audience could help fact-check      0.11 0.05 0.99 0.07 0.10  1  3.3e-16
1.1
audience would like me more (index) 0.90 0.12 0.13 0.21 0.33  1  1.2e-15
1.5
story is true                       0.11 0.96 0.05 0.21 0.14  1  7.8e-16
1.2
useful to mobilize audience (index) 0.41 0.20 0.14 0.26 0.84  1  6.7e-16
1.9

                       RC5  RC3  RC2  RC4  RC1
SS loadings           1.05 1.03 1.02 1.01 0.89
Proportion Var        0.21 0.21 0.20 0.20 0.18
Cumulative Var        0.21 0.42 0.62 0.82 1.00
Proportion Explained  0.21 0.21 0.20 0.20 0.18
Cumulative Proportion 0.21 0.42 0.62 0.82 1.00

Mean item complexity =  1.4
Test of the hypothesis that 5 components are sufficient.

The root mean square of the residuals (RMSR) is  0
 with the empirical chi square  0  with prob <  NA

Fit based upon off diagonal values = 1
```

Outgroup audience, congruent true news

```
Principal Components Analysis
Call: principal(r = mot_to_share_wide_Outaud_t_cong, nfactors = 5,
    rotate = "varimax")
Standardized loadings (pattern matrix) based upon correlation matrix
                                      RC5   RC3   RC2  RC4   RC1 h2
u2 com
affirm moral conviction             -0.07  0.22  0.96 0.17  0.00  1 -
1.1e-15 1.2
audience could help fact-check       0.94 -0.03 -0.07 0.17  0.29  1 -
2.2e-16 1.3
audience would like me more (index)  0.31 -0.13  0.00 0.23  0.91  1
3.3e-16 1.4
story is true                       -0.03  0.96  0.22 0.09 -0.11  1
4.4e-16 1.2
useful to mobilize audience (index)  0.18  0.10  0.18 0.94  0.21  1
5.6e-16 1.3

                       RC5  RC3  RC2  RC4  RC1
SS loadings           1.02 1.01 1.00 1.00 0.97
Proportion Var        0.20 0.20 0.20 0.20 0.19
Cumulative Var        0.20 0.40 0.61 0.81 1.00
Proportion Explained  0.20 0.20 0.20 0.20 0.19
Cumulative Proportion 0.20 0.40 0.61 0.81 1.00

Mean item complexity =  1.3
Test of the hypothesis that 5 components are sufficient.

The root mean square of the residuals (RMSR) is  0
 with the empirical chi square  0  with prob <  NA

Fit based upon off diagonal values = 1
```

Outgroup audience, incongruent true news

```
Principal Components Analysis
Call: principal(r = mot_to_share_wide_Outaud_t_incong, nfactors = 5,
    rotate = "varimax")
Standardized loadings (pattern matrix) based upon correlation matrix
                                     RC3   RC4   RC5   RC2  RC1 h2
u2 com
affirm moral conviction             0.06 -0.02  0.96  0.17 0.20  1 -4.4e-
16 1.2
audience could help fact-check      0.97  0.15  0.06  0.00 0.19  1  1.3e-
15 1.1
audience would like me more (index) 0.15  0.96 -0.02 -0.09 0.22  1 -6.7e-
16 1.2
story is true                       0.00 -0.08  0.16  0.98 0.04  1  6.7e-
16 1.1
useful to mobilize audience (index) 0.23  0.26  0.24  0.05 0.91  1  8.9e-
16 1.5

                       RC3  RC4  RC5  RC2  RC1
SS loadings           1.02 1.02 1.01 1.01 0.95
Proportion Var        0.20 0.20 0.20 0.20 0.19
Cumulative Var        0.20 0.41 0.61 0.81 1.00
Proportion Explained  0.20 0.20 0.20 0.20 0.19
Cumulative Proportion 0.20 0.41 0.61 0.81 1.00

Mean item complexity =  1.2
Test of the hypothesis that 5 components are sufficient.

The root mean square of the residuals (RMSR) is  0
 with the empirical chi square  0  with prob <  NA

Fit based upon off diagonal values = 1
```

Outgroup audience, congruent false news

```
Principal Components Analysis
Call: principal(r = mot_to_share_wide_Outaud_f_cong, nfactors = 5,
    rotate = "varimax")
Standardized loadings (pattern matrix) based upon correlation matrix
                                      RC2   RC3  RC4  RC5  RC1 h2
u2 com
affirm moral conviction              0.28  0.04 0.03 0.94 0.20  1 -8.9e-
16 1.3
audience could help fact-check      -0.06  0.96 0.22 0.04 0.14  1  1.7e-
15 1.2
audience would like me more (index)  0.00  0.23 0.95 0.03 0.22  1  5.6e-
16 1.2
story is true                        0.95 -0.06 0.00 0.27 0.13  1  6.7e-
16 1.2
useful to mobilize audience (index)  0.14  0.16 0.24 0.21 0.92  1  4.4e-
16 1.4

                       RC2  RC3  RC4 RC5  RC1
SS loadings           1.01 1.01 1.00 1.0 0.98
Proportion Var        0.20 0.20 0.20 0.2 0.20
Cumulative Var        0.20 0.40 0.61 0.8 1.00
Proportion Explained  0.20 0.20 0.20 0.2 0.20
Cumulative Proportion 0.20 0.40 0.61 0.8 1.00

Mean item complexity =  1.2
Test of the hypothesis that 5 components are sufficient.

The root mean square of the residuals (RMSR) is  0
 with the empirical chi square  0  with prob <  NA

Fit based upon off diagonal values = 1
```

Outgroup audience, incongruent false news

```
Principal Components Analysis
Call: principal(r = mot_to_share_wide_Outaud_f_incong, nfactors = 5,
    rotate = "varimax")
Standardized loadings (pattern matrix) based upon correlation matrix
                                      RC2   RC4  RC3  RC1  RC5 h2
u2 com
affirm moral conviction              0.07  0.23 0.17 0.91 0.29  1 -8.9e-
16 1.4
audience could help fact-check       0.14  0.14 0.95 0.16 0.16  1 -2.2e-
16 1.2
audience would like me more (index)  0.97 -0.04 0.13 0.06 0.17  1 -4.4e-
16 1.1
story is true                       -0.04  0.96 0.14 0.20 0.15  1  2.2e-
16 1.2
useful to mobilize audience (index)  0.23  0.18 0.19 0.31 0.88  1  1.1e-
16 1.6

                       RC2  RC4  RC3  RC1  RC5
SS loadings           1.02 1.02 1.01 1.00 0.94
Proportion Var        0.20 0.20 0.20 0.20 0.19
Cumulative Var        0.20 0.41 0.61 0.81 1.00
Proportion Explained  0.20 0.20 0.20 0.20 0.19
Cumulative Proportion 0.20 0.41 0.61 0.81 1.00

Mean item complexity =  1.3
Test of the hypothesis that 5 components are sufficient.

The root mean square of the residuals (RMSR) is  0
 with the empirical chi square  0  with prob <  NA

Fit based upon off diagonal values = 1
|
```

H.5 Regressions: motivations for sharing, averaging across all partisan news types (baseline: Outgroup audience)

Story is true

Stu 3 - mot to share: story is true - averaging across all congruent partisan news

		**value**	
Predictors	*Estimates*	*CI*	*p*
(Intercept)	3.93	3.85,4.02	**<0.001**
Audience InMixOut [Mixed audience]	0.03	-0.09,0.15	0.607
Audience InMixOut [Ingroup audience]	0.01	-0.10,0.11	0.924
Observations	4199		
R^2 / R^2 adjusted	0.000 / -0.000		

Stu 3 - mot to share: story is true - averaging across all incongruent partisan news

	value		
Predictors	*Estimates*	*CI*	*p*
(Intercept)	3.56	3.41,3.71	**<0.001**
Audience InMixOut [Mixed audience]	-0.02	-0.22,0.19	0.872
Audience InMixOut [Ingroup audience]	0.04	-0.15,0.23	0.687
Observations	2104		
R^2 / R^2 adjusted	0.000 / -0.001		

Affirm moral conviction

Stu 3 - mot to share: affirm moral conviction - averaging across all congruent partisan news

	value		
Predictors	*Estimates*	*CI*	*p*
(Intercept)	3.69	3.60,3.78	**<0.001**
Audience InMixOut [Mixed audience]	-0.03	-0.16,0.11	0.699
Audience InMixOut [Ingroup audience]	-0.08	-0.20,0.05	0.239
Observations	4199		
R^2 / R^2 adjusted	0.001 / 0.000		

Stu 3 - mot to share: affirm moral conviction - averaging across all incongruent partisan news

	value		
Predictors	*Estimates*	*CI*	*p*
(Intercept)	3.37	3.21,3.54	**<0.001**
Audience InMixOut [Mixed audience]	0.02	-0.19,0.24	0.844
Audience InMixOut [Ingroup audience]	0.07	-0.13,0.28	0.471

Observations	2104
R^2 / R^2 adjusted	0.001 / -0.000

Audience would like me more

Stu 3 - mot to share: audience would like me more (index) - averaging across all congruent partisan news

Predictors	Estimates	value<br>CI	p
(Intercept)	1.97	1.88,2.07	**<0.001**
Audience InMixOut [Mixed audience]	0.29	0.17,0.41	**<0.001**
Audience InMixOut [Ingroup audience]	0.44	0.32,0.56	**<0.001**
Observations	4199		
R^2 / R^2 adjusted	0.054 / 0.053		

Stu 3 - mot to share: audience would like me more (index) - averaging across all incongruent partisan news

Predictors	Estimates	value<br>CI	p
(Intercept)	2.51	2.41,2.61	**<0.001**
Audience InMixOut [Mixed audience]	-0.24	-0.38,-0.09	**0.001**
Audience InMixOut [Ingroup audience]	-0.18	-0.32,-0.04	**0.010**
Observations	2104		
R^2 / R^2 adjusted	0.019 / 0.018		

Useful to mobilize audiences

Stu 3 - mot to share: useful to mobilize audience (index) - averaging across all congruent partisan news

Predictors	Estimates	value<br>CI	p

(Intercept)	2.50	2.43,2.57	**<0.001**
Audience InMixOut [Mixed audience]	0.18	0.09,0.28	**<0.001**
Audience InMixOut [Ingroup audience]	0.14	0.04,0.23	**0.006**
Observations	4199		
R^2 / R^2 adjusted	0.012 / 0.011		

Stu 3 - mot to share: useful to mobilize audience (index) - averaging across all incongruent partisan news

		**value**	
Predictors	*Estimates*	*CI*	*p*
(Intercept)	2.46	2.36,2.57	**<0.001**
Audience InMixOut [Mixed audience]	-0.03	-0.17,0.11	0.707
Audience InMixOut [Ingroup audience]	0.05	-0.09,0.18	0.488
Observations	2104		
R^2 / R^2 adjusted	0.002 / 0.001		

Audience could help fact-check

Stu 3 - mot to share: audience could help fact-check - averaging across all congruent partisan news

		**value**	
Predictors	*Estimates*	*CI*	*p*
(Intercept)	3.03	2.89,3.16	**<0.001**
Audience InMixOut [Mixed audience]	0.50	0.32,0.67	**<0.001**
Audience InMixOut [Ingroup audience]	0.51	0.34,0.68	**<0.001**
Observations	4199		
R^2 / R^2 adjusted	0.035 / 0.034		

Stu 3 - mot to share: audience could help fact-check - averaging across all incongruent partisan news

		**value**	
Predictors	*Estimates*	*CI*	*p*
(Intercept)	3.43	3.28,3.58	**<0.001**
Audience InMixOut [Mixed audience]	0.35	0.15,0.55	**0.001**
Audience InMixOut [Ingroup audience]	0.35	0.16,0.54	**<0.001**
Observations	2104		
R^2 / R^2 adjusted	0.021 / 0.020		

H.6 Plot: motivations for sharing supplemented with density plots for raw data

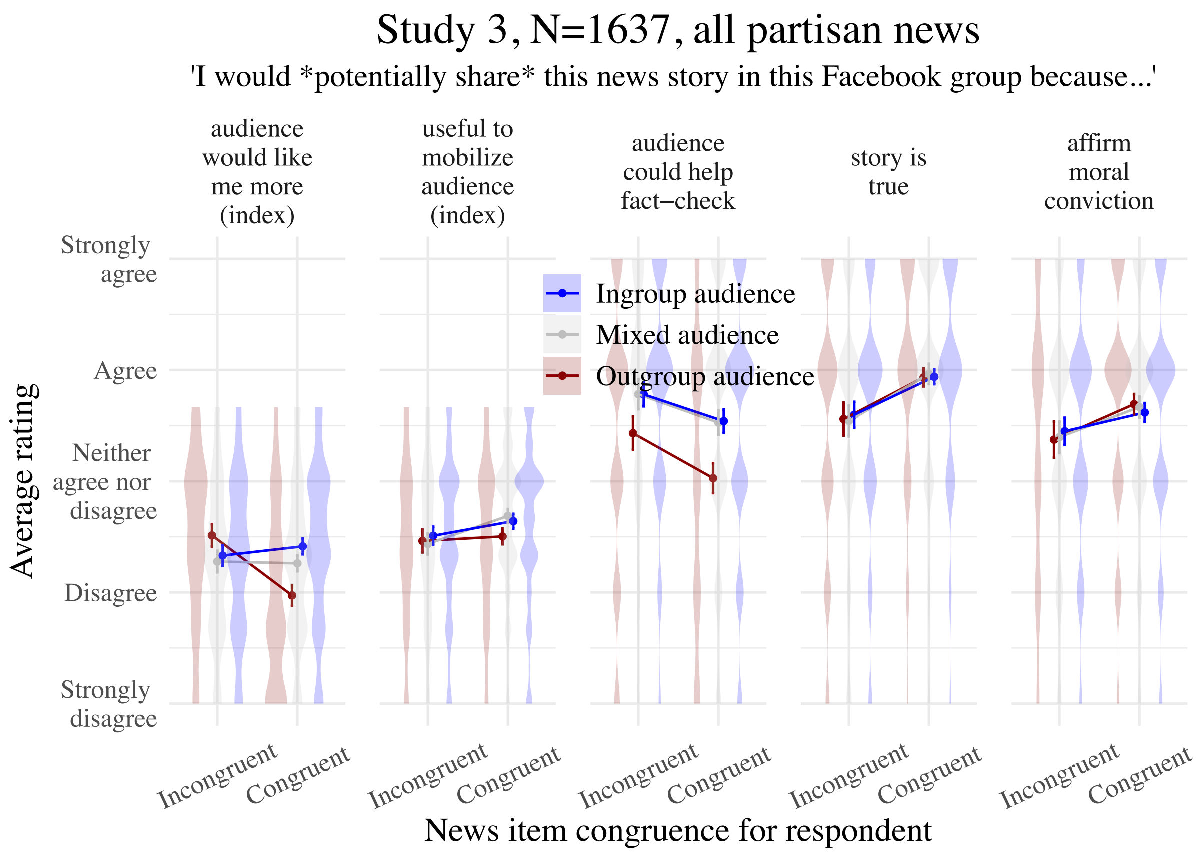

Supplementary figure 11.

H.7 Plots: motivations for sharing, breaking down by partisan news types

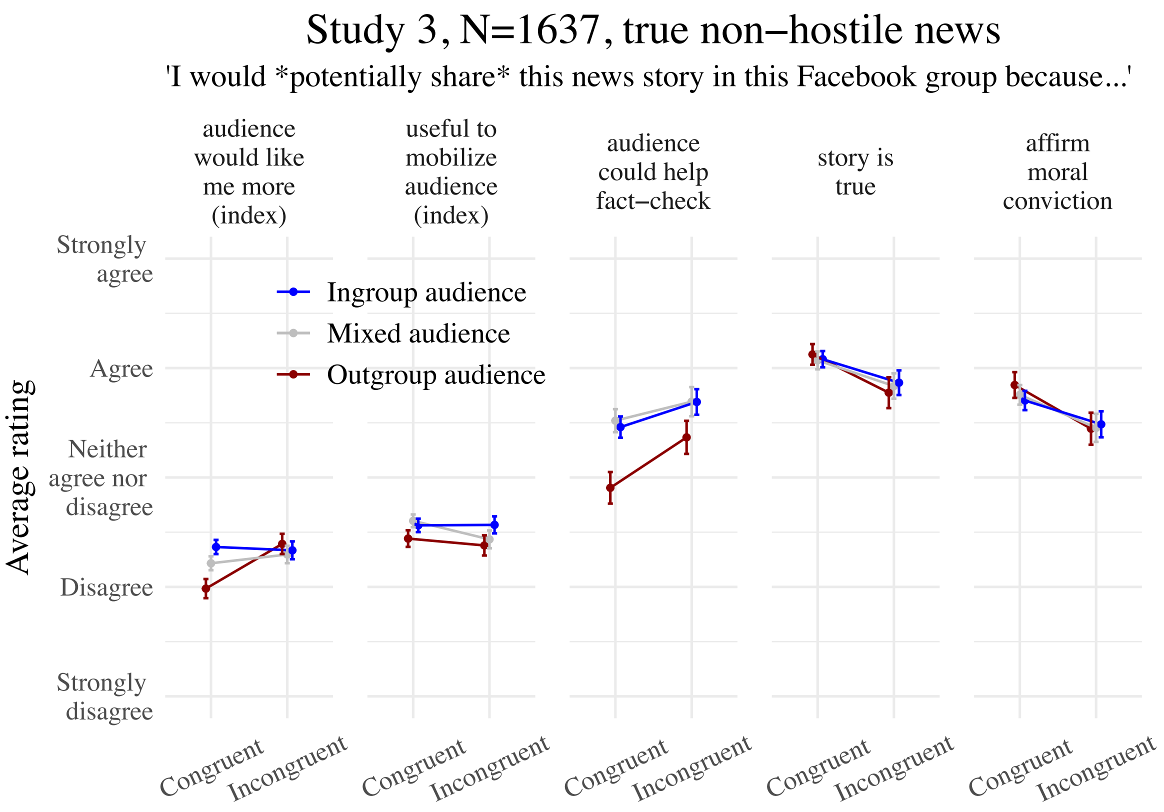

Supplementary figure 12: Motivations for sharing the true non-hostile news in Study 3 as a function of each motivation type. NB: graph produced for exploratory purposes. 95% confidence intervals around the mean were created from ggplot2 (not model summarise), so they do <u>not</u> take the nested structure of the data within participants into account, resulting in narrower confidence intervals.

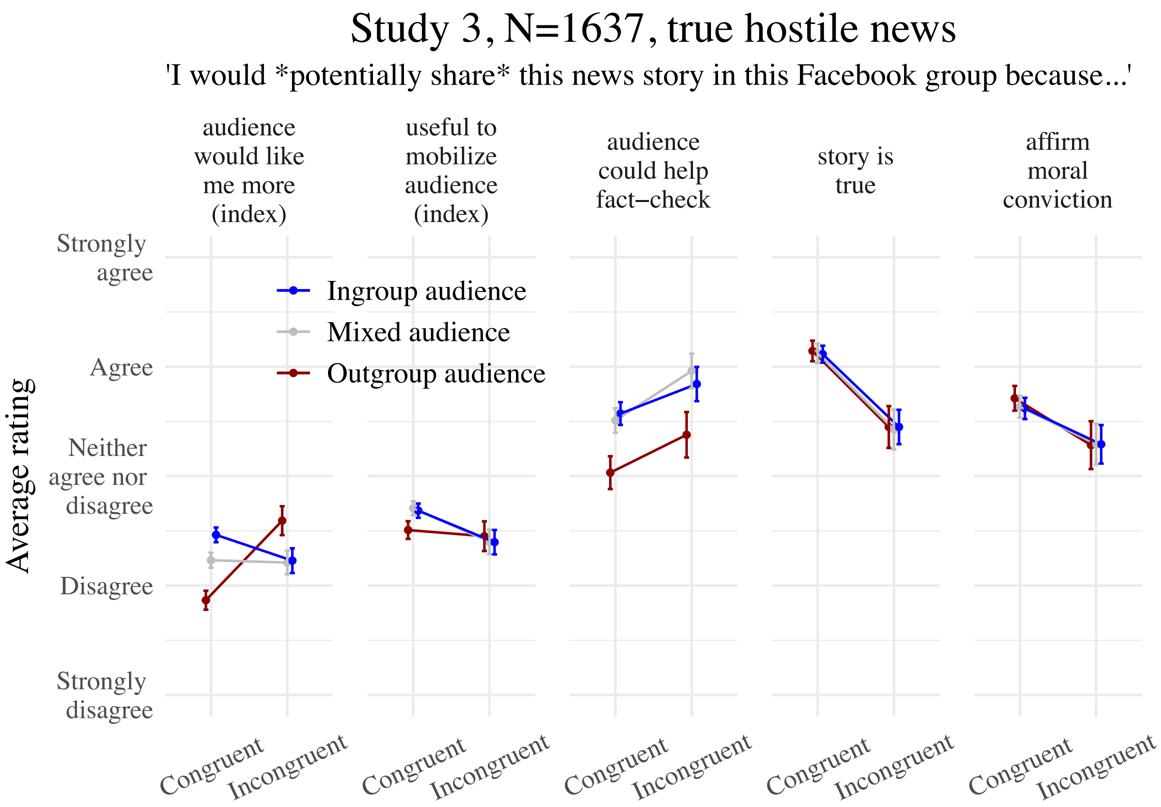

Supplementary figure 13: Motivations for sharing the true hostile news in Study 3 as a function of each motivation type. NB: graph produced for exploratory purposes. 95% confidence intervals around the mean were created from ggplot2 (not model summaries), so they do not take the nested structure of the data within participants into account, resulting in narrower confidence intervals.

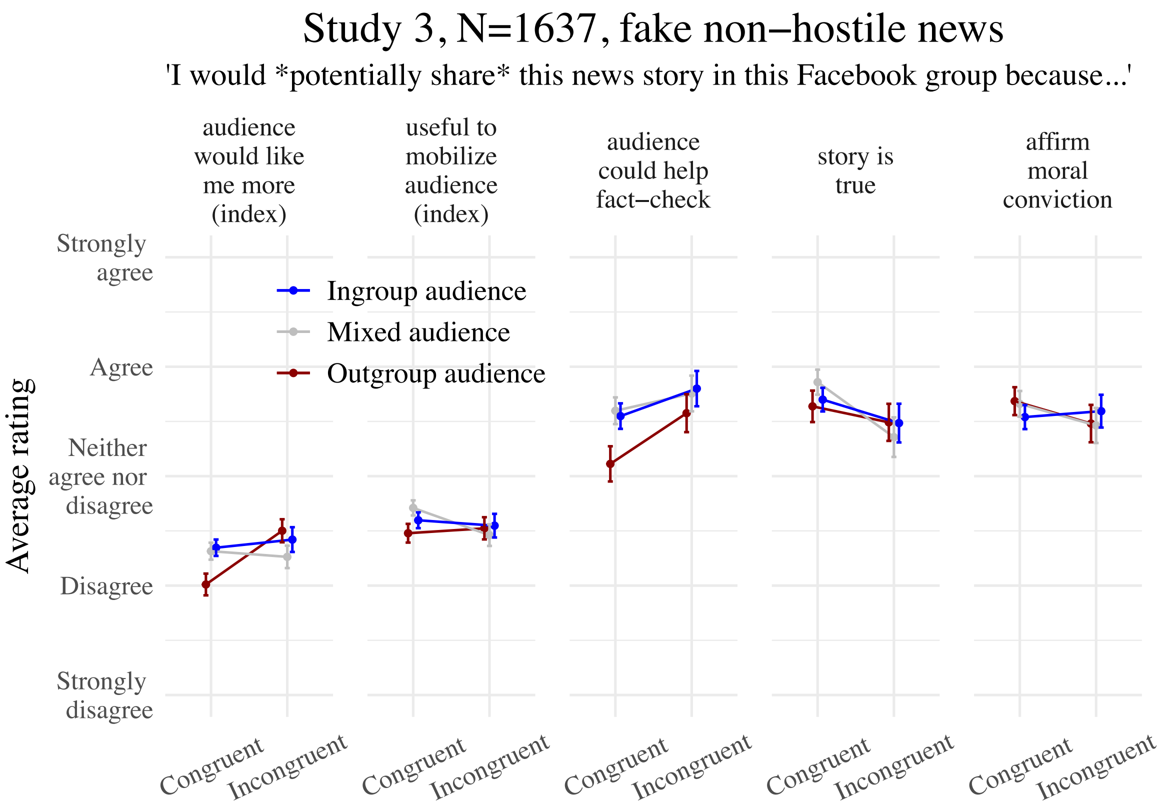

Supplementary figure 14: Motivations for sharing the false non-hostile news in Study 3 as a function of each motivation type. NB: graph produced for exploratory purposes. 95% confidence intervals around the mean were created from ggplot2 (not model summaries), so they do not take the nested structure of the data within participants into account, resulting in narrower confidence intervals.

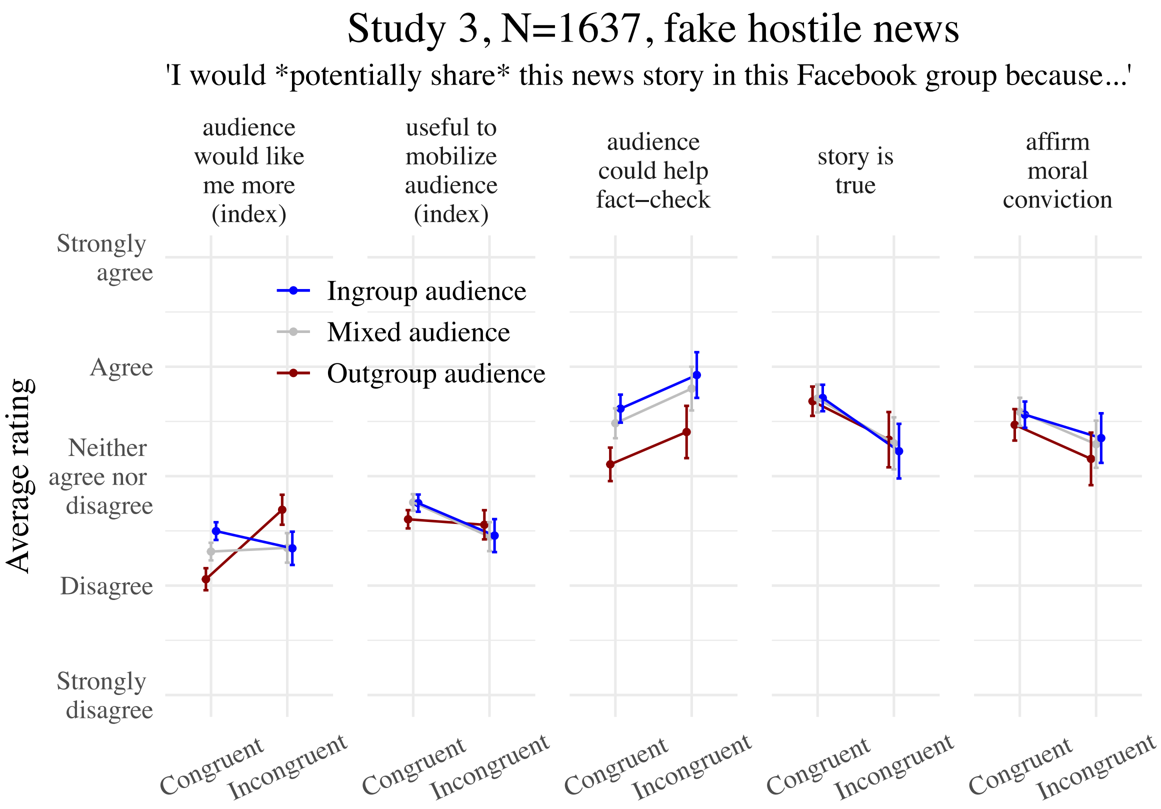

Supplementary figure 15: Motivations for sharing the false hostile news in Study 3 as a function of each motivation type. NB: graph produced for exploratory purposes. 95% confidence intervals around the mean were created from ggplot2 (not model summaries), so they do not take the nested structure of the data within participants into account, resulting in narrower confidence intervals.

H.8 Tests comparing motivations for not sharing

Those comparisons were made on the mean scores of each motivation for sharing (averaging across congruent and incongruent news and audience)
Baseline: 'Audience would like me less'

```
# A tibble: 5 × 7
  term                                          estimate std.error statistic   p.value conf.low conf.high
  <chr>                                            <dbl>     <dbl>     <dbl>     <dbl>    <dbl>     <dbl>
1 (Intercept)                                      2.29     0.0230      99.4 0            2.24      2.33
2 motivationuseful to mobilize audience (index)    0.285    0.0167      17.1 5.80e- 58    0.252     0.318
3 motivationaudience could help fact-check         1.21     0.0303      39.8 1.31e-213    1.15      1.27
4 motivationstory is true                          1.53     0.0301      50.9 2.45e-289    1.48      1.59
5 motivationaffirm moral conviction                1.29     0.0265      48.5 2.63e-273    1.24      1.34
```

H.9 Regressions: motivations for not sharing, averaging across all partisan news types (baseline: Outgroup audience)

Story is false

Stu 3 - mot not to share: story is false - averaging across all congruent partisan news

Predictors	Estimates	value CI	p
(Intercept)	2.91	2.83,2.98	**<0.001**
Audience InMixOut [Mixed audience]	0.14	0.03,0.25	**0.009**
Audience InMixOut [Ingroup audience]	0.16	0.04,0.28	**0.008**
Observations	8140		
R^2 / R^2 adjusted	0.003 / 0.003		

Stu 3 - mot not to share: story is false - averaging across all incongruent partisan news

Predictors	Estimates	value CI	p
(Intercept)	3.49	3.42,3.55	**<0.001**
Audience InMixOut [Mixed audience]	0.08	-0.01,0.17	0.092
Audience InMixOut [Ingroup audience]	0.04	-0.06,0.14	0.444
Observations	10248		
R^2 / R^2 adjusted	0.001 / 0.000		

Goes against moral conviction

Stu 3 - mot not to share: go against moral conviction - averaging across all congruent partisan news

Predictors	Estimates	value CI	p
(Intercept)	2.80	2.71,2.90	**<0.001**
Audience InMixOut [Mixed audience]	-0.07	-0.21,0.06	0.271

Audience InMixOut [Ingroup audience]	-0.06	-0.19,0.08	0.419
Observations	8140		
R^2 / R^2 adjusted	0.001 / 0.000		

Stu 3 - mot not to share: go against moral conviction - averaging across all incongruent partisan news

Predictors	*Estimates*	*CI*	**value** *p*
(Intercept)	3.14	3.06,3.22	**<0.001**
Audience InMixOut [Mixed audience]	-0.17	-0.28,-0.05	**0.005**
Audience InMixOut [Ingroup audience]	-0.16	-0.28,-0.04	**0.010**
Observations	10248		
R^2 / R^2 adjusted	0.004 / 0.004		

Audience would like me less

Stu 3 - mot not to share: audience would like me less (index) - averaging across all congruent partisan news

Predictors	*Estimates*	*CI*	**value** *p*
(Intercept)	2.33	2.26,2.39	**<0.001**
Audience InMixOut [Mixed audience]	-0.18	-0.27,-0.10	**<0.001**
Audience InMixOut [Ingroup audience]	-0.45	-0.53,-0.36	**<0.001**
Observations	8140		
R^2 / R^2 adjusted	0.060 / 0.060		

Stu 3 - mot not to share: audience would like me less (index) - averaging across all incongruent partisan news

			**value**

Predictors	*Estimates*	*CI*	*p*
(Intercept)	2.05	2.00,2.11	**<0.001**
Audience InMixOut [Mixed audience]	0.11	0.03,0.19	**0.005**
Audience InMixOut [Ingroup audience]	0.06	-0.01,0.14	0.109
Observations	10248		
R^2 / R^2 adjusted	0.004 / 0.004		

Not useful to mobilize audiences

Stu 3 - mot not to share: not useful to mobilize audience (index) - averaging across all congruent partisan news

		**value**	
Predictors	*Estimates*	*CI*	*p*
(Intercept)	2.43	2.37,2.49	**<0.001**
Audience InMixOut [Mixed audience]	-0.25	-0.33,-0.16	**<0.001**
Audience InMixOut [Ingroup audience]	-0.44	-0.52,-0.35	**<0.001**
Observations	8140		
R^2 / R^2 adjusted	0.061 / 0.061		

Stu 3 - mot not to share: not useful to mobilize audience (index) - averaging across all incongruent partisan news

		**value**	
Predictors	*Estimates*	*CI*	*p*
(Intercept)	2.43	2.38,2.49	**<0.001**
Audience InMixOut [Mixed audience]	-0.15	-0.23,-0.07	**<0.001**
Audience InMixOut [Ingroup audience]	-0.28	-0.37,-0.20	**<0.001**
Observations	10248		
R^2 / R^2 adjusted	0.024 / 0.024		

Audience could not help fact check

Stu 3 - mot not to share: audience cannot help fact-check - averaging across all congruent partisan news

		**value**	
Predictors	*Estimates*	*CI*	*p*
(Intercept)	3.08	2.98,3.17	**<0.001**
Audience InMixOut [Mixed audience]	-0.38	-0.51,-0.25	**<0.001**
Audience InMixOut [Ingroup audience]	-0.46	-0.59,-0.32	**<0.001**
Observations	8140		
R^2 / R^2 adjusted	0.029 / 0.029		

Stu 3 - mot not to share: audience cannot help fact-check - averaging across all incongruent partisan news

		**value**	
Predictors	*Estimates*	*CI*	*p*
(Intercept)	3.14	3.05,3.23	**<0.001**
Audience InMixOut [Mixed audience]	-0.37	-0.50,-0.25	**<0.001**
Audience InMixOut [Ingroup audience]	-0.57	-0.69,-0.44	**<0.001**
Observations	10248		
R^2 / R^2 adjusted	0.038 / 0.038		

H.10 Plot: motivations for not sharing supplemented with density plots for raw data

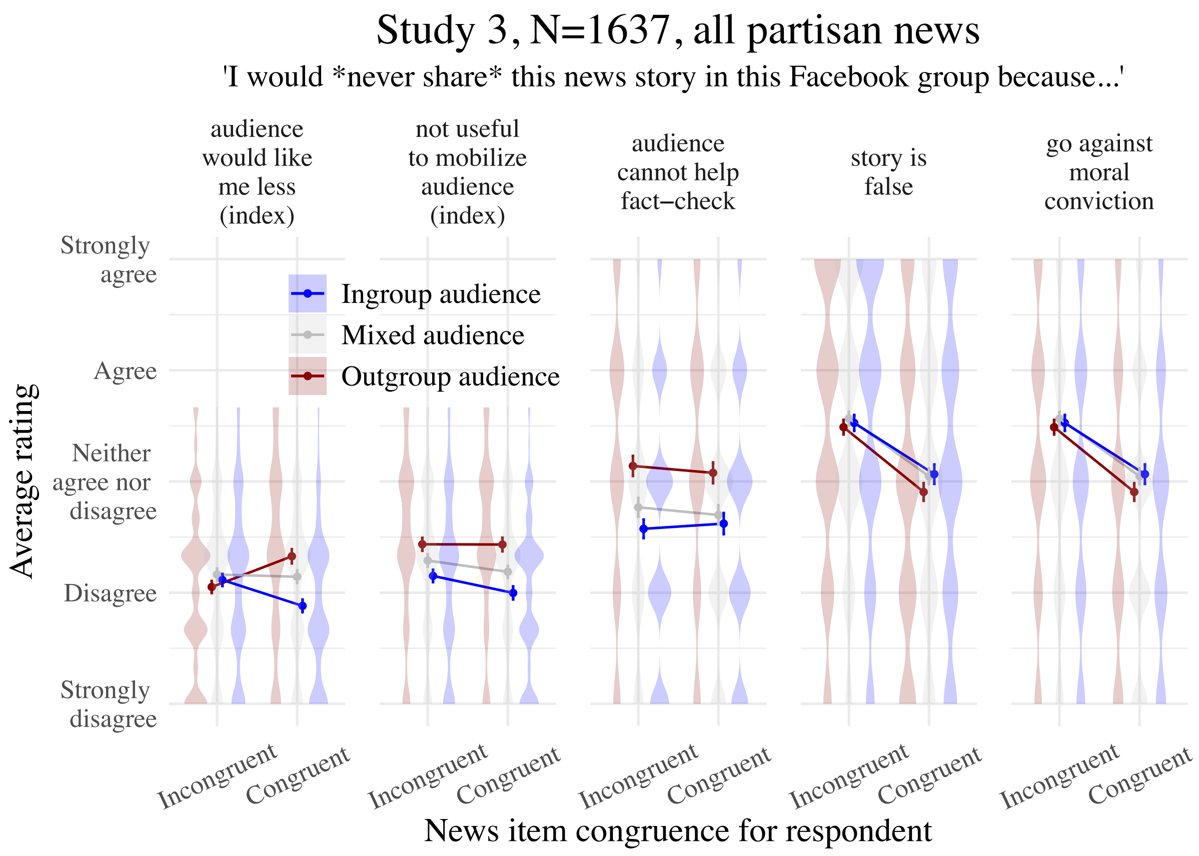

Supplementary figure 16.

H11. Plots: motivations for not sharing, breaking down by partisan news types

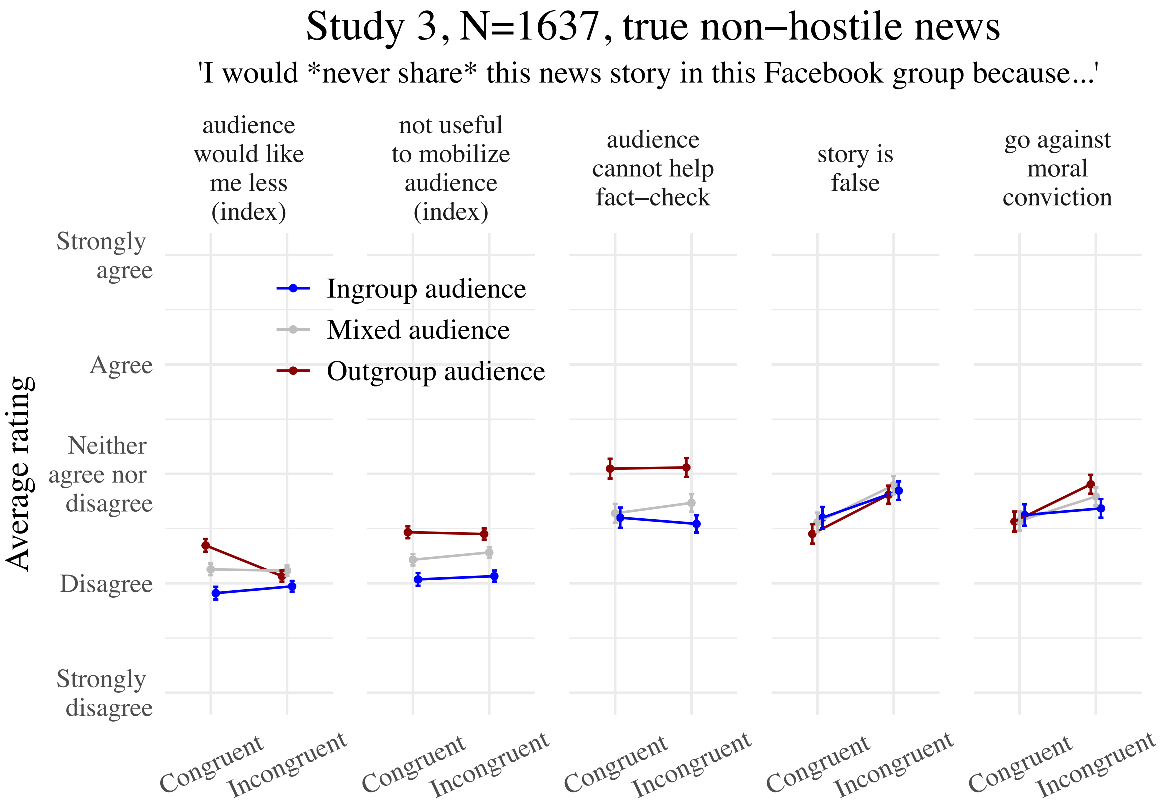

Supplementary figure 17: Motivations for not sharing the true non-hostile news in Study 3. NB: graph produced for exploratory purposes. 95% confidence intervals around the mean were created from ggplot2 (not model summaries), so they do not take the nested structure of the data within participants into account, resulting in narrower confidence intervals.

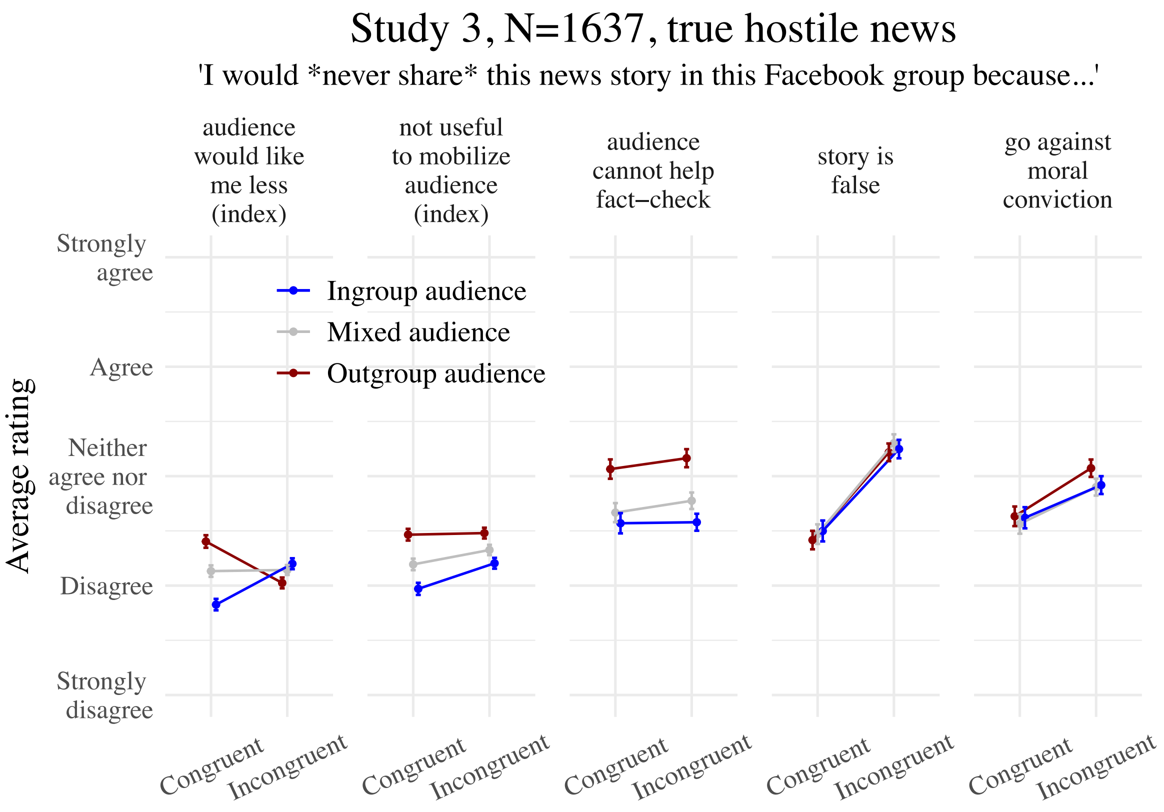

Supplementary figure 18: Motivations for not sharing the true hostile news in Study 3. NB: graph produced for exploratory purposes. 95% confidence intervals around the mean were created from ggplot2 (not model summaries), so they do not take the nested structure of the data within participants into account, resulting in narrower confidence intervals.

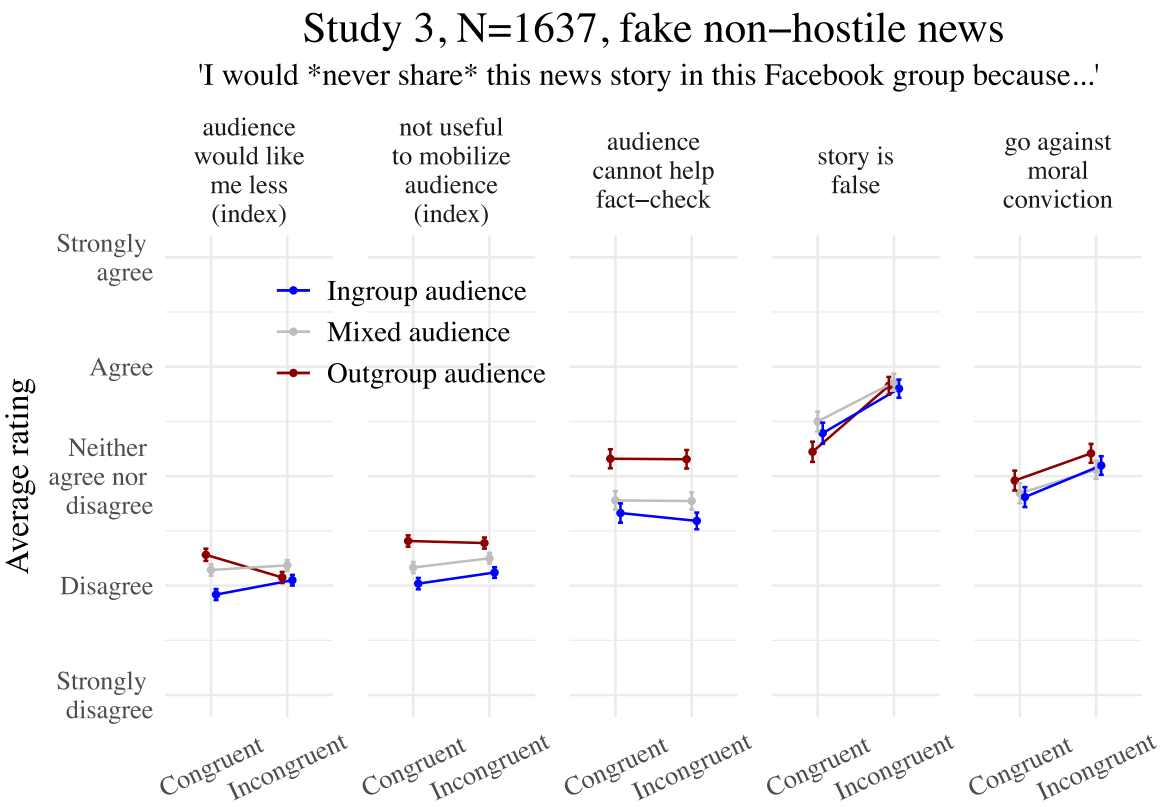

Supplementary figure 19: Motivations for not sharing the false non-hostile news in Study 3. NB: graph produced for exploratory purposes. 95% confidence intervals around the mean were created from ggplot2 (not model summaries), so they do not take the nested structure of the data within participants into account, resulting in narrower confidence intervals.

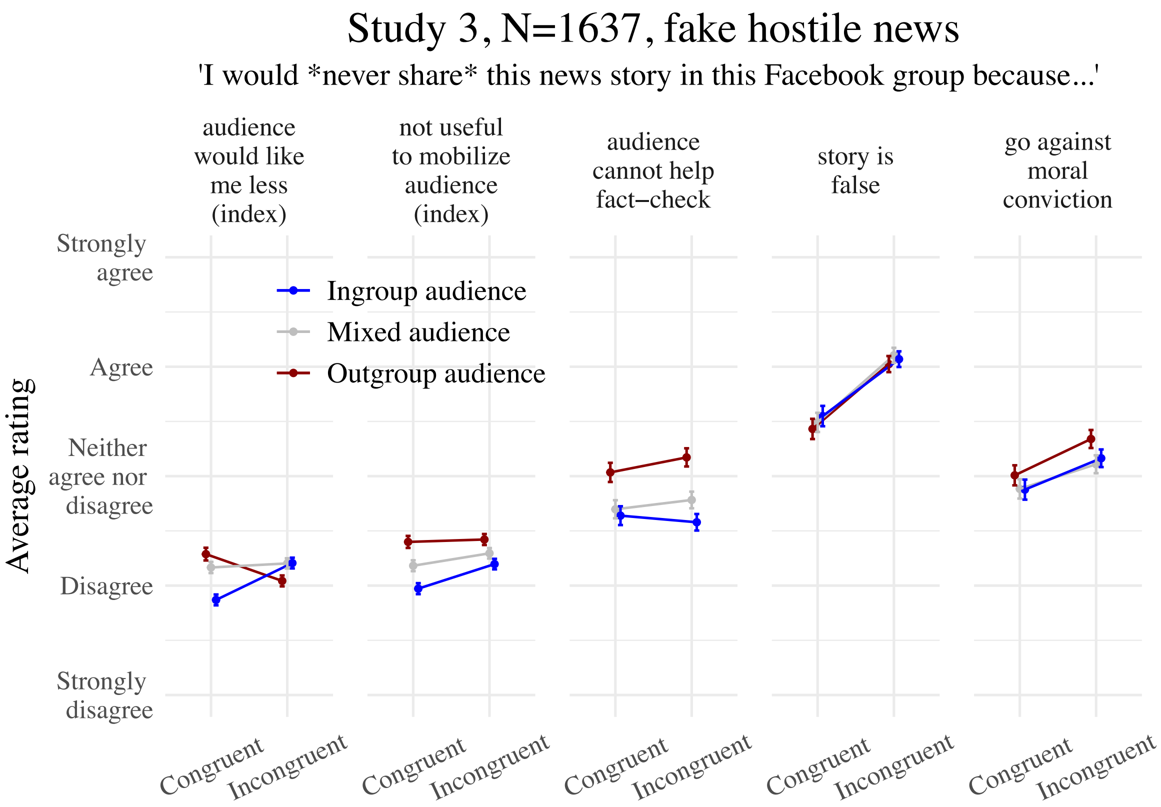

Supplementary figure 20: Motivations for not sharing the false hostile news in Study 3. NB: graph produced for exploratory purposes. 95% confidence intervals around the mean were created from ggplot2 (not model summaries), so they do not take the nested structure of the data within participants into account, resulting in narrower confidence intervals.
